# Multiple myeloma-associated *hDIS3* mutations cause perturbations in cellular RNA metabolism and suggest hDIS3 PIN domain as a potential drug target

**DOI:** 10.1093/nar/gkt930

**Published:** 2013-10-21

**Authors:** Rafal Tomecki, Karolina Drazkowska, Iwo Kucinski, Krystian Stodus, Roman J. Szczesny, Jakub Gruchota, Ewelina P. Owczarek, Katarzyna Kalisiak, Andrzej Dziembowski

**Affiliations:** ^1^Department of Biophysics, Institute of Biochemistry and Biophysics, Polish Academy of Sciences, Pawinskiego 5a, 02-106 Warsaw, Poland, ^2^Institute of Genetics and Biotechnology, Faculty of Biology, University of Warsaw, Pawinskiego 5a, 02-106 Warsaw, Poland and ^3^International Institute of Molecular and Cell Biology, Trojdena 4, 02-109 Warsaw, Poland

## Abstract

hDIS3 is a mainly nuclear, catalytic subunit of the human exosome complex, containing exonucleolytic (RNB) and endonucleolytic (PIN) active domains. Mutations in *hDIS3* have been found in ∼10% of patients with multiple myeloma (MM). Here, we show that these mutations interfere with hDIS3 exonucleolytic activity. Yeast harboring corresponding mutations in *DIS3* show growth inhibition and changes in nuclear RNA metabolism typical for exosome dysfunction. Construction of a conditional *DIS3* knockout in the chicken DT40 cell line revealed that *DIS3* is essential for cell survival, indicating that its function cannot be replaced by other exosome-associated nucleases: hDIS3L and hRRP6. Moreover, HEK293-derived cells, in which depletion of endogenous wild-type *hDIS3* was complemented with exogenously expressed MM hDIS3 mutants, proliferate at a slower rate and exhibit aberrant RNA metabolism. Importantly, MM mutations are synthetically lethal with the hDIS3 PIN domain catalytic mutation both in yeast and human cells. Since mutations in PIN domain alone have little effect on cell physiology, our results predict the hDIS3 PIN domain as a potential drug target for MM patients with *hDIS3* mutations. It is an interesting example of intramolecular synthetic lethality with putative therapeutic potential in humans.

## INTRODUCTION

Multiple myeloma (MM) is a lethal neoplastic disease accounting for 10–15% of hematologic malignances and 20% of deaths related to cancer of the blood and bone marrow (1). MM originates from terminally differentiated antibody-producing B cells known as plasma cells (1).

The genetic background of MM is not completely understood. Hypermutations occurring at the time of immunoglobulin receptor affinity maturation and class switching are involved in MM pathogenesis, leading to chromosomal abnormalities such as translocations, hyperdiploidy, hypodiploidy, monosomy or partial deletion of chromosome 13 (1–3). A recent whole-genome sequencing of 38 MM patients provided a global view on the somatic mutations associated with this cancer (4). Unexpectedly, *hDIS3* gene was mutated in ∼10% of MM patients (4). Importantly, these mutations were either homo- or hemizygotic. A high frequency of *hDIS3* gene mutations in MM patients was recently confirmed in another high-throughput study (5). Interestingly, *hDIS3* gene mutations were also found in global screens of other cancers, such as medulloblastoma and acute myeloid leukemia (6,7). Additionally, *hDIS3* was identified in transcriptomic analyses as one of the genes, whose expression differentiates superficial spreading melanoma from nodular melanoma (8). Furthermore, *hDIS3* overexpression was earlier observed in human colorectal cancer and in a mouse model of this cancer, where elevated levels of respective mRNA and protein positively correlated with the incidence of metastasis (9,10). Expression profiling revealed that *hDIS3* is among several genes whose loss-of-function significantly reduces viability of colorectal carcinoma cell lines (11). Increased levels of hDIS3 mRNA have been also recently proposed as one of the characteristics of the epithelial ovarian cancer (12). All examples presented above strongly suggest the existence of possible molecular link between hDIS3 functions and development of different cancers [reviewed in (13)]. More specifically, it appears likely that exonucleolytic activity of hDIS3 protein—the major catalytic subunit of the exosome—might be somehow involved in this association.

hDIS3 is a catalytic subunit of the RNA exosome, which plays a crucial role in RNA processing and decay. The exosome complex has an evolutionarily conserved structure encompassing a 9-subunit ring devoid of any catalytic activity (14,15). The associated ribonucleases responsible for the enzymatic activity of the exosome belong to two different families: Dis3 proteins, similar to bacterial RNases II/R, and Rrp6 proteins, members of the RNase D family (16). In yeast, single genes code for Dis3 and Rrp6 proteins. Dis3 is the only essential catalytic subunit, present both in the nucleus and cytoplasm, while Rrp6 is restricted to the nucleus and responsible for only a subset of nuclear exosome functions (17). Dis3 is a multidomain protein with two different catalytic activities: a 3′–5′ exonucleolytic activity via the RNase II/R (RNB) domain and an endonucleolytic activity via the PilT N-terminal (PIN) domain at the N-terminus (16,18–20). The Dis3 exonuclease active site is located near the bottom of the central channel of the 9-subunit ring through which substrates are delivered (21–25). Both activities cooperate with each other, but the exonucleolytic activity is more important for cell physiology, whereas mutations abolishing the endonucleolytic activity alone have no detectable growth phenotype (18–20,24).

The human genome encodes three Dis3 homologues, of which only hDIS3 and hDIS3L were found to associate with the exosome (26,27). Notably, both of them are processive 3′–5′ hydrolytic exonucleases, whereas only hDIS3 has also retained endonuclease activity in its PIN domain. *In vivo* localization studies and analyses of substrate specificities revealed that hDIS3L is restricted to the cytoplasmic exosome, whereas hDIS3 is mainly a nucleoplasmic protein, with a small fraction present in the cytoplasm (26,27). Additionally, human RRP6 is mainly nuclear and significantly enriched in the nucleoli, with a minor fraction in the cytoplasm (26). Thus, human RNA exosomes, although based on the same structural scaffold as their *Saccharomyces cerevisiae* counterparts, exist as functionally and compositionally distinct variants in different areas of the nucleus and in the cytoplasm (28).

The third human Dis3 homologue—hDIS3L2—does not interact with the exosome core, due to the lack of PIN domain and it has been recently demonstrated by several independent studies that it is responsible for an alternative 3′–5′ RNA decay pathway in the cytoplasm (29–31). An important role of hDIS3L2 in the maintenance of proper cellular metabolism is supported by the fact that mutations in its gene were found to be associated with Perlman syndrome of overgrowth and predisposition to Wilm’s tumor development (13,32).

The exosome plays a fundamental role in most RNA metabolic pathways (16,33,34). These pathways include turnover of normal mRNAs and AU-rich element-regulated decay of unstable mRNAs (35,36); nuclear processing of stable RNA classes (small nuclear RNA (snRNA), small nucleolar RNA (snoRNA), rRNA, tRNA) (17,37); degradation of unstable transcripts arising from intergenic regions (38–40); and complete degradation of mRNA after endonucleolytic cleavage initiated by small interfering RNA (siRNA) in RNA interference (41). Furthermore, the exosome plays a crucial role in the quality control, as it is a primary enzyme degrading unwanted molecules in the nucleus and the cytoplasm, including incorrectly processed pre-mRNAs, rRNAs and tRNAs as well as translationally incompetent mRNAs (17,37,42–47). Apart from its well-documented role in RNA metabolism, a recent study has implicated the exosome in the activation-induced immunoglobulin heavy-chain class switch recombination and immunoglobulin variable region somatic hypermutation in human B lymphocytes (48). It was also suggested that the exosome is essential for activation-induced cytidine deaminase (AID) recruitment to chromatin (48), but the mechanistic details of this process remain elusive.

Here, we explored the consequences of *hDIS3* gene mutations found in MM patients using biochemical, genetic and functional analyses, and examined possible synthetic genetic interactions between hDIS3 exonucleolytic activity and other catalytic activities of the nuclear exosome.

## MATERIALS AND METHODS

### Cell culture and generation of stable cell lines

Human HEK293 Flp-In T-REx (Invitrogen) cells were cultured as monolayers in Dulbecco’s modified Eagle’s medium (D-MEM, Gibco) supplemented with 10% tetracycline-free fetal bovine serum (TET System Approved Fetal Bovine Serum (FBS); Clontech) and antibiotics (penicillin-streptomycin; Sigma-Aldrich) at 37°C in a 5% CO_2_ humidified atmosphere.

The stable inducible HEK293 Flp-In T-REx cell lines were obtained in this study using highly pure DNA midipreps of pMM7-pMM14 plasmid constructs (Supplementary Table S3; see Supplementary Materials and Methods for details on construction of the plasmids) and the Flp-In™ T-REx™ system (Invitrogen), according to the protocol of the manufacturer. Established cell lines were grown in the same medium as above, supplemented with hygromycin B (100 μg/ml) and blasticidin (10–15 μg/ml) (both from Invitrogen). Transfections were performed with Lipofectamine2000 (Invitrogen). Expression of exogenous genes was induced by addition of doxycycline to the culture medium at a final concentration of 100 ng/ml.

The chicken DT40 Cre1 cell line was obtained from the laboratory of Jean-Marie Buerstedde (Institute for Molecular Radiobiology, Neuherberg-Munich, Germany). DT40 cells were cultured in D-MEM supplemented with 10% FBS (Invitrogen), 1% chicken serum (Sigma-Aldrich), 0.1 mM 2-mercaptoethanol (Sigma-Aldrich), 100 U/ml of penicillin and 0.1 mg/ml of streptomycin (Sigma-Aldrich) at 37°C in a 5% CO_2_ humidified atmosphere. Cell densities were kept between 0.2 and 2 million per 1 ml.

Stable integration of pMM15 and pMM16 constructs (Supplementary Table S3; see Supplementary Materials and Methods for details on construction of the plasmids) into the DT40 *DIS3* locus with the aim of generating conditional knockouts was performed as follows. DT40 Cre1 cells (10^7^) were centrifuged, resuspended in 0.8 ml of growth medium and incubated on ice for 10 min with 40 μg of the respective plasmid linearized with *Not*I restriction enzyme. The cells were then electroporated in ice-chilled 0.4 cm cuvettes (Biorad) using the following parameters: 700 V, 25 μF. The cells were transferred into 10 ml of medium and cultured for 24 h in small flasks. Ten milliliters of medium with the proper selection antibiotic was added (puromycin or blasticidin S, both from Invivogen; final concentrations of 0.5 μg/ml and 20 μg/ml, respectively), and cells were transferred to 96-well plates (200 μl each well). After 7–14 days, single colonies were counted under the microscope and resuspended in 1.5 ml of fresh medium containing selection antibiotics. Surviving cells were collected for further analysis. The stable heterozygotic puromycin-resistant DT40 Cre1 cell line (*dis3Δ^PuroR^/DIS3*) was generated following one round of transfection with the pMM15 construct. To obtain the stable homozygotic puromycin- and blastidicin S-resistant DT40 Cre1 cell line (*dis3Δ^PuroR^/dis3Δ^BsrR^*), a second round of electroporation was performed using the pMM16 construct.

The conditional *DIS3* knockout in DT40 Cre1-derived cell lines obtained above was achieved through Cre-mediated *LoxP* excision of the *hDIS3* expression cassette induced by 4-hydroxy-tamoxifen. Briefly, cells (10^6^) were cultured for 48 h in presence of 10 μM 4-hydroxy-tamoxifen (Sigma-Aldrich). Cells were counted and subcloned in a set of dilutions to obtain single colonies on 96-well plates. After 5–7 days, colonies were counted under the microscope, and cells were resuspended and split into two media: with or without selection antibiotic(s). Colonies that did not survive exposure to antibiotic(s) were treated as cultures with complete *LoxP* excision.

### Cell growth analyses

Stable inducible human HEK293 Flp-In T-REx cell lines were grown as described above in 100 mm plates until reaching 90% confluence. In the 48-h induction mode, 2 × 10^5^ cells were plated directly in six-well plates in medium either lacking or containing doxycycline, and grown for another 48 h. In the 48-h + 48-h induction mode, the cells were first replated in new 100 mm plates in medium either lacking or containing doxycycline at 40% confluence. After 48 h, 2 × 10^5^ cells were plated in six-well plates in the respective medium and grown for another 48 h. Cell growth and eGFP fluorescence were analyzed using an Olympus IX81 microscope.

Metabolic activity assays of the cell lines were performed as follows. Cells (5 × 10^3^) were plated in triplicate (for each cell line, condition and time of measurement) in 96-well plates. Cells were either maintained in medium lacking doxycycline or subjected to treatment with the inducer. At various time points following induction (24, 72 or 120 h), 10 μl of AlamarBlue® (Invitrogen) was added to the cultures. Amounts of the reduced reagent were quantified after 120 min using the DTX880 Multimode Detector (Beckman Coulter).

### Western blotting

Protein samples from yeast cultures and human or chicken cell lines were prepared according to standard protocols, run on 10–12% sodium dodecyl sulphate-polyacrylamide gel electrophoresis (SDS-PAGE) gels and immobilized on Protran nitrocellulose membranes (Whatman) by electrotransfer using Trans-Blot® SD Semi-Dry Transfer Cell (Bio-Rad). Following transfer, membranes were stained with Ponceau S Red (Sigma-Aldrich; 0.1% in 3% acetic acid), blocked in 5% nonfat milk in Tris-buffered saline (TBS) containing 0.05% Tween-20 (TBST), and incubated in the same solution with primary antibodies. The following primary antibodies were used for analyses: mouse monoclonal anti-eGFP (B2) (Santa Cruz Biotechnology; sc-9996) (1:1000), rabbit polyclonal anti-FLAG (Sigma-Aldrich; F-7425) (1:3000), rabbit polyclonal anti-hDIS3 (Sigma-Aldrich; HPA039281, lot: R37348) (1:1500), rabbit polyclonal anti-hDIS3L (Sigma-Aldrich; HPA041805, lot: R38591) (1:500), rabbit polyclonal anti-hRRP6 (Sigma-Aldrich; P4124) (1:3000), rabbit polyclonal anti-hDIS3L2 (31) (1:2000) and goat polyclonal anti-glyceraldehyde 3-phosphate dehydrogenase (GAPDH) (V-18) (Santa Cruz Biotechnology; sc-20357) (1:2000). Membranes were then washed with TBST and incubated with appropriate secondary antibody [goat anti-mouse, goat anti-rabbit (Calbiochem; 401215, 401393, respectively) or rabbit anti-goat (Sigma-Aldrich; A5420)] conjugated with horseradish peroxidase. Protein A tag was detected directly using rabbit Peroxidase–Anti-Peroxidase Soluble Complex antibody (Sigma-Aldrich; P1291) (1:3000). Blots were developed in a Curix 60 machine (AGFA) using the Immun-Star^TM^ WesternC^TM^ Kit (Bio-Rad) and CL-XPosure^TM^ Films (Thermo Scientific).

### Heterologous expression and purification of hDIS3 proteins

For purification of the panel of hDIS3 variants, the *Escherichia coli* BL21-CodonPlus-RIL strain (Stratagene) was first transformed with appropriate plasmids (pHEX1, pHEX8 or pMM1-pMM6). Transformants were grown at 18°C for 48 h in 1 l of Auto Induction Media Super Broth Base Including Trace Elements (Formedium) supplemented with 2% glycerol, kanamycin (30 μg/ml) and chloramphenicol (34 μg/ml), following inoculation from a pre-culture, grown in standard Luria-Broth (LB) medium containing both antibiotics. Recombinant proteins were purified as described previously (26,49), with minor modifications.

### Exoribonuclease assays

*In vitro* enzymatic assays of hDIS3 exoribonucleolytic activity were performed in 20 μl reaction volumes containing 10 mM Tris–HCl, pH = 8.0, 75 mM NaCl, 1 mM 2-mercaptoethanol and 100 μM MgCl_2_. Protein concentration was 0.1 μM, while substrate concentration was 0.2 or 2 μM for duplex or unstructured RNA molecules, respectively. Details on the preparation of radiolabeled substrates can be found in the Supplementary Materials and Methods.

For gel-based analyses, reactions were performed at 37°C for the indicated times and terminated by adding 20 μl of formamide loading dye (90% formamide, 20 mM EDTA, 0.03% bromophenol blue, 0.03% xylene cyanol in 1 × Tris-borate-EDTA (TBE)). Reaction products were resolved in denaturing 20% polyacrylamide, 8 M urea, 1× TBE gels. In the case of thin layer chromatography (TLC)-based experiments, samples were collected by mixing aliquots of the reaction mixtures with 1 μl of 0.5 M EDTA, and subsequently analyzed by running them on polyethylenimine (PEI)-cellulose plates (Schleicher & Schuell) in 0.5 M LiCl/1 M formic acid using nonradioactive uridine monophosphate (UMP) as a marker. For both types of analyses, reaction products were visualized using a FUJI PhosphorImager.

### Southern hybridization

Approximately 10 μg of genomic DNA isolated from DT40-derived cell lines was digested overnight with 40 U of either *Nde*I or *Bsa*I restriction enzyme (both from ThermoScientific) in a total volume of 100 μl, according to the manufacturer’s instructions. Digested DNA was precipitated according to standard laboratory procedures and loaded onto a 0.6% agarose gel stained with ethidium bromide. Electrophoresis was performed in 1×Tris-acetate-EDTA (TAE) buffer at 8°C. Following DNA separation, gel was sequentially submerged for 30 min with gentle agitation in (i) depurination solution (0.25 M HCl), (ii) denaturation solution (1.5 M NaCl, 0.5 M NaOH) and (iii) neutralization buffer (1.5 M NaCl, 0.5 M Tris–HCl, pH 7.5). The gel was briefly rinsed with H_2_O between different steps of incubation described above. Finally, DNA was transferred overnight onto a Hybond N^+^ membrane (GE Healthcare), according to the manufacturer’s instructions, and fixed by ultraviolet cross-linking.

The polymerase chain reaction (PCR) product spanning a 480-bp fragment of the chicken *DIS3* right arm was amplified from the pMM15 construct with the DIS3_RF-DIS3rfinR primer pair (see Supplementary Table S2 for sequences). The amplification product was gel-purified and used as a probe for hybridization. Approximately 25 ng of PCR product was labeled with α-^32^P[dATP] using the DECAprime™ II Kit (Ambion) according to the manufacturer’s instructions. After denaturation and snap-cooling, labeled probe was purified using Micro Bio-Spin Column P-30 Tris (Biorad). Hybridization was performed at 68°C overnight in PerfectHyb Plus hybridization buffer (Sigma-Aldrich). The membrane was washed twice with 2×saline-sodium citrate (SSC), 0.1% SDS and once in 0.5×SSC, 0.1% SDS at 68°C for 20 min, and eventually exposed to a PhosphorImager screen (FujiFilm), which was scanned following exposure using a FLA 7000 scanner (FujiFilm).

### RNA isolation and northern-blot analysis

RNA was isolated from yeast cultures and human cell lines using the standard hot acidic phenol procedure or TRI Reagent (Sigma-Aldrich), respectively. Total RNA (5–10 μg) was fractionated by electrophoresis either in denaturing 6% polyacrylamide-urea gel followed by electroblotting onto a Hybond N^+^ membrane in 0.5× TBE at 4°C, or in a 1% formaldehyde-agarose gel, followed by RNA immobilization on the same type of membrane by overnight capillary transfer in 20× SSC (3 M NaCl, 0.3 M sodium citrate). RNA was fixed on membranes by ultraviolet cross-linking. Hybridizations were performed in PerfectHyb Plus hybridization buffer. The blots were handled according to standard procedures and probed at 42°C (5′-labeled oligonucleotide probes) or 63°C (DNA fragments labeled by random priming). Between successive hybridizations, probes were stripped off the membranes at 65°C using boiling 0.1% SDS.

For detection of yeast 5′-external transcribed spacer (ETS) and NEL025 cryptic unstable transcript (CUT), PCR probes labeled by random priming with α-^32^P[dATP] and DecaLabel DNA Labeling Kit (Thermo Scientific) as described previously (18) were used. Randomly primed PCR products, obtained with the primer pairs GAPDH_F-GAPDH_R and 7SL_F-7SL-R (Supplementary Table S2), were used as probes for detection of *GAPDH* mRNA and *7SL* RNA, respectively. Randomly primed probe, generated as previously described (26), was used for detection of the *hDIS3* transcript. Randomly primed DNA probe specific to histone H2A mRNA was generated by *Eco*RI/*Hin*dIII-mediated excision of sequence corresponding to its full-length CDS (GenBank ID: AY131971.1), cloned between respective sites of pUC19; the construct was kindly provided by Prof. Zbigniew Dominski. For all other transcripts, ^32^P-labeled oligonucleotides (listed in Supplementary Table S2) were used as probes. After hybridization, membranes were washed with 2× SSC, 0.1% SDS and results were visualized using phosphorimaging as described in the previous section. Quantification was performed using Multi Gauge v. 3.0 software (FujiFilm).

### Reverse transcription and quantitative PCR

Total RNA (10 μg) extracted from stable human cell lines was treated with 6 U of TURBO^TM^ DNase (Ambion) in the presence of RiboLock^TM^ RNase Inhibitor (Thermo Scientific), according to the manufacturer’s instructions. Following phenol:chloroform extraction and precipitation of RNA with isopropanol, 2 μg of DNase-treated RNA was reverse transcribed using a mixture of 50 pmol of an oligo(dT) primer and 250 ng of random hexamers (Invitrogen) and Superscript III^TM^ reverse transcriptase (Invitrogen), according to the manufacturer’s instructions, in a final volume of 20 µl. A portion (1/100) of the cDNA reaction was mixed with Platinum® Quantitative PCR SuperMix-UDG (Invitrogen), 2.5 pmol of each primer (Supplementary Table S2) and 0.3 μg of bovine serum albumin in a final volume of 10 µl and analyzed by real-time PCR in a Roche LightCycler® 480 system using an annealing temperature of 58°C. Negative controls lacking reverse transcriptase showed a negligible background. Analyses were performed in triplicate. All data were normalized to GAPDH mRNA.

## RESULTS

### hDIS3 proteins with MM-associated mutations display various defects in the degradation of different RNA substrates *in vitro*

**Figure 1.**
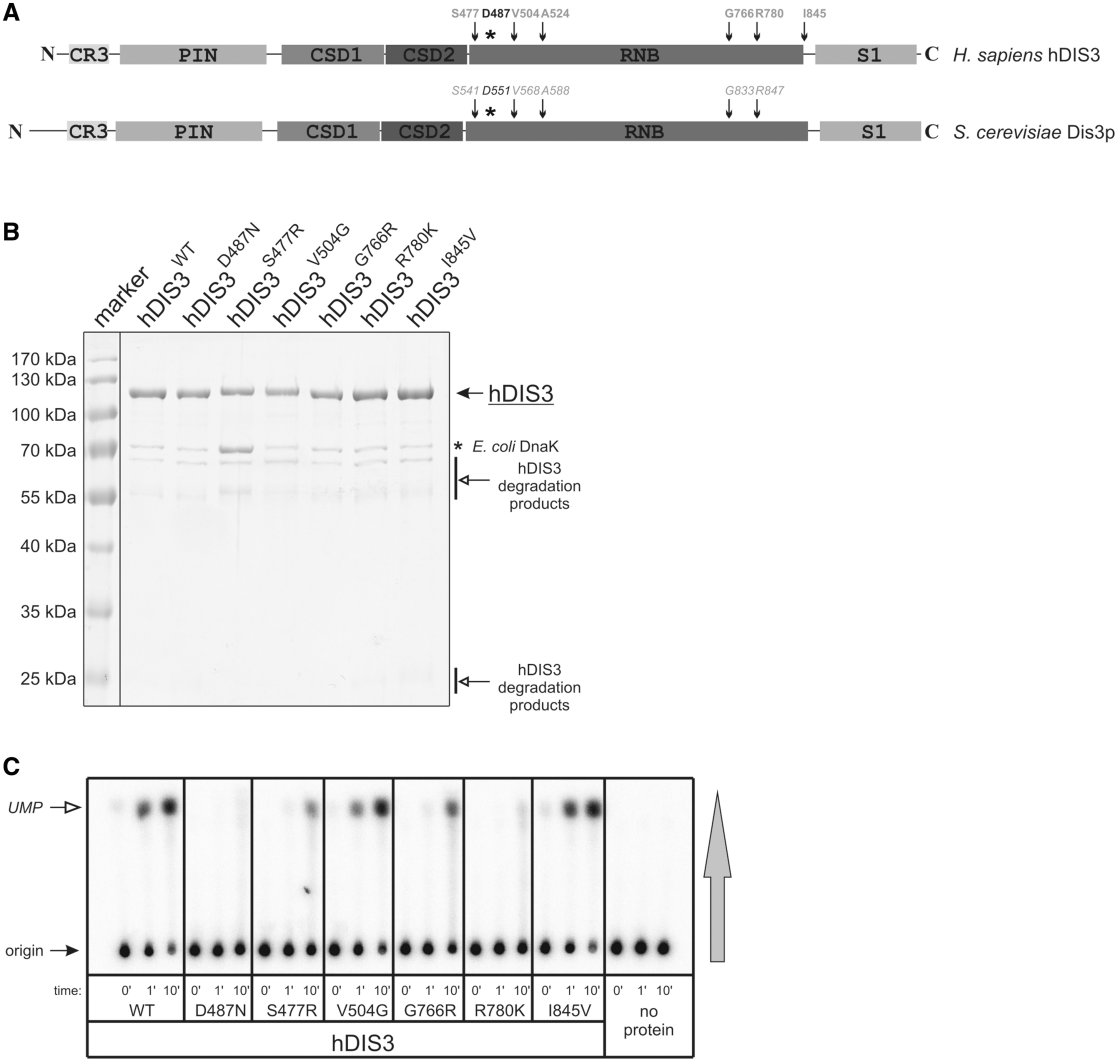
Substitutions of conserved amino acids in the hDIS3 RNB domain decrease enzymatic activity. (**A**) Schematic view of domain organization of human and yeast DIS3 proteins. Amino acids substituted in MM are indicated in light gray and their positions with respect to hDIS3 domains are marked with arrows. An asterisk indicates position of D487, which was previously shown to be critical for exoribonucleolytic activity of hDIS3. Corresponding amino acids in *S. cerevisiae* Dis3p are marked with *italics*. (**B**) SDS-PAGE analysis of hDIS3 protein variants purified using two-step affinity chromatography, followed by gel filtration. Position of hDIS3 protein is marked with a solid arrow. Common contaminations with bacterial DnaK chaperone and hDIS3 proteolytic degradation products are indicated with an asterisk and open arrows, respectively. The PageRuler^TM^ Prestained Protein Ladder (Fermentas) molecular marker is in the leftmost lane. (**C**) TLC analysis of the RNA degradation efficiency for recombinant hDIS3 proteins, shown in (B). Internally radiolabeled RNA, synthesized by *in vitro* transcription in the presence of [α-^32^P]UTP, was incubated with equal amounts of hDIS3 variants or in the absence of protein. Aliquots were collected at indicated time points and spotted onto a PEI-cellulose TLC plate, which was developed in the direction shown with the vertical gray arrow; positions of substrate and product (UMP) are indicated with solid and open arrows, respectively. S477R, G766R and R780K mutations significantly inhibit ribonucleolytic degradation.

The majority of mutations found in MM affect conserved residues in the hDIS3 RNB domain, suggesting their impact on the exonucleolytic activity of the enzyme [(4); Figure 1A]. In comparison, no cancer-associated mutations have been found in the PIN domain, entirely responsible for the endoribonucleolytic activity of hDIS3 (Figure 1A).

To analyze the impact of *hDIS3* mutations identified in MM patients by Chapman *et al.* (4) on the exonucleolytic activity of the protein, recombinant versions of hDIS3 [wild-type (WT) or bearing different mutations detected in MM] were purified from *E. coli*. We successfully obtained hDIS3^S477R^, hDIS3^V504G^, hDIS3^G766R^, hDIS3^R780K^ and hDIS3^I845V^ proteins (Figure 1B), but were unable to purify hDIS3^A524P^, which was insoluble irrespective of expression conditions. In parallel, we purified two control hDIS3 variants: hDIS3^WT^, which was previously shown to display robust exoribonucleolytic activity, and hDIS3^D487N^, a catalytically inactive mutant (exo^−^) with one of the aspartic acid residues coordinating magnesium in the active site of the RNB domain substituted with its amide [(26); see Supplementary Figure S6 therein, where hDIS3^D487N^ is referred to as hDIS3^RNB MUT^]. All recombinant proteins were obtained with similar efficiency and comparable degree of purity; the only significant contaminant was bacterial DnaK chaperone, which was slightly increased for hDIS3^S477R^, likely due to its lower solubility (Figure 1B).

Next, we performed a biochemical experiment using the purified hDIS3 variants and *in vitro* transcribed RNA (∼400-nt-long RNA fragment encompassing *Arabidopsis thaliana* Lsm1 open reading frame) uniformly labeled with UTP. Time-course–based analysis of RNA degradation by TLC allowed us to tentatively assess the influence of individual mutations on exoribonucleolytic activity. While the degradative potential was almost unchanged in hDIS3^V504G^ and hDIS3^I845V^ compared with hDIS3^WT^, the activity was severely reduced for hDIS3^S477R^, hDIS3^G766R^ and hDIS3^R780K^ (Figure 1C). The phenotype of hDIS3^R780K^ closely resembled that of the catalytic RNB mutant in the active site (exo^−^), hDIS3^D487N^ (Figure 1C).

**Figure 2.**
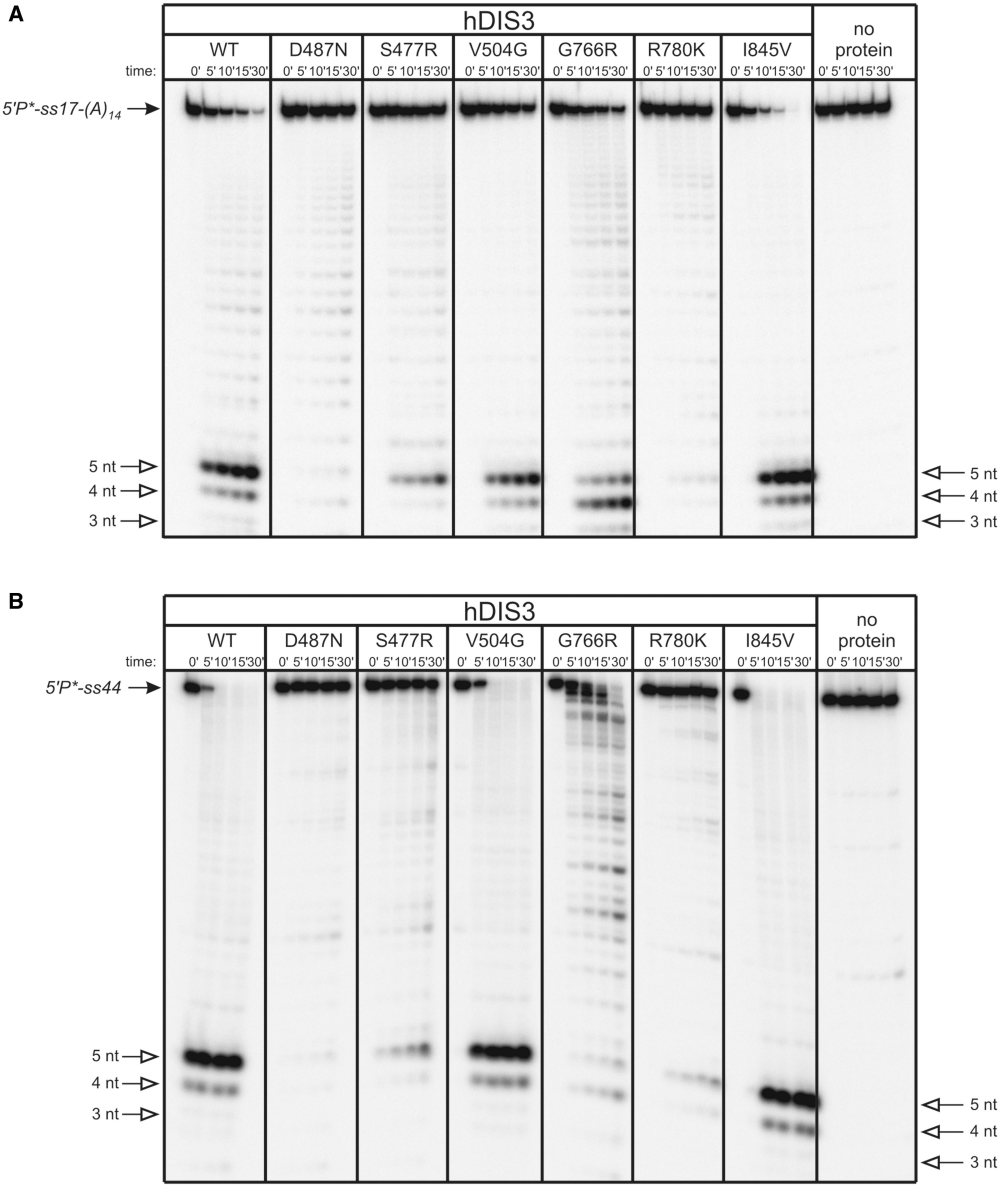

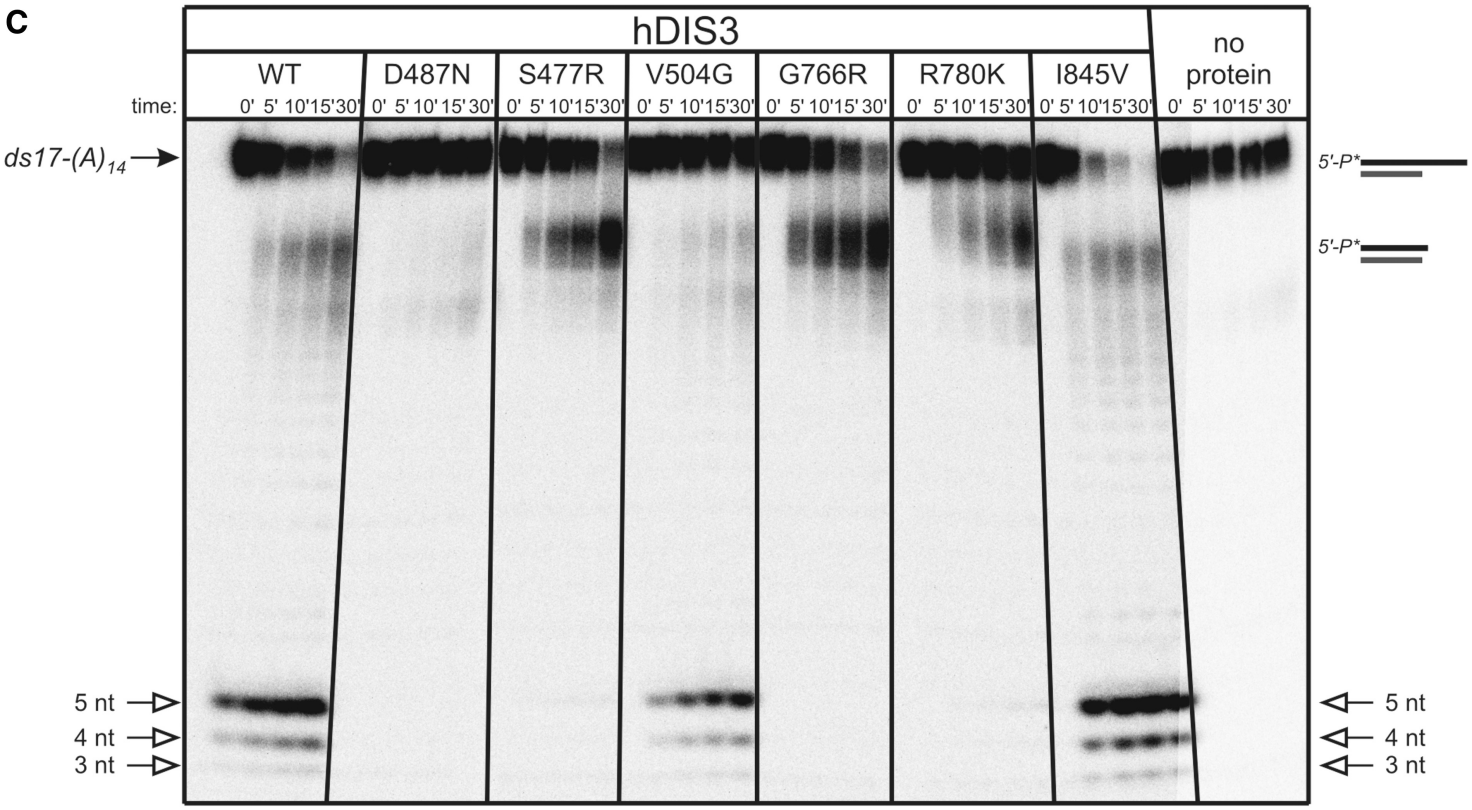
*hDIS3* mutations result in changes of the degradation pattern of unstructured RNA substrates and inability to degrade structured substrates. (**A**) 5′-labeled ss17-(A)_14_ substrate was incubated in a buffer containing 100 μM magnesium with equal amounts of hDIS3 variants or in the absence of added protein. Reactions were terminated at the indicated time points, followed by denaturing PAGE and phosphorimaging. Positions of substrate and 3–5 nt degradation products are marked with solid and open arrows, respectively. R780K mutation abolished the activity, similar to D487N, while S477R and G766R substitutions led to a change in ratio of 5-nt/4-nt products. (**B**) Experiment was performed as in (A), but using 5′-labeled ss44 oligonucleotide. S477R and R780K mutations markedly reduced hDIS3 exoribonucleolytic activity. G766R mutation resulted in the loss of processivity. (**C**) Experiment was performed as in (A), but using 5′-labeled RNA substrate forming a partial duplex ds17-(A)_14_. hDIS3^S477R^, hDIS3^G766R^ and hDIS3^R780K^ proteins were not able to continue degradation of the substrate on encountering double-stranded region.

To more qualitatively assess defects in RNA degradation caused by *hDIS3* mutations, we performed assays on various 5′-end labeled oligoribonucleotides: (i) a single-stranded RNA, composed of 17-mer generic sequence, followed by 14 nt-long oligo(A) tail [5′P*-ss17-(A)_14_]; (ii) a 44-mer, lacking oligo(A) extension (5′P*-ss44) and (iii) a structured substrate containing a 17-nt duplex followed by a 14-nt-long oligo(A) tail [5′P*-ds17-(A)_14_]. Decay products were analyzed on denaturing polyacrylamide gels (Figure 2). In the hDIS3^I845V^ and hDIS3^V504G^ mutants, patterns of final degradation products were the same as for hDIS3^WT^ for all substrates (Figure 2A–C). The rate of degradation was, however, slightly slower for the hDIS3^V504G^ variant compared with hDIS3^WT^ and hDIS3^I845V^. In contrast, the remaining mutations had a visible impact on hDIS3 function. R780K led to virtually complete inhibition of exonucleolytic degradation of both single-stranded substrates, similar to the catalytic D487N mutation (Figure 2A and B), which confirmed the TLC results shown in the Figure 1B. hDIS3^S477R^ and hDIS3^G766R^ variants displayed somewhat milder defects on the 5′P*-ss17-(A)_14_, in regard to changes in the ratio of final degradation products compared with hDIS3^WT^: for the former variant, the level of 4-nt-long decay products was reduced, while with the latter, the level was increased at the expense of decreased amounts of 5-nt-long degradation products (Figure 2A). Moreover, the hDIS3^G766R^ mutant lost processivity and became distributive in the presence of 5′P*-ss44 (Figure 2B). Importantly, in the case of partially double-stranded 5′P*-ds17-(A)_14_ substrate, while hDIS3^S477R^, hDIS3^G766R^ and hDIS3^R780K^ seemed to digest a single-stranded extension, they all stalled on encountering secondary structures in the substrate (this effect was most pronounced with the hDIS3^R780K^ mutant), even though degradation of the partial duplex was not completely abolished, as was the case of hDIS3^D487N^ catalytic mutant (Figure 2C).

Together, these results strongly indicate that the S477R, G766R and R780K mutations in *hDIS3* cause significant aberrations of hDIS3 exoribonucleolytic activity, while the V504G and I845V have subtle, if any, effects.

### Mutations in yeast *DIS3* gene corresponding to those found in MM patients cause cell growth defects and molecular phenotypes strongly suggesting impaired exosome function

Since *S. cerevisiae* is the most widely used model for studying Dis3 protein function, we started our functional analyses of *hDIS3* MM mutations by constructing yeast strains expressing endogenous Dis3 with introduced equivalent amino acid changes (with the exception of hDIS3^I845V^, as this amino acid is not conserved in yeast). We successfully produced four mutants: *dis3-G833R* (corresponding to G766R in humans), *dis3-R847K* (R780K), *dis3-V568G* (V504G) and *dis3-A588P* (A524P), but not *dis3-S541R* (S477R).

**Figure 3.**
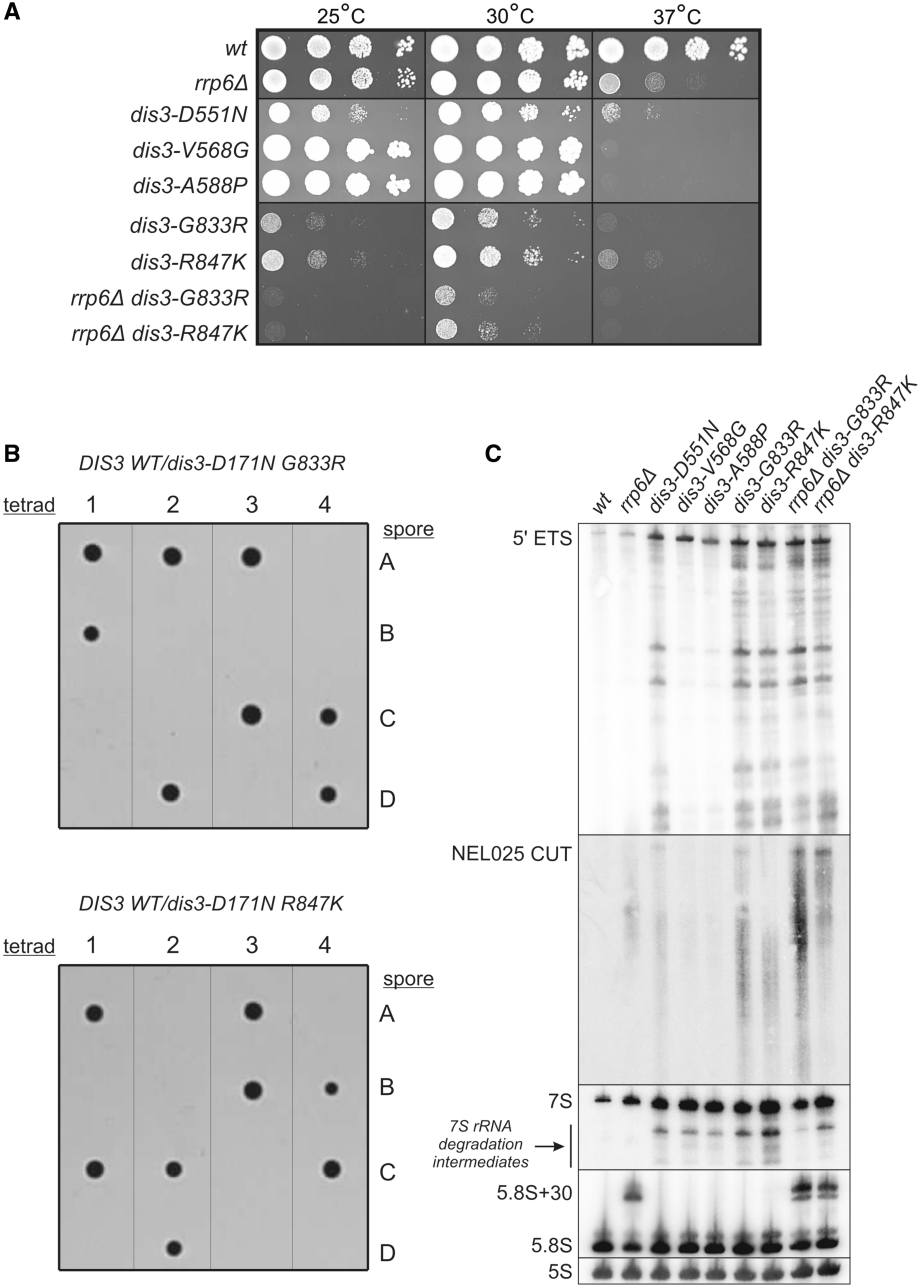
Mutations in yeast *DIS3* in positions analogous to those found in MM cause growth defects, molecular phenotypes and display synthetic genetic interaction with catalytic mutation in the Dis3 PIN domain. (**A**) Serial dilutions of indicated yeast strains were spotted on YPD plates and incubated at 25, 30 or 37°C for 60 h. V568G and A524P mutations resulted in thermosensitivity, while G833R and R847K substitutions led to growth inhibition at all tested temperatures. The two latter *DIS3* point mutations seemed to give a slight synergistic effect in *rrp6Δ* background. (**B**) Diploid heterozygotic strains *DIS3 WT*/*dis3-D171N G833R-pA* (*top*) and *DIS3 WT/dis3-D171N R847K-pA* (*bottom*), combining PIN domain endo^−^ mutation with RNB mutations analogous to those found in MM were sporulated and subjected to tetrad dissection. Only two spores from each tetrad were viable, demonstrating that MM-associated RNB domain mutations are synthetically lethal with D171N amino acid change, abolishing endonucleolytic activity of Dis3 PIN domain. (**C**) Total RNA isolated from the strains in (A) was subjected to northern blot analysis using probes specific to typical exosome substrates. *DIS3* mutations (G833R and R847K in particular) caused accumulation of 5′-ETS, NEL025 CUT and 7S precursor of 5.8S rRNA synthesis (as well as their degradation intermediates).

We first performed growth tests with the yeast strains. Both *dis3-G833R* and *dis3-R847K* displayed a strong growth retardation phenotype compared with the WT control, even at physiological temperature (Figure 3A). The growth defect of *dis3-G833R* mutant was even stronger than that observed for the *dis3-D551N* (D487N in humans) catalytic mutant (exo^−^). On the other hand, while the two other mutant strains, *dis3-V568G* and *dis3-A588P*, grew normally at 30°C, they appeared to be extremely thermosensitive, as their growth was virtually completely abolished at the elevated temperature (Figure 3A). The observed phenotypes were not due to the lack of expression of the *DIS3* mutants, as confirmed by western blotting against the protein A tag that was introduced at the 3′-end of all inserts during generation of the strains (Supplementary Figure S1A; see Supplementary Materials and Methods for details on strain construction). Moreover, successful purification of the other exosome subunits using protein A-tagged Dis3 variants as baits ruled out the possibility that these phenotypes may result from protein instability or inability to assemble into the complex (Supplementary Figure S1B). Therefore, the results of growth tests of yeast *DIS3* mutants provided the first *in vivo* support that the amino acids altered in MM patients might be important for exosome function. Moreover, consistent with the previous *in vitro* analyses, the V568 substitution exhibited milder defects than G833 or R847 (Figure 3A).

Previous studies of Dis3^exo^^−^ mutant, which harbors a mutation in the RNB domain that abolishes exonucleolytic activity, showed that simultaneous inactivation of either Rrp6 exonuclease or Dis3 PIN domain endonuclease activity led to synthetic lethality (15,18–20). Therefore, since G833R and R847K *DIS3* mutations resulted in the strongest growth phenotypes, we used these mutants to check for any synthetic genetic interactions with *RRP6* deletion or the D171N point mutation in Dis3 PIN domain [previously shown to abolish endonucleolytic activity of the protein, hereinafter referred to as Dis3^endo^^−^; see (18)]. Importantly both G833R and R847K *DIS3* mutations were synthetically lethal with the Dis3^endo^^−^ mutation (Figure 3B), as in the case of Dis3^exo^^−^ mutation. In contrast, only a slight synergistic growth inhibition was observed on combination of G833R and R847K *DIS3* mutations with *rrp6Δ* (Figure 3A).

We next tested the molecular effects of *DIS3* gene mutations in yeast by northern blot analyses on RNA isolated from mutant and control strains using probes for typical exosome substrates: the 5′-ETS by-product of ribosomal RNA processing, NEL025 CUT and 7S precursor in the 5.8S rRNA maturation pathway. All *DIS3* mutations (the D551N substitution in the active site and four mutations corresponding to those found in MM patients) caused comparable accumulation of full-length 5′-ETS and 7S rRNA (without affecting the levels of mature 5.8 S rRNA in the latter case) compared with the WT strain (Figure 3C). On the other hand, on examining levels of 7S and 5′-ETS degradation intermediates, as well as NEL025 CUT, we could divide *DIS3* mutations into two classes: one class that led to more significant accumulation of all above-mentioned species (D551N, G766R and R780K), and a second class with less pronounced effects (V568G and A588) (Figure 3C). The results are consistent with hDIS3 activity assays and yeast growth analysis (Figures 2 and 3A). Combining *DIS3* G766R or R780K mutation with deletion of *RRP6* did not seem to remarkably enhance the analyzed molecular phenotypes (except perhaps additional accumulation of NEL025 CUT in *rrp6Δ dis3-G766R* in comparison with *dis3-G766R* single mutant) (Figure 3C).

Altogether, experimental results from the yeast model suggest that changing amino acids in Dis3 positions analogous to those mutated in MM patients affects *S. cerevisiae* growth through the impairment of the exosome ability to degrade its physiological substrates.

### *DIS3* is essential for survival of DT40 cell line

In contrast to yeast, the cellular role of vertebrate DIS3 and its potential redundancy with other exosome-associated nucleases, RRP6 and DIS3L, has not been intensively studied. Our previous siRNA-based experiments in human cells did not reveal a strong effect of hDIS3 depletion on cell growth rate, and the molecular phenotypes regarding potential substrates such as 5.8S rRNA were relatively mild (26). Therefore, to assess the importance of the DIS3 protein in the physiology of vertebrate cells, we constructed a conditional *DIS3* knockout using DT40 chicken cells. The DT40 cell line has a high rate of homologous recombination, which allows for relatively straightforward generation of knockouts (50). Conditionality is achieved by insertion of *LoxP* sites into the integration cassettes and usage of a DT40 cell line derivative expressing tamoxifen-inducible Cre recombinase (51). We constructed vectors for chicken *DIS3* deletion that contained the human DIS3 cDNA expression cassette flanked by *LoxP* sites. Thus, after tamoxifen-regulated induction of Cre-recombinase, cells would be devoid of DIS3 activity.

**Figure 4.**
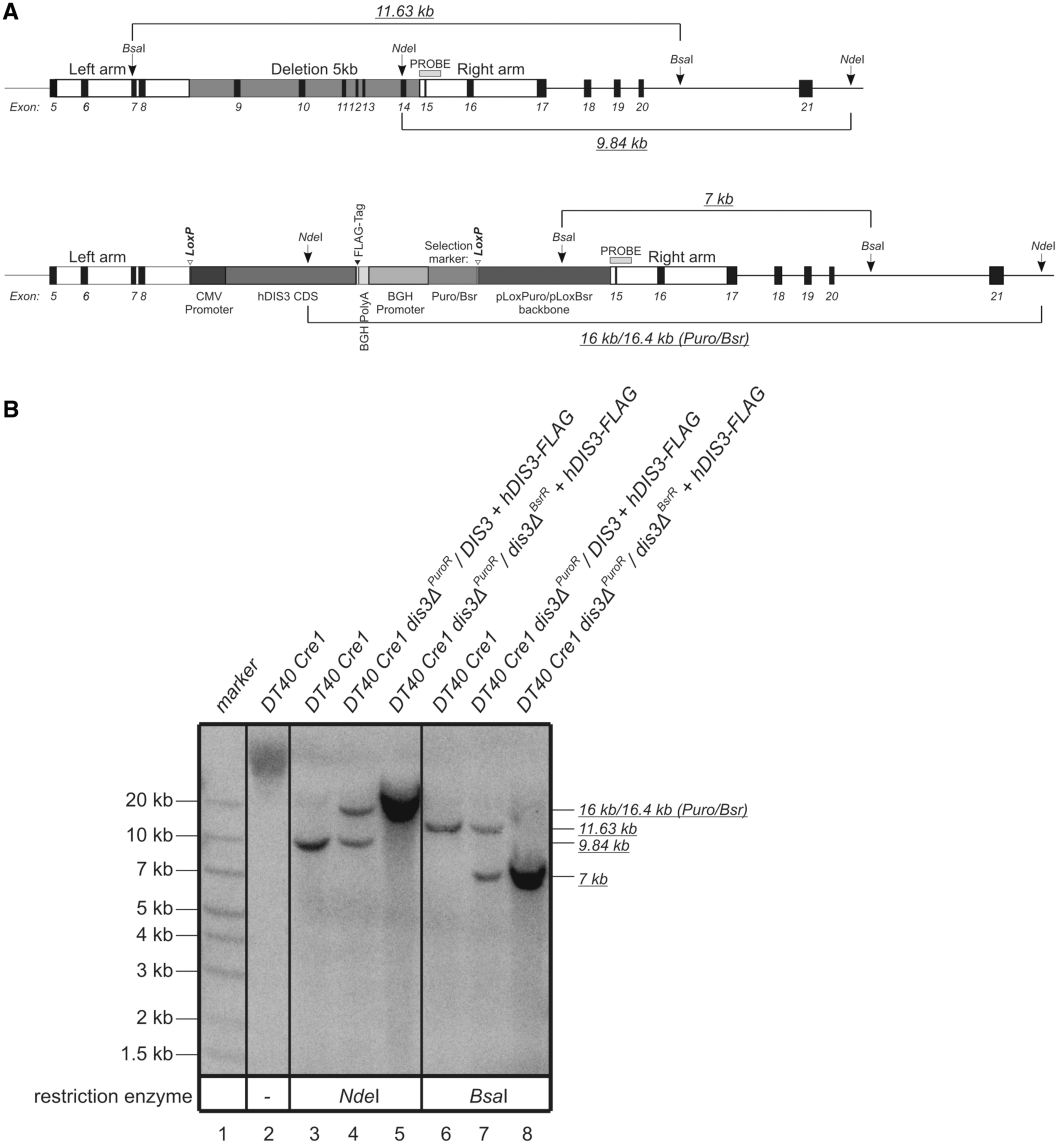
Verification of *DIS3* deletion and integration of *hDIS3* expression cassette into the genome of DT40 Cre1 chicken cell line. (**A**) Schematic representation of the fragment of *DIS3* locus in DT40 Cre1 cells before (*top*) and after (*bottom*) integration of the *hDIS3* expression cassette. Exons are numbered. Positions of the left and right arm, used in recombination, and the 5-kb fragment deleted by cassette integration (removing the majority of the chicken DIS3 RNB domain from the resulting protein), as well as elements of the integration cassette are indicated. *Nde*I and *Bsa*I sites used in Southern blot analysis are marked with arrows. Open arrowheads indicate *LoxP* sites, which were used for tamoxifen-induced Cre-mediated excision of the *hDIS3* expression cassette. Horizontal light-gray bar shows location of the probe used in Southern hybridization. Lengths of the DNA fragments generated by restriction enzymes are indicated by italicized underlined numbers. Note that the size of the *Nde*I-derived fragment differs in the case of the locus following integration, depending on the selection marker present (Puro or Bsr). (**B**) Results of Southern hybridization. Total genomic DNA was isolated from the parental DT40 Cre1 cell line (lanes 2, 3, 6), heterozygotic *DIS3* knockout with Puro selection marker (lanes 4, 7) or homozygotic *DIS3* knockout with both Puro and Bsr selection markers (lanes 5, 8). DNA was either nondigested (lane 2) or digested with *NdeI* (lanes 3–5) or *BsaI* (lanes 6–8), transferred onto membrane and hybridized with a PCR-based probe as marked in (A). Sizes of the molecular marker (GeneRuler^TM^ 1 kb Plus DNA Ladder from Fermentas; lane 1) are indicated on the left. Lengths of the restriction fragments recognized by the probe (indicated on the right) are as expected.

Two rounds of successful integrations into the DT40 genome resulted in construction of a cell line in which a large part of the endogenous *DIS3* gene was deleted (Figure 4A) and hDIS3 was expressed, as verified by Southern hybridization and western blotting, respectively (Figure 4B and Supplementary Figure S1C). Modified heterozygotic and homozygotic *DIS3-*knockout cells were treated with 4-hydroxy-tamoxifen to excise the cassettes expressing hDIS3, and then subjected to subcloning. Despite numerous attempts, it was not possible to obtain clones with excised cassettes from the *DIS3* homozygotic knockout line, while these clones were successfully obtained from the heterozygotic cell line. These results suggest that the *DIS3* gene is essential for survival of the DT40 cell line.

### MM-associated *hDIS3* mutations result in accumulation of different RNA species in the human cellular model

The promising results of the biochemical analyses and experiments in yeast and DT40 cells prompted us to test the impact of *hDIS3* mutations in humans using a proper cellular model. The most appropriate approach would be to identify MM cell lines bearing different *hDIS3* variants analyzed in previous experiments. However, sequence analysis of several commercially available MM cell lines did not reveal the presence of mutations in *hDIS3* (Supplementary Figure S2A). Most surprisingly, we were unable to confirm the sequence change detected by Chapman *et al.*(4) in H929 established cell lines from two different sources (Supplementary Figure S2A and B). Although the I845V mutation was identified in the SKMM1 cell line (Supplementary Figure S2A and C), we opted against using this line since the mutation does not influence the exoribonucleolytic activity of the enzyme (Figures 1C and 2). Therefore, we constructed our own cellular model that would allow us to analyze the influence of *hDIS3* mutations on cell physiology and RNA metabolism. We combined the Flp-In^TM^ T-REx^TM^ system from Invitrogen with a compatible vector containing a bidirectional tetracycline-inducible promoter (52) (Supplementary Figure S3; see Supplementary Materials and Methods and Supplementary Figures S4 and S5 for details on cloning strategy and construction of cell lines). This experimental setup allowed us to generate HEK293 Flp-In T-REx cell lines stably co-expressing (i) sh-microRNA that silenced the endogenous *hDIS3* copy and (ii) an exogenous hDIS3 variant tagged with a FLAG epitope and containing a recoded fragment to render it insusceptible to miRNA silencing (Supplementary Figure S3). We established four cell lines exogenously expressing hDIS3^WT^, the catalytic mutant hDIS3^D487N^ or one of the two MM-associated hDIS3 variants that had produced the strongest phenotypes in previous experiments: hDIS3^G766R^ and hDIS3^R780K^. Northern and western blot analyses confirmed that both inserts were efficiently expressed in all four cell lines (Supplementary Figure S6A and B). Importantly, exogenous proteins were completely absent from cells not subjected to induction, similarly to nontransfected HEK293 Flp-In T-Rex cells, indicating that the system that we used was relatively tight (Supplementary Figure S6B). Before beginning experiments, we first verified that the expression of other known human 3′–5′ exonucleases involved in RNA metabolism, both cooperating with the exosome core, hDIS3L and hRRP6, and functioning independently of the complex (hDIS3L2), remained unchanged in our experimental system (Supplementary Figure S6C). Furthermore, immunolocalization experiments confirmed that neither overexpression of hDIS3 nor the introduced mutations influenced the intracellular localization of the protein (Supplementary Figure S7A and B).

Using our model cell lines, we examined whether expression of mutant hDIS3 proteins led to aberrations in metabolism of RNA molecules representing various classes. We isolated total RNA from the cells, which were either untreated or subjected to doxycycline-mediated induction for 48 h. Notably, after induction, cells expressing hDIS3^D487N^ and hDIS3^R780K^ mutants grew at a slightly lower rate than those producing hDIS3^WT^ or hDIS3^G766R^ variants (data not shown).

**Figure 5.**
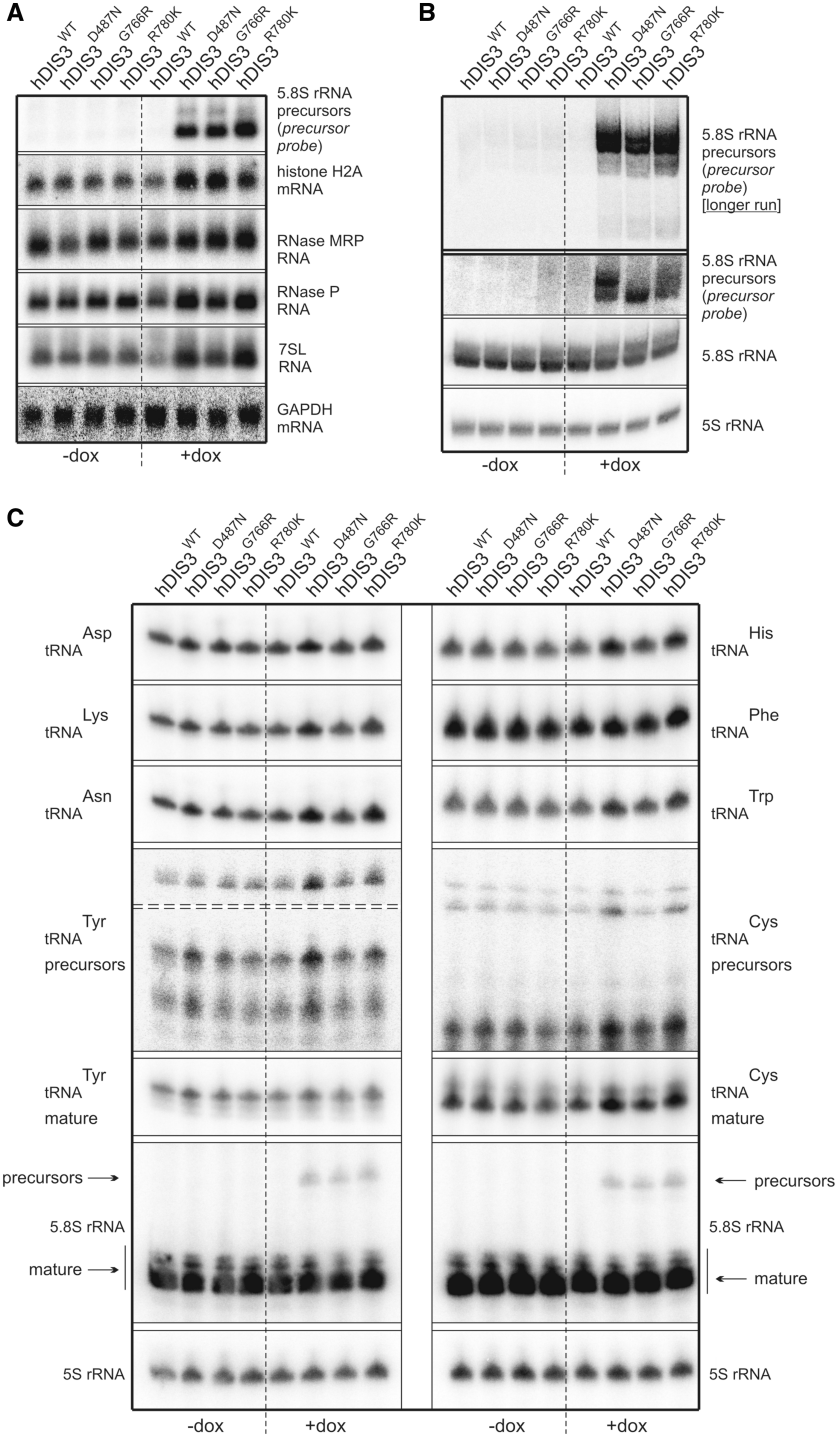

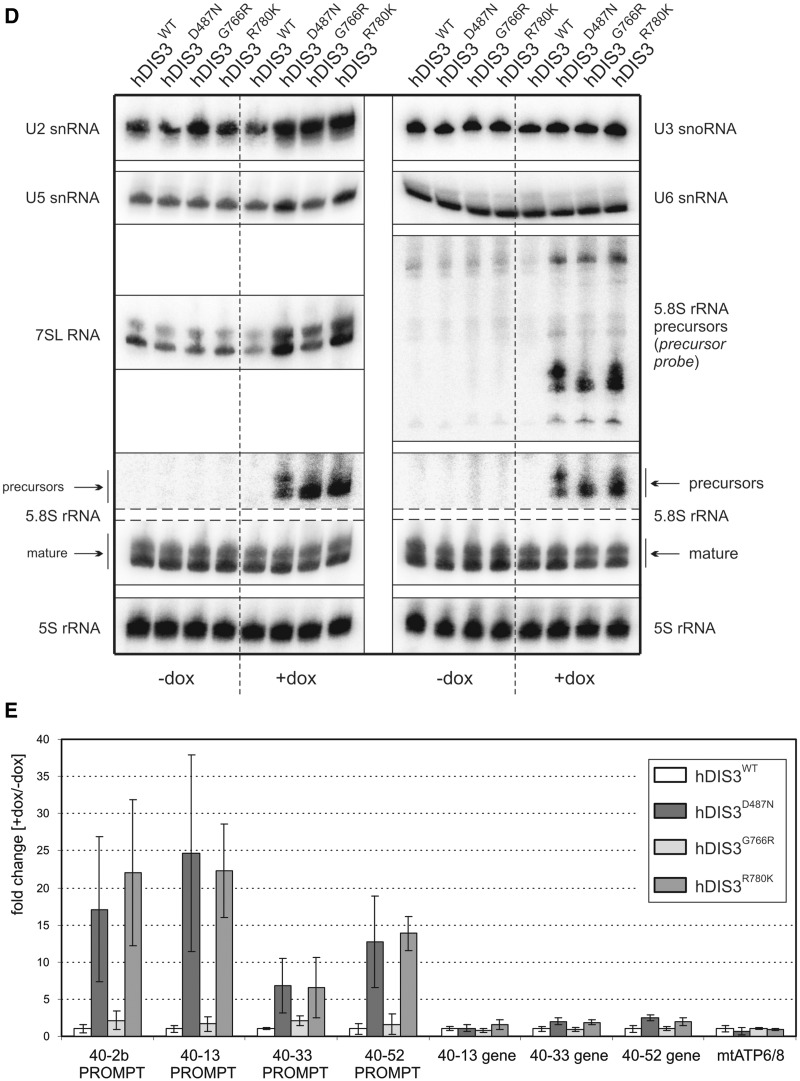
Model cell lines producing hDIS3 variants accumulate transcripts representing various RNA classes. (**A**) Low-resolution northern blot analysis of steady-state levels of RNA molecules synthesized by different RNA polymerases. Total RNA was isolated from cell lines expressing either WT or mutated hDIS3, either untreated (‘−dox’) or treated with doxycycline (‘+dox’). Following electrophoretic separation in a denaturing agarose gel, RNA was transferred onto a membrane, which was then sequentially hybridized with probes recognizing RNA pol I (5.8S rRNA precursor), RNA pol II (histone H2A mRNA, GAPDH mRNA) or RNA pol III transcripts (RNase P RNA, RNase MRP RNA, 7SL RNA), and signals were visualized by phosphorimaging. A significant increase in levels of 5.8S rRNA 3′-extented precursors was visible on induction, while a more modest accumulation was apparent for the histone H2A transcript, as well as RNase P and 7SL RNAs. For quantification of the results, see Supplementary Figure S8A. (**B**) High-resolution northern blot analysis of 5.8S rRNA and its precursor. The same RNA samples as in (A) were resolved in denaturing polyacrylamide gels for 1 h or for 2 h (‘longer run’; *upper section*). Following transfer, membranes were hybridized with probes for 5.8S rRNA or the region downstream its 3′ border (precursor probe); 5S rRNA was used as a loading control. Accumulation of slightly different precursors was observable depending on the mutant hDIS3 variant, without concomitant decrease of mature 5.8S rRNA levels. (**C**) High-resolution northern blot analysis of tRNAs. RNA samples as in (A) were separated in denaturing polyacrylamide gels and analyzed as above using probes for various tRNAs. Induction-dependent accumulation was observed for the majority of analyzed tRNAs and precursors of tRNA^Tyr^ and tRNA^Cys^, particularly in cells producing hDIS3^D487N^ and hDIS3^R780K^ proteins. Hybridization with the probe for 5.8S rRNA served as positive control. For quantification of the results, see Supplementary Figure S8B. (**D**) High-resolution northern blot analysis of selected sn/snoRNAs. RNA samples as in (A) were analyzed as above, using probes specific to three different snRNA and U3 snoRNA. Induction-dependent accumulation was noticeable in the case of U5 snRNA; hybridizations with probes for 7SL RNA and 5.8S rRNA served as positive controls. For quantification of the results, see Supplementary Figure S8C. (**E**) Quantitative PCR analysis of different PROMPT regions and corresponding protein-coding transcripts in cell lines bearing various hDIS3 mutations. The graph shows results of quantification of three independent experiments; values on the left represent fold increase of transcripts in cells treated with doxycycline versus untreated cells. GAPDH mRNA, which was found to be relatively insensitive to hDIS3 mutations in northern blots [see panel (A)], was used for normalization. Abundance of all analyzed PROMPTs was significantly higher in cells producing D487N and R780K hDIS3 variants compared with the WT control. This effect was not seen for mRNAs synthesized under the control of respective promoters located downstream of three of the analyzed PROMPT regions or for the unrelated mitochondrial transcript, ATP6/8, which was used as a negative control.

First, we performed low-resolution northern blot analysis for selected transcripts synthesized by different RNA polymerases. Above all, we noticed significant accumulation of unprocessed 5.8S ribosomal RNA precursors (RNA polymerase I transcripts) following doxycycline-mediated induction in cells bearing mutated *hDIS3* variants (but not the WT counterpart) (Figure 5A). In the case of RNA polymerase II transcripts, while GAPDH polyA^+^ mRNA levels, used for normalization, remained constant in all cell lines irrespective of induction, we observed a clear increase of histone H2A polyA^−^ mRNA amounts in cells expressing hDIS3^D487N^, hDIS3^G766R^ and hDIS3^R780K^ mutant proteins (Figure 5A; see Supplementary Figure S8A for quantification). In regard to RNA polymerase III transcripts, although expression of *hDIS3* mutants did not seem to have significant impact on RNase MRP RNA (Figure 5A and Supplementary Figure S8A), the levels of two other representative RNA species synthesized by this enzyme, namely RNase P RNA and 7SL RNA, were increased, particularly in cells producing hDIS3^D487N^ and hDIS3^R780K^ variants (Figure 5A and Supplementary Figure S8A). The phenotype observed for 7SL RNA was also confirmed by high-resolution northern blot analysis (Figure 5D).

As the degree of accumulation was the strongest for 5.8S rRNA precursors, we analyzed this in more detail by high-resolution northern blots, using 5S rRNA as loading control (Figure 5B). In general, we confirmed that the levels of these species were elevated exclusively on induction in cells with *hDIS3* mutations (Figure 5B). Additionally, we found that (i) the patterns of observed precursors differed among the individual mutants and (ii) the patterns of accumulating precursors were more similar between *hDIS3* D487N and R780K mutants than those in cells expressing hDIS3^G766R^ (Figure 5B). Furthermore, despite the significant processing defects, levels of mature 5.8S rRNA were not markedly reduced (Figure 5B). Consequently, neither ribosome biogenesis nor polysome formation seemed to be significantly disturbed (Supplementary Figure S9A). Interestingly, the observed 5.8S rRNA precursors were incorporated into polysomes (Supplementary Figure S9B). It is also worth noting that their accumulation was not further increased by siRNA-mediated silencing of *hRRP6* expression (Supplementary Figure S10).

Two recent publications underscored the importance of exosome and catalytic Dis3 activities in particular, in the global degradation of noncoding RNAs in yeast, and accumulation of tRNAs and their precursors was reported as one of the most prominent phenotypes resulting from Dis3 inactivation (37,53). These results, together with the increased levels of other RNA polymerase III transcripts in cells producing mutant hDIS3 (Figure 5A and Supplementary Figure S8A), prompted us to analyze the influence of *hDIS3* mutations on selected tRNA molecules more carefully. We noted that almost all examined mature tRNA (except tRNA^Tyr^ and tRNA^Phe^), as well as precursors of tRNA^Tyr^ and tRNA^Cys^, accumulate in cells bearing exogenous *hDIS3* with D487N or R780K mutations following doxycycline treatment (Figure 5C; for quantification see Supplementary Figure S8B). Although the effect was not as pronounced as for 5.8S rRNA precursors (Figure 5A–D), it was reproducible. It is again worth emphasizing that amino acid substitutions at positions 487 and 780 exerted stronger molecular phenotypes than the G766R change (Figure 5C and Supplementary Figure S8B).

We also investigated the impact of *hDIS3* mutations on the accumulation of U3 snoRNA and three different snRNAs; however, we only noticed a phenotype for U5 snRNA that resembled the previously observed phenotype with tRNAs (Figure 5D; for quantification see Supplementary Figure S8C). We also analyzed PROMPTs, which are unstable transcripts previously shown to accumulate in cells subjected to siRNA-mediated depletion of human exosome subunits (26,39,40). We found that the levels of all analyzed PROMPTs were increased in all cell lines producing mutated hDIS3 variants (Figure 5E). In agreement with other analyses, accumulation of PROMPTs was far more pronounced in cells bearing *hDIS3* D487N or R780K mutations than in the case of G766R substitution (Figure 5E). Additionally, we analyzed corresponding protein-coding transcripts synthesized from promoters localized downstream of three out of four PROMPTs and found that they were not affected by *hDIS3* mutations, similar to the unrelated mitochondrially encoded transcript, ATP6/8 (Figure 5E).

In summary, *hDIS3* mutations in our model cell lines resulted in accumulation of the majority of analyzed exosome substrates, including 5.8S processing intermediates, tRNAs, RNA polymerase III transcripts and PROMPTs. Although the degree of accumulation was variable among different exosome targets, in most cases we observed more significant effects in cells producing the hDIS3^D487N^ and hDIS3^R780K^ than the hDIS3^G766R^ variant.

### MM-associated *hDIS3* mutations lead to growth inhibition of human cells and are synthetically lethal with PIN domain catalytic mutations

**Figure 6.**
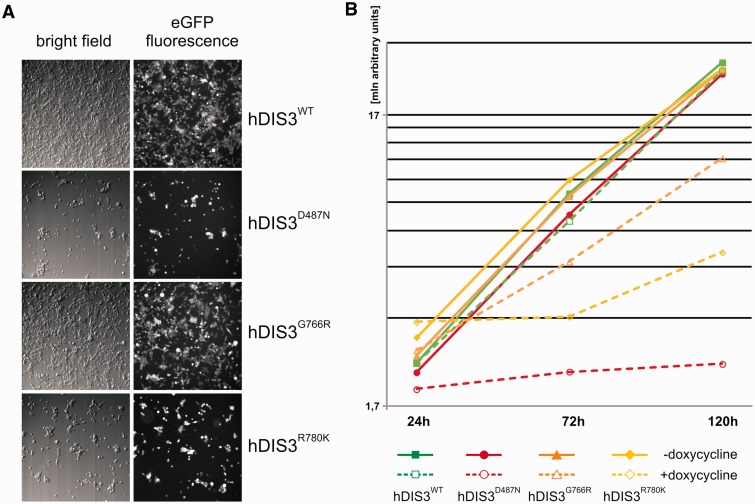
MM-associated *hDIS3* mutations adversely affect cell growth and metabolic activity in a human cellular model. (**A**) Cell-growth analysis. Equal amounts of cells from each line were seeded in culture dishes, subjected to doxycycline-mediated induction (48 h + 48 h) and analyzed by microscopy. The cells harboring mutated hDIS3 grew worse than the cell line with WT protein. The strength of the growth phenotype was as follows: hDIS3^D487N ^> hDIS3^R780K ^> hDIS3^G766R^. (**B**) Metabolic activity assay. Approximately equal numbers of cells from each stable cell line were grown in triplicate in 96-well plates, either untreated (‘−dox’) or treated with doxycycline (‘+dox’). AlamarBlue® reagent was added to the cells after the indicated time, and changes of metabolic status were evaluated by fluorescence measurements. In agreement with results from (A), hDIS3^D487N^ and hDIS3^G766R^ mutants gave the strongest and weakest phenotype, respectively.

We noticed that cells expressing hDIS3 variants with D487N or R780K substitutions proliferated at somewhat slower rate following the doxycycline-mediated induction compared with the two other cell lines. Therefore, we performed additional growth and metabolic analyses by observing the untreated cells and their counterparts over a long induction period, 48 h of culture, followed by passage of cells into fresh medium containing doxycycline and another 48 h of culture. While all cell lines grew normally and comparably in medium lacking doxycycline, on longer sh-miRNA–mediated repression of endogenous *hDIS3* and simultaneous expression of sh-miRNA-insensitive exogenous hDIS3 variants, the four cell lines behaved differently (Figure 6). hDIS3^WT^ produced from the integrated construct complemented the silenced endogenous *hDIS3*, and the cell growth and metabolism were undisturbed (Figure 6A and B). In contrast, cells expressing hDIS3^D487N^ and hDIS3^R780K^ variants exhibited inhibition of growth and reduction of metabolic activity (Figure 6A and B), while G766R mutation in *hDIS3* exerted only moderate effects (Figure 6A and B). These results were in line with biochemical and functional data (see Figures 1C, 2 and 5).

**Figure 7.**
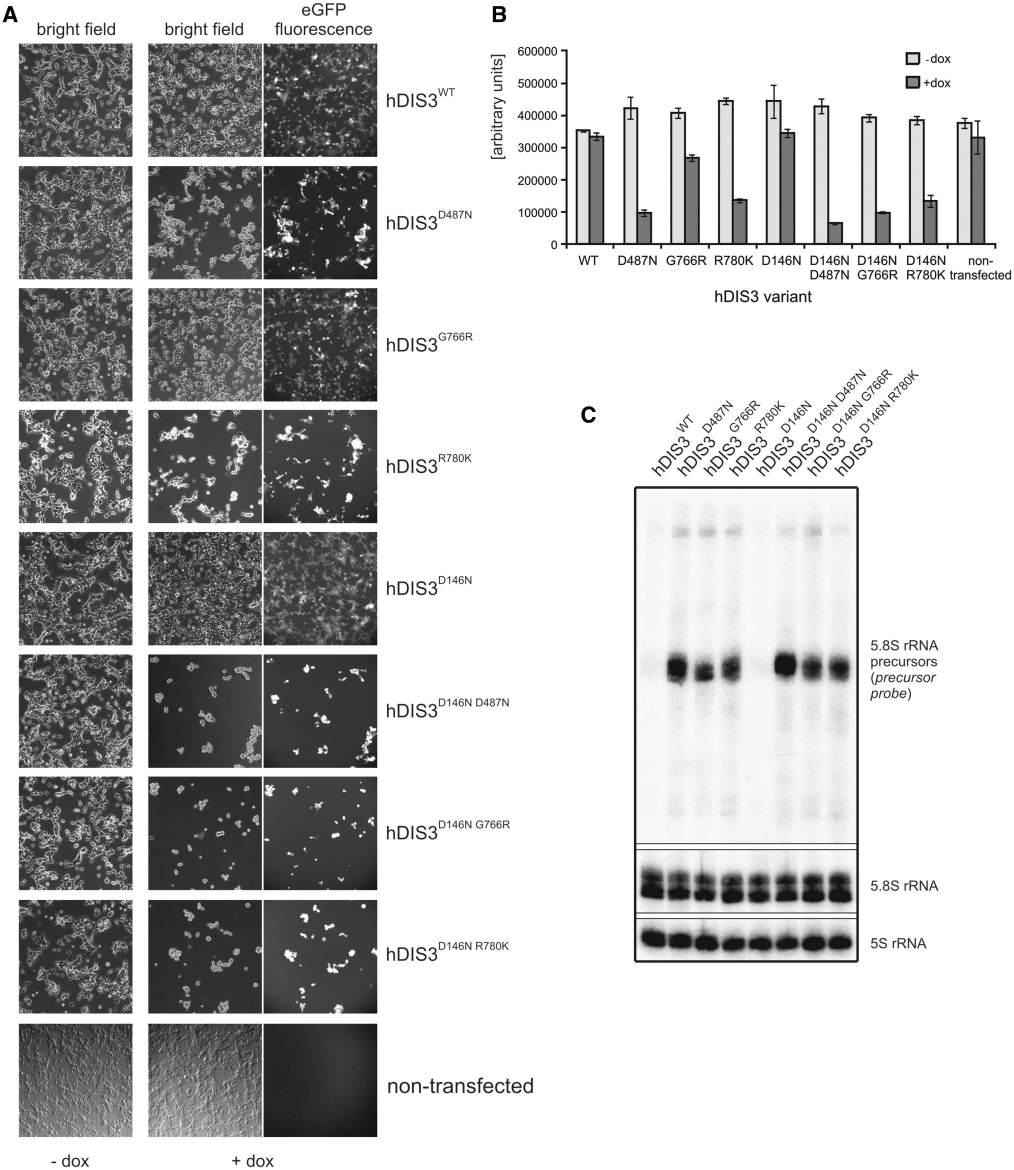

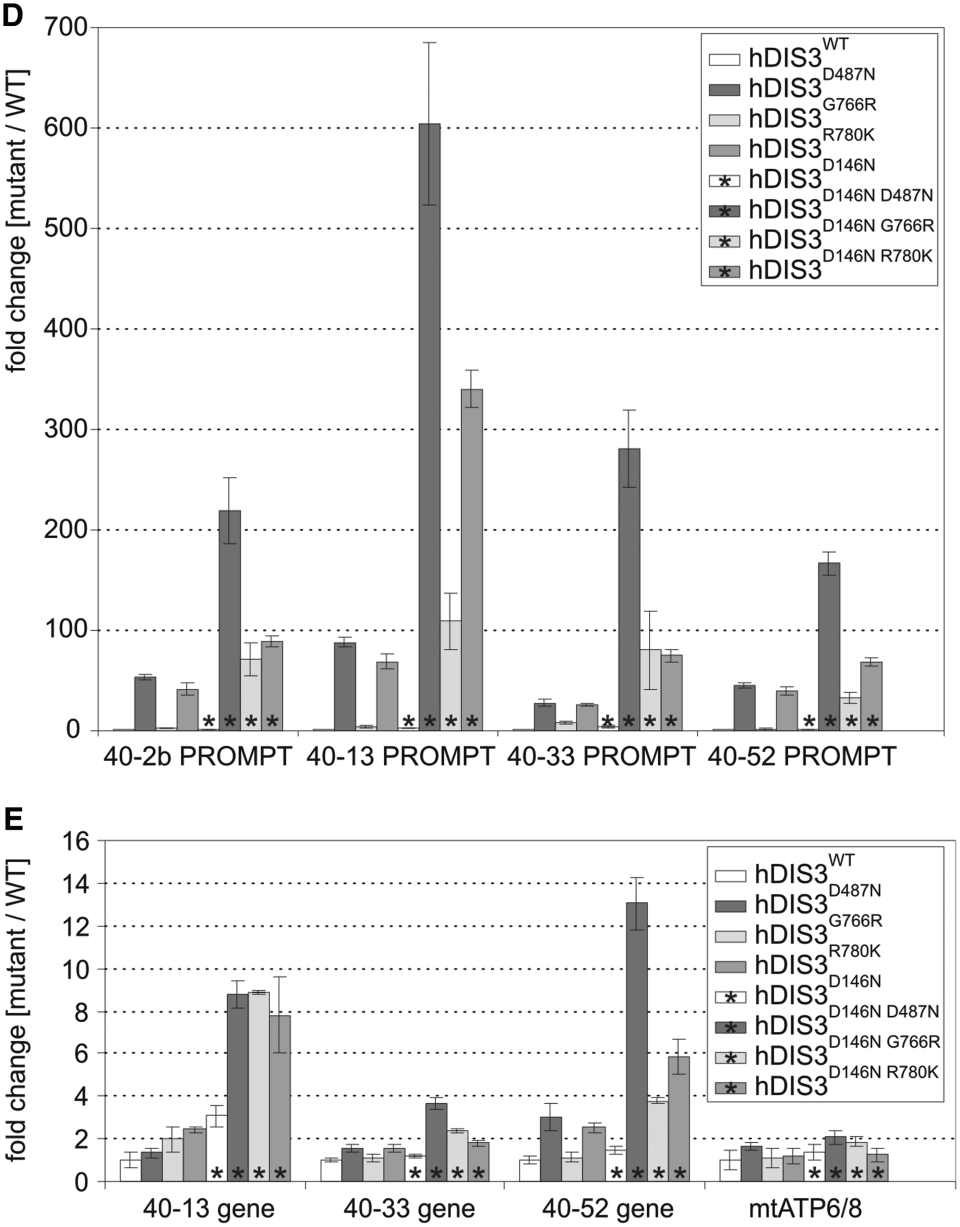
Catalytic mutation in the hDIS3 PIN domain, responsible for endonucleolytic activity, leads to synergistic effects with MM-associated mutations in the RNB domain. (**A**) Cell-growth analysis. Equal amounts of cells from each line were seeded in culture dishes, grown in the absence (‘−dox’) or presence (‘+dox’) of doxycycline and analyzed by microscopy. The cell lines producing hDIS3 with mutations in both catalytic domains grew significantly worse than cells producing hDIS3 with mutations only in the RNB domain. Nontransfected HEK293 Flp-In T-Rex cells were analyzed in parallel as a control. (**B**) Metabolic activity assay. Approximately equal numbers of cells from each stable cell line, or nontransfected HEK293 Flp-In T-Rex cells, were grown in triplicate in 96-well plates, either untreated (‘−dox’) or treated with doxycycline (‘+dox’). AlamarBlue® reagent was added after 72 h, and metabolic status was assessed by fluorescence measurements. In agreement with results from (A), cells producing hDIS3 double mutants displayed lower metabolic activity than cells expressing protein variants with an intact PIN domain. (**C**) Catalytic mutation of PIN domain does not enhance accumulation of 3′-extended 5.8S rRNA precursor molecules. High-resolution northern blot analysis of 5.8S rRNA and its precursor. Total RNA was isolated from cell lines producing WT hDIS3 or hDIS3 PIN or/and RNB domain mutants, which were all subjected to doxycycline treatment. RNA samples were resolved in denaturing polyacrylamide gel for 2 h; following RNA transfer, nylon membranes were hybridized with probes for mature 5.8S rRNA or to the region located downstream its 3′ border (precursor probe); 5S rRNA was used as a loading control. (**D** and **E**) Quantitative PCR analysis of different PROMPT regions (D) and corresponding protein-coding transcripts (E) in cell lines producing hDIS3 variants. The graphs show results of quantification of three independent experiments; values on the left represent fold increase of transcripts in cells expressing mutated versus WT hDIS3. GAPDH mRNA was used for normalization. Abundance of all analyzed PROMPTs was highly increased in cells producing hDIS3 double mutants compared with cells expressing proteins harboring mutations only in the RNB domain (D); the same trend was also observed for mRNAs synthesized under the control of respective promoters located downstream of three of the analyzed PROMPT regions, but not for the unrelated mitochondrial transcript, ATP6/8, which was used as a negative control (E).

Next, we were interested in checking whether, as in yeast, there is a synergistic effect of MM-associated RNB domain mutations and dysfunction of hDIS3 PIN domain endonuclease activity. We constructed analogous stable cell lines bearing an additional mutation in the catalytic site of PIN domain, D146N, which was previously shown to abolish hDIS3 endonucleolytic activity in the *in vitro* assays (26). Cell growth assays performed as above revealed that the growth of cell lines expressing hDIS3 variants with the D146N mutation was unaffected in the absence of doxycycline (Figure 7A). On the other hand, on induction, only cells expressing the hDIS3^D146N^ single mutant grew relatively normally and comparably with its counterpart producing hDIS3^WT^, indicating that endonuclease activity alone has little impact on cell physiology (Figure 7A). In contrast, combination of the PIN domain catalytic mutation with any mutation in the RNB domain (D487N, G766R or R780K) strongly suppressed survival of cells (Figure 7A). A synergistic effect of inactivation of both nucleolytic activities of hDIS3 was most pronounced in cells expressing the hDIS3^D146N G766R^ double mutant, particularly when noting that the cell line producing the hDIS3^G766R^ mutant alone displayed mild growth inhibition compared with the WT control (Figure 7A). Importantly, the metabolic activity of the cells bearing mutations in the RNB domain was also additionally reduced on accompanying inactivation of PIN domain endonuclease (Figure 7B).

We then analyzed molecular phenotypes resulting from combination of MM-associated *hDIS3* mutations in the RNB domain with disruption of PIN domain endonucleolytic activity by comparing them with the respective single mutants. As shown in Figure 7C, inactivation of the PIN domain catalytic activity alone did not lead to accumulation of 5.8S rRNA precursors. Interestingly, we were not able to observe any synergistic effect of D146N substitution in the background of RNB domain mutations, as the levels of precursor molecules were not further elevated in cells expressing hDIS3^D146N D487N^, hDIS3^D146N G766R^ and hDIS3^D146N R780K^ compared with the cells producing respective hDIS3 versions with an intact PIN domain (Figure 7C). This indicates that such 3′-extended 5.8S rRNA species are normally degraded mainly by hDIS3 exoribonucleolytic activity. On the contrary, our results clearly demonstrated that while the levels of different PROMPTs were comparable between cell lines producing either hDIS3 with D146N substitution or its WT counterpart, the cells expressing hDIS3^D146N D487N^, hDIS3^D146N G766R^ and hDIS3^D146N R780K^ double mutants accumulated much higher amounts of PROMPTs than the respective single mutants (Figure 7D), suggesting the cooperative action of both nucleolytic activities of hDIS3. Intriguingly, we noticed a highly similar trend for protein-coding transcripts corresponding to three out of four tested PROMPTs (but not for mitochondrially encoded ATP6/8 mRNA, which we would not expect to be affected by exosome dysfunction), although the fold differences between double and single mutants were not so prominent as in the case of PROMPTs (Figure 7E).

In conclusion, although the molecular events linking the inactivation of hDIS3 nucleolytic properties and its inability to efficiently degrade transcripts with reduced cell viability remain to be elucidated, the negative synthetic interaction between MM-associated RNB domain mutations and PIN domain dysfunction may suggest the latter as a promising drug target for cancers bearing mutations that affect hDIS3 RNB domain exonucleolytic activity.

## DISCUSSION

In this study, we showed that MM-associated *hDIS3* mutations have negative effects on hDIS3 protein function, cell physiology and RNA metabolism, leading to vulnerabilities that can be used for anti-cancer drug development. Moreover, our analyses prove that DIS3 has an essential function in RNA metabolism in vertebrates.

### Inhibition of hDIS3 exonucleolytic activity by MM-associated mutations

Results of biochemical assays indicate that in the majority of cases, amino acids substituted in hDIS3 in MM patients play an important role in regulating hDIS3 exoribonucleolytic activity, which is generally in agreement with data from literature and structural information. Five out of six analyzed mutations are positioned in the RNB domain, strictly associated with exoribonucleolytic activity (Figure 1A; Supplementary Figure S11). The only exception is isoleucine at position 845 (I845), which is located in a less conserved region downstream of the RNB domain; its occurrence is limited exclusively to the human protein (Figure 1A; Supplementary Figure S11). Accordingly, activity of hDIS3^I845V^ was unaffected, irrespective of whether tested on single- or partially double-stranded RNA substrates.

Although our results do not allow us to directly propose the possible mechanism of inhibition of hDIS3 exoribonucleolytic activity resulting from MM-associated mutations, some clues can be obtained following comparison of the solved 3D structures of *E. coli* RNase II and yeast Dis3 RNB domains {the latter in the context of exosome core, according to the recently published structure of 11-subunit exosome [(25); Supplementary Figure S12A]}. For instance, S541 in *S. cerevisiae* Dis3 (an equivalent of S477 in hDIS3) and threonine in the corresponding position of *E. coli* RNase II (Supplementary Figure S11) are located in the proximity of RNB domain catalytic center (Supplementary Figure S12B). We may hypothesize that substitution with large amino acid, like arginine (as in the case of the S477R *hDIS3* mutation), may affect corresponding β-sheet formation and most likely result in a steric conflict and/or abolish hydrogen bonds required for correct structure, therefore interfering with magnesium coordination and decreasing enzymatic activity. On the other hand, perfectly conserved G833 in yeast Dis3 (a counterpart of G766 in hDIS3) is situated in the ‘neck region’ conserved between RNase II and Dis3, which is responsible for binding of the 3′-end of RNA (Supplementary Figure S12C). A potentially important function of glycine in this particular position is further underscored by the fact that the G833D mutation is known to suppress degradation of hypomodified 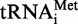 in *S. cerevisiae* (43,54). Introduction of a negatively charged amino acid might disrupt the interaction between protein and RNA. Substitution of glycine with a bigger amino acid, such as arginine (as in the case of the human G766R mutation), although positively charged, may result in a steric hindrance. Additionally, this particular glycine is located at the top of a loop, and flexibility of this small amino acid may be crucial for the structural integrity. In turn, R780/R847 in human/yeast Dis3 is in the same position as R500 in *E.coli* RNase II; this amino acid is localized in the active site of the bacterial enzyme [(55); Supplementary Figure S12D] and its substitution with alanine leads to an over 40 000-fold reduction of the exoribonucleolytic activity (56). We may thus infer that, similar to *E. coli* RNase II, the role of arginine in this specific location is to increase susceptibility of the phosphodiester bonds present in RNA substrates to cleavage. All the findings mentioned above nicely corroborate our results demonstrating that the exoribonucleolytic activity of hDIS3 is affected most significantly in protein variants bearing S477R, G766R or R780K mutation.

Contrary to the mutations mentioned above, V504G substitution did not have any impact on hDIS3 exoribonucleolytic activity. It might be a bit surprising, as valine in this position is perfectly conserved (Supplementary Figure S11). However, superimposition of yeast Dis3 and *E. coli* RNB domain structures reveals that its counterpart in *S. cerevisiae* Dis3 (V568) is located at the periphery of the protein (Supplementary Figure S12E). Similarly, A588 in yeast Dis3 (equivalent of A524 in hDIS3) is located far from the catalytic center, in a large 4-stranded β-sheet, inside a globular hydrophobic structure (Supplementary Figure S12F). Furthermore, it is the least conserved amino acid, absent from *E. coli* RNase II/R, which both contain a basic amino acid residue in this position (K or R) (Supplementary Figure S11). Although we were not able to assess the activity of the hDIS3^A524P^ protein variant due to its insolubility, analyses performed in a yeast model indirectly indicate that the dysfunction of such mutant is not so severe as in the case of hDIS3^G766R^ or hDIS3^R780K^ versions.

Together these results show that some MM-associated *hDIS3* mutations exert strong effects on the ability of the enzyme to exonucleolytically digest RNA substrates, which is in agreement with the currently available structural information. However, it is important to point out that other *hDIS3* mutations, like I845V and V504G, do not affect the enzyme activity, what may suggest that they represent random mutational noise, since global sequencing of tumor samples leads to the discovery of thousands of single nucleotide polymorphisms and not all of them are relevant to cancer development. Nevertheless, this does not diminish the significance of other detected amino acid substitutions, as there are currently 60 described *DIS3* mutations in the COSMIC database (http://cancer.sanger.ac.uk/cancergenome/projects/cosmic) and a large fraction of them is located in the conserved region of the RNB domain.

### Importance of hDIS3 and intactness of its RNB domain for cell physiology

Results of multiple analyses performed in this study using different models highlight the importance of hDIS3 and intactness of its RNB domain for cell physiology. First, knockout of both alleles of *DIS3* gene in the chicken DT40 cell line was found to be lethal, which demonstrated for the first time that in vertebrates the gene is indispensable for survival, thus confirming findings in *S. cerevisiae*. We used this model to analyze the influence of MM-associated *DIS3* mutations in the RNB domain on cell viability and discovered that, in general, amino acid changes leading to more significantly decreased enzymatic activity of hDIS3 protein *in vitro* were reflected by more severe molecular phenotypes and growth defects of corresponding yeast mutant strains. This observation was in turn corroborated by the results of assays performed using our experimental system based on HEK293-derived human cell lines.

Among the strongest molecular phenotypes caused by MM mutations are increased levels of extended 5.8S precursor molecules, improperly processed at the 3′-end, and an intermediate in rRNA maturation pathway, 5′-ETS. In both yeast and human cells, accumulation was particularly apparent in the case of mutations most adversely affecting the activity of the protein. Intriguingly, in neither of the two models was accumulation of 5.8S rRNA precursor accompanied by concomitant significant decrease in the levels of mature 5.8S rRNA. Furthermore, we found that such precursors were successfully incorporated into polysomes in human cells, indicating that their overabundance may not influence protein synthesis. Therefore, the significance of accumulation of these species for cell physiology remains to be elucidated. Moreover, not only full-length 5.8S precursors and 5′-ETS but also their degradation intermediates accumulated in the yeast mutants, confirming previous observations that hDIS3 exoribonucleolytic activity plays a crucial role in their removal from the cell (18). It also appears that RRP6 exonuclease cannot fully take over hDIS3 functions in the degradation of such RNA species in the nucleus, as neither the deletion of *RRP6* gene in yeast nor RNA interference (RNAi)-based suppression of hRRP6 expression is human cells had an additive effect on their accumulation. However, our results do not provide explanation as to whether aberrations in ribosomal RNA maturation pathway are directly associated with cell growth defects.

Further analyses of molecular phenotypes resulting from MM-associated *hDIS3* mutations in human cells revealed that inhibition of hDIS3 exoribonucleolytic activity results in decreased degradation rate of RNA molecules belonging to other RNA classes, such as tRNAs and their precursors, snRNAs and other small RNAs, including RNase P RNA and 7SL RNA. One of the most interesting and reproducible phenotypes was the strong accumulation of PROMPTs, unstable transcripts synthesized by RNA Pol II upstream many promoters of protein-coding genes. Moreover, accumulation of PROMPTs was further enhanced when MM-associated RNB domain mutations were combined with hDIS3 D146N (endo^−^) mutation within PIN domain, indicating that degradation of these RNA species requires both exonucleolytic and endonucleolytic activities of hDIS3. It should be also pointed out that *DIS3* mutations in yeast (in which Dis3p is present both in the nucleus and the cytoplasm) lead to the accumulation of nuclear noncoding RNAs somewhat related to human PROMPTs, namely CUTs. This indicates that the level of ‘transcriptional noise’ is probably much higher in the nucleus of the cells producing mutated hDIS3 variants. Whether and how this may affect physiology, metabolic activity and survival potential of the cells remains to be addressed. Furthermore, it is essential to apply high-throughput sequencing analyses to obtain deeper insights into the transcriptome changes resulting from MM-associated *hDIS3* mutations.

Moreover, the role of *hDIS3* mutations in cancer development remains to be determined. One possibility is the involvement of hDIS3 as a part of the exosome complex, known to be involved in AID-mediated immunoglobulin hypermutations and class switches (48), which lead to DNA translocations associated with MM development. The mutations may also have specific direct effects on gene expression; alternatively, as the exosome is the main enzyme degrading unstable transcripts from noncoding regions and some such transcripts affect chromatin structure, their overabundance resulting from hDIS3 dysfunction may indirectly affect expression of genes important for carcinogenesis. All of that remains to be experimentally verified using true MM models, but since thus far there is no single established MM cell line with *hDIS3* mutation affecting activity of the protein, this is a challenging task.

### hDIS3 PIN domain as a potential drug target for cancers bearing mutations in hDIS3 RNB domain

Synthetic lethality has recently emerged as one of the most promising strategies for development of targeted cancer therapies (57). Such a strategy enables the targeting of cancer cells with a potentially low effect on untransformed cells. For example, breast cancer cells with mutations in the *BRCA1* and *BRCA2* are sensitive to inhibition of poly [ADP-ribose] polymerase (PARP1), whereas inhibition of PARP1 has little influence on survival and proliferation of normal cells with WT BRCA (58,59). Lead compounds based on PARP1 inhibitors are in advanced clinical trials (60).

In the yeast system, both deletion of accessory nuclear exosome catalytic subunit Rrp6 and inactivation of Dis3 PIN endonuclease domain are synthetically lethal with mutations abolishing Dis3 exonucleolytic activity. Since we show here that, as in yeast, vertebrate *DIS3* is essential for cell survival, it was possible that similar synthetic interactions could exist in the case of hDIS3. Results presented herein indicate that MM-associated mutations in hDIS3 RNB domain, inhibiting its exonuclease activity, are indeed synthetically lethal with inactivation of hDIS3 endonucleolytic activity. This is valid for both the yeast and cellular model described in this publication. In human cells, the combination of MM mutations with mutations in the PIN domain had a cumulative effect on cell growth inhibition and accumulation of exosome targets.

In contrast to inactivation of the DIS3 PIN domain, deletion or depletion of RRP6 was not synthetically lethal with MM-associated *hDIS3* mutations.

In summary, our data suggest the hDIS3 PIN domain but not hRRP6 as a drug target for cancers bearing *hDIS3* mutations. Importantly, mutations inactivating the hDIS3 PIN domain alone have little effect on cell physiology, since research performed by us and others did not reveal any detectable phenotype of hDIS3 PIN domain dysfunction without the presence of other mutations. This suggests that the active site of the hDIS3 PIN domain endonuclease may be a particularly promising drug target for cancers bearing mutations affecting hDIS3 RNB domain exonucleolytic activity. This is especially important for MM, as it remains incurable, although we cannot exclude the possibility that due to differences in physiology, synthetic interactions between mutations in hDIS3 PIN and RNB domains might not occur in MM cells. The DIS3 PIN domain structure is solved and the *in vitro* assays for its endoribonucleolytic activity have been developed, what is an additional advantage that should facilitate identification of its inhibitors based on classical medical chemistry or virtual screening for putative lead compounds.

## SUPPLEMENTARY DATA

Supplementary Data are available at NAR Online, including [61–65].

## FUNDING

National Science Centre [NCN Maestro: UMO-2011/02/A/NZ1/00001 to A.D.]; National Centre for Research and Development [NCBR PBS: 176911 to A.D., NCBR LIDER: LIDER/35/46/L-3/11/NCBR/2012 to R.T.]; Polish-Swiss Research Programme [PSPB-183/2010 to A.D.]; Scholarship for outstanding young scientists from the Polish Ministry of Science and Higher Education (to R.T. and R.J.S.);. Experiments were carried out with the use of CePT infrastructure financed by the European Union—the European Regional Development Fund [Innovative economy 2007–13, Agreement POIG.02.02.00-14-024/08-00]. Funding for open access charge: National Science Centre Poland [NCN Maestro: UMO-2011/02/A/NZ1/00001 to A.D.].

*Conflict of interest statement.* None declared.

## Supplementary Material

Supplementary Data
