# Supplementary material for: Multiple myeloma-associated hDIS3 mutations cause perturbations in cellular RNA metabolism and suggest hDIS3 PIN domain as a potential drug target
Source: Nucleic Acids Res. 2013 Oct 21;42(2):1270–90. doi: 10.1093/nar/gkt930 (PMC3902924; doi:10.1093/nar/gkt930)
Supplement: Supplementary Data [file supp_42_2_1270__index.html]

Multiple myeloma-associated hDIS3 mutations cause perturbations in cellular RNA metabolism and suggest hDIS3 PIN domain as a potential drug target — Multiple myeloma-associated hDIS3 mutations cause perturbations in cellular RNA metabolism and suggest hDIS3 PIN domain as a potential drug target — Supplementary Data 

# Multiple myeloma-associated *hDIS3* mutations cause perturbations in cellular RNA metabolism and suggest hDIS3 PIN domain as a potential drug target

## Supplementary Data

files

**Files in this Data Supplement:**

- Supplementary Data - pdf file
