# Supplementary material for: Multiple myeloma-associated hDIS3 mutations cause perturbations in cellular RNA metabolism and suggest hDIS3 PIN domain as a potential drug target
Source: Nucleic Acids Res. 2013 Oct 21;42(2):1270–90. doi: 10.1093/nar/gkt930 (PMC3902924; doi:10.1093/nar/gkt930)
Supplement: Supplementary Data [file supp_gkt930_nar-01708-y-2013-File012.pdf]

Supplementary data

Multiple myeloma-associated *hDIS3* mutations cause perturbations in cellular RNA metabolism and suggest hDIS3 PIN domain as a potential drug target

Rafal Tomecki, Karolina Drazkowska, Iwo Kucinski, Krystian Stodus, Roman Jozef Szczesny, Jakub Gruchota, Ewelina Patrycja Owczarek, Katarzyna Kalisiak and Andrzej Dziembowski

Supplementary Methods

Supplementary Figures S1 – S12

Supplementary Tables S1 – S3

Supplementary References [61-65]

Supplementary Methods

In silico analyses

Multiple amino acid sequence alignment of RNase II/R family members was performed using ClustalX v. 2.0.11 (http://www.clustal.org) and visualized with JalView (http://www.jalview.org). For three-dimensional analysis, structures of the yeast exosome complex and *E. coli* RNase II were acquired from PDB database (PDB IDs: 4IFD and 2IXO, respectively). Domains and residues critical for binding of RNA 3'-end and catalysis were selected according to literature (25,49). Structures were superimposed and visualized using UCSF Chimera software (http://www.cgl.ucsf.edu/chimera).

Bacterial strains

The following *E. coli* strains were used:

- MH1 (*E. coli araD lacX74 galU hsdR hsdM rpsL*),
- dam-/dcm- (New England Biolabs; *E. coli ara-14 leuB6 fhuA31 lacY1 tsx78 glnV44 galK2 galT22 mcrA dcm-6 hisG4 rfbD1 R(zgb210::Tn10)* Tet^S *endA1 rspL136 (*Str^R*) dam13::Tn9 (*Cam^R*) xylA-5 mtl-1 thi-1 mcrB1 hsdR2*),
- BL21-CodonPlus-RIL (Stratagene; *E. coli* B F^- *ompT hsdS*(r_B^- m_B^-) dcm^+ Tet^r *gal endA* Hte [*argU ileY leuW* Cam^r]).

Yeast strains and construction of mutants

Yeast strains employed in this study are listed in Table S1. A *S. cerevisiae* strain encoding fusion of wild-type Dis3p with protein A tag-TEV protease cleavage site and TRP selection marker (ADZY532; referred herein to as wt) was a derivative of haploid BMA64 (*MATa ade 2-1 his3-11,15 leu2-3,112 trp1Δ ura3-1 can1-100*), created by homologous recombination (61). *dis3-D551N* (ADZY123) was

obtained following dissection of a diploid strain BSY1726 (15) and *dis3-D171N* (ADZY531) mutant strains was constructed previously (24). *rrp6Δ* strain was purchased from Euroscarf (acc. no.: Y11777; BY4742 *MATα his3Δ1 leu2Δ0 lys2Δ0 ura3Δ0 YOR001w::kanMX4*).

dis3-V568G, *dis3-A588P*, *dis3-G833R* and *dis3-R847K* mutants were constructed as follows. A diploid BMA64 strain was transformed with DNA fragment containing *DIS3* gene harboring respective mutation, a tag (encompassing TEV protease cleavage site and sequence encoding protein A) and the *TRP* selection marker. First, two parts of the *DIS3* gene were produced in two independent amplifications: 5' – using ADZKD106 forward primer (see Table S2 for primer sequences), complementary to the sequence located upstream the Dis3p coding sequence and suitable reverse primer covering the mutated site (ADZKD134, ADZKD136, ADZKD138 or ADZKD140 for *dis3-V568G*, *dis3-A588P*, *dis3-G833R* and *dis3-R847K* mutant, respectively); and 3' – with appropriate forward primer covering the mutated site (ADZKD133, ADZKD135, ADZKD137 or ADZKD139 for *dis3-V568G*, *dis3-A588P*, *dis3-G833R* and *dis3-R847K* mutant, respectively) and ADZKD107 reverse primer, complementary to the sequence downstream the *TRP* selection marker; total genomic DNA isolated from ADZY532 (wt) strain served as template in all above-mentioned amplifications. The full-length amplicons with desired mutations were obtained with ADZKD106 and ADZKD107 primers in overlap PCR (where products of the 5' and 3' amplifications served as templates), and were subsequently used for transformation. Selected transformants were sporulated and spores were dissected. A spore harboring *dis3-V568G* mutation gave strain ADZY679, *dis3-A588P* – ADZY681, *dis3-G833R* – ADZY783 and *dis3-R847K* – strain ADZY685. The presence of V568G, A588P, G833R and R847K mutations was validated by amplification of the genomic DNA fragments using ADZKD145-ADZKD146 primer pair, followed by digestion of PCR products with *Mls*I, *Apa*I, *Bsh*TI and *Psp*1406I restriction enzymes, respectively. Finally, correctness of the ORF sequence was confirmed by sequencing of the obtained PCR product (see Table S2 for sequences of the primers).

rrp6Δ dis3-G833R and *rrp6Δ dis3-R847K* strains were obtained through crossing *rrp6Δ* strain with either ADZY783 or ADZY685. Spores were dissected and double mutants were selected. Spores harboring *RRP6* deletion together with either *DIS3* G833R or R847K mutation gave strains ADZY732 and ADZY742, respectively.

Diploid strains *DIS3 WT/dis3-D171N G833R-pA* (ADZY713) and *DIS3 WT/dis3-D171N R847K-pA* (ADZY716) were obtained as follows. Diploid BMA64 strain was transformed with a DNA fragment containing *DIS3* gene harboring D171N mutation and G833R or R847K mutation, respectively, a tag (encompassing TEV protease cleavage site and sequence encoding protein A) and the *TRP* selection marker. First, two parts of the *DIS3* gene were amplified: 5' – using ADZKD106-ADZKD141 primer pair and total genomic DNA isolated from ADZY531 strain (with the *DIS3* locus containing D171N mutation) as a template; and 3' – with ADZKD142-ADZKD107 primer pair, employing total genomic DNA isolated from ADZY783 or ADZY685 (with the *DIS3* locus containing G833R or R847K mutation), respectively, as template. The full-length amplicons with the desired mutations were obtained with ADZKD106 and ADZKD107 primers in overlap PCR (where products of the 5' and 3' amplifications served as templates), and were subsequently used for transformation. Selected transformants gave desired strains.

Yeast growth assays

Yeast strains described above were grown in complete YPD medium at 30°C overnight until OD_{600} reached 0.2 before spotting serial dilutions onto YPD plates. Cell growth was analyzed after 60 h of incubation at 25°C, 30°C or 37°C. Analysis of viability for strains harboring mutations in both PIN and RNB domains of Dis3 was carried out by sporulation of diploid strains, followed by tetrad dissection.

Oligonucleotides, plasmids and cloning

Oligonucleotides and plasmids used are listed in Tables S2 and S3, respectively. All restriction enzymes and T4 DNA ligase were from Thermo Scientific. CIP was from New England Biolabs. All DNA purification kits: DNA Plasmid Mini, DNA Plasmid Midi and Gel-Out were from A&A Biotechnology. T4 PNK was from New England Biolabs. PCR reactions were performed with the Phusion DNA polymerase (Thermo Scientific).

Generation of plasmids for production of recombinant hDIS3 variants in E. coli

Plasmids pMM1, pMM2, pMM3, pMM4, pMM5 and pMM6 (for heterologous expression of various hDIS3 versions in *E. coli*) were generated by site-directed mutagenesis with oligonucleotide pairs S477Rfor-S477Rrev, V504Gfor-V504Grev, A524Pfor-A524Prev, G766Rfor-G766Rrev, R780Kfor-R780Krev and I845Vfor-I845Vrev, respectively, using previously described pHEX1 construct (encoding wild-type hDIS3) as template. The presence of S477R, V504G, A524P, G766R, R780K and I845V mutations was confirmed by digestion with *Pst*I, *Mls*I, *Xma*JI, *Bsh*TI, *Psp*1406I and *Eco*147I restriction enzymes, respectively, and sequencing of hDIS3 inserts (see Table S2).

Generation of plasmids utilized for construction of human model cell lines

A multistep cloning procedure for generation of vectors for co-expression of different versions of recoded hDIS3 with FLAG epitope at the C-terminus and sh-miRNAs directed against endogenous hDIS3 mRNA was the following. First, inserts encompassing open reading frames coding for WT, RNB MUT, G766R and R780K variants of hDIS3 were amplified with D3FMluI-D3RB120I primer pair and using pHEX1, pHEX8, pMM4 or pMM5 constructs as respective templates. Next, the inserts were cloned into *Mlu*I and *Bsp*120I sites of BI-16 vector (52), thus replacing hRLUC ORF present therein, with the use *E. coli* MH1 strain (Supplementary Figure S4A). This way, [BI-16’] *hDIS3 WT*, [BI-16’] *hDIS3 RNB MUT*, [BI-16’] *hDIS3 G766R* and [BI-16’] *hDIS3 R780K* transitory vectors were

constructed. hDIS3 inserts were sequenced (refer to Table S2 for sequences of oligonucleotides used in sequencing reactions).

In parallel, we performed a search for 5 miRNA sequences that should specifically and efficiently target endogenous hDIS3 mRNA, using BLOCK-iT™ RNAi Designer tool from Invitrogen (with "*miR RNAi*" option) (http://rnaidesigner.invitrogen.com/rnaiexpress). We chose candidate sequences ranked as 1., 3., 5., 6. and 7. (with the highest scores returned by the program; sequences ranked as 2. and 4. were rejected, as they partially overlapped with 1. and 3.), starting at positions 495., 898., 1159., 1273. and 1404. of hDIS3 ORF, respectively. Basing on the general idea of BLOCK-iT™ Pol II miR RNAi Expression Vector Kits from Invitrogen (http://www.invitrogen.com/site/us/en/home/Products-and-Services/Applications/rnai/Vector-based-RNAi/Pol-II-miR-RNAi-Vectors.html), we then designed two synthetic DNA fragments, each of them encompassing different combination of miRNA sequences listed above – either miR495-miR898-miR1159 or miR1159-miR1273-miR1404 – however only the latter (*tri-miR2*) was utilized in further experiments. It encoded three tandemly positioned shRNAs corresponding to pre-designed miRNAs – so-called sh-miRs, where sense and antisense miRNA sequences were separated by the loop element enabling formation of the hairpin (Supplementary Figure S5A). Each of the sh-miR sequence was flanked at both termini with motifs ensuring correct miRNA processing from the artificial pre-miRNA precursor, following a natural miRNA biogenesis pathway active in human cells (Supplementary Figure S5A). In addition, polyadenylation signal derived from the gene encoding herpes simplex virus thymidylate kinase (HSV-TK-pA) was placed at the 3'-end of this synthetic cassette, allowing for correct termination of transcription in human cells (Supplementary Figure S5A). The cassette contained *Eco*RI/*Sal*I and *Cla*I/*Hin*dIII restriction site combinations at the 5' and 3' extremities, respectively, which were used in subsequent cloning steps (Supplementary Figure S5A). It was synthesized by BlueHeronBio company (http://blueheronbio.com) and inserted between *Eco*RI and *Hin*dIII sites of

pUCAmpMinusMCS vector (Supplementary Figure S4B). Next, a sequence encoding eGFP (allowing for monitoring of expression of the cassette containing artificial pre-miRNA) was amplified in PCR using eGFPFor-eGFPRev primer pair and pEGFP-N1 plasmid (Clontech) as a template and inserted into *Eco*RI and *Sal*I sites of the provided [pUCAmpMinusMCS] *tri-miR 2* plasmid, thus giving [pUCAmpMinusMCS] *eGFP-tri-miR 2* transitory construct (Supplementary Figure S4C). Additionally, a site recognized by *Xma*JI restriction endonuclease was introduced in eGFPFor oligonucleotide upstream the 5'-end of eGFP ORF (Supplementary Figure S4C), which was used at further cloning stage.

In the next phase of construct generation, it was necessary to change the sequence of exogenous hDIS3 ORF in order make it insensitive to miRNA action. To this end, we ordered synthesis of recoded hDIS3 fragment encompassing nucleotides 451.-1457. of the open reading frame (see Supplementary Figure S5B for nucleotide sequence alignment of original and recoded hDIS3 ORF fragment). It is worth noting that this fragment covered all five sites that we initially aimed to target with miRNA (Supplementary Figure S5B), and – on the other hand – it was located outside the region where D487N (RNB MUT), G766R and R780K mutations had been earlier introduced. The idea of recoding was to introduce synonymous mutations into all possible codons (at those positions where degeneration of genetic code could be utilized) within the fragment containing sites recognized by miRNA and taking codon usage frequency into account, so that the sequence would be as much divergent from the initial one as possible (Supplementary Figure S5B). Recoded hDIS3 ORF fragment was synthesized by BlueHeronBio, also as an insert in pUCAmpMinusMCS vector, surrounded with ~30-45 nt-long flanking regions fully complementary to the initial sequence and terminated at both extremities with sites recognized by *Sch*I restriction enzyme – an endonuclease cleaving DNA at some distance from its site and leaving blunt ends following cleavage (Supplementary Figures S4D and S5B). Owing to the presence of such termini, the insert could be excised from the provided [pUCAmpMinusMCS]

rec_hDIS3 plasmid (propagated in *E. coli* MH1 strain) with *Sch*I (Supplementary Figure S4E) and then utilized as a "megaprimer" in overlap extension PCR (62), employing [BI-16'] *hDIS3 WT*, [BI-16'] *hDIS3 RNB MUT*, [BI-16'] *hDIS3 G766R* and [BI-16'] *hDIS3 R780K* plasmids generated in the first step (see above) as templates (Supplementary Figure S4F). We used 0.775 pmol of plasmid DNA and 193.2 pmol of "megaprimer" in overlap extension PCR; 23 cycles of amplification were performed (with denaturation at 98°C, annealing at 55°C and elongation at 72°C carried out for 30'', 1'30'' and 15'30'', respectively, followed by 20' of final elongation at 72°C). Products were digested with *Dpn*I restriction enzyme and introduced to *E. coli* MH1 strain by transformation. This gave rise to [BI-16'] *hDIS3rec WT*, [BI-16'] *hDIS3rec RNB MUT*, [BI-16'] *hDIS3rec G766R* and [BI-16'] *hDIS3rec R780K* transitory constructs, respectively. The presence of recoded fragment was checked by digestion with *Ade*I restriction enzyme (site recognized by this endonuclease disappears after recoding) and sequencing (see Table S2).

The aim of the ultimate cloning stage was to transfer a DNA fragment containing a co-cistron of eGFP coding sequence and pre-miRNA/HSV-TK-pA from [pUCAmpMinusMCS] *eGFP-tri-miR 2* construct to each of the four BI-16 vector derivatives from the previous step, through replacement of the FLUC ORF present in the latter. To this end, all plasmids were propagated in *E. coli* dam-/dcm- strain prior to the standard cloning procedure, utilizing *Xma*JI and *Cla*I restriction sites, followed by transformation of the ligation products into *E. coli* MH1 strain (Supplementary Figure S4G). This eventually led to the generation of final constructs: pMM7-pMM10 (see Table S3). Both *hDIS3rec* and *eGFP-tri-miR 2* inserts were sequenced (see Table S2 for sequences of sequencing primer).

pMM11-pMM14 (Table S3) constructs were generated by site-directed mutagenesis using D3PINF-D3PINR oligonucleotide pair and pMM7-pMM10 plasmids as respective templates.

Generation of constructs required for conditional DIS3 knockout in DT40 cells

pLoxPuro and pLoxBsr (63) were used as entry vectors for targeted integration. These plasmids carry selection markers (puromycin or blasticidin, respectively) flanked with two *loxP* sites allowing for recycling of selection marker through Cre-mediated recombination. Expression cassette containing DIS3 coding sequence was derived from pcDNA5/FRT/TO (Invitrogen) derivative with cloned human DIS3 ORF tagged with FLAG epitope at the C-terminus (referred to as pHEX18 in ref. 26). Human gene was selected with the aim of decreasing non-specific homologous recombination in DT40 cell line. Expression cassette was under CMV (cytomegalovirus) promoter, which was previously shown to be functional in avian cell lines (64).

A region containing CMV promoter, hDIS3 ORF with FLAG tag and BGH polyA signal was amplified in PCR with primer pair CMV_F-BGH_R using pHEX18 as template. The PCR product was cloned into pLoxPuro or pLoxBsr linearized with *Nhe*I restriction enzyme using SLIC (Sequence and Ligase Independent Cloning) method (65). Obtained clones were sequenced with use of CMV_R, HD3F883, HD3F1848, HD3F2429, HD3R1021, HD3R1592, HD3R2443, RSZ174 and RSZ176 in order to avoid any mutations. This way, [pLoxPuro] *hDIS3* and [pLoxBsr] *hDIS3* transitory constructs were created.

Chicken *DIS3* arms required for homologous recombination were amplified using genomic DNA of wild-type DT40 cells. *DIS3* left arm was obtained in PCR with DIS3_LF- DIS3_LR primer pair whereas *DIS3* right arm was obtained in PCR with DIS3_RF-DIS3_RR primer pair. *DIS3* left arm was cloned using standard cloning methods into *Not*I restriction site present in [pLoxPuro] *hDIS3*, while right arm was added subsequently using SLIC method. This eventually led to the generation of pMM15 vector (Table S3). pMM16 construct (Table S3) was created by SLIC-mediated cloning of DNA fragment covering both chicken *DIS3* arms and complete *hDIS3* expression cassette amplified using CMV_F-BGH_R primer pair and pMM15 as a template into *Nhe*I restriction site of pLoxBsr.

Other plasmids

pLsm1 plasmid, kindly provided by Prof. Joanna Kufel, which was used as a template for *in vitro* transcription, is pGEM-T Easy with insert encompassing nucleotides 1-387 of the open reading frame corresponding to *A. thaliana* Lsm1 protein.

Purification of the exosomes from yeast strains

The yeast strains producing protein A-tagged Dis3 variants were grown in 8 l of YPD to OD_{600}=2. Following centrifugation at 5000 rpm for 5 minutes at 4°C, the cell pellet was resuspended in 30 ml of lysis buffer (40 mM Hepes-KOH pH=8.0; 250 mM NaCl; 1 mM DTT), frozen in liquid nitrogen and stored at -80°C. Cells were broken in a laboratory blender chilled with dry ice. The homogenate was melt in the presence of protease inhibitors and centrifuged in 35Ti rotor (Beckman) in a Beckman ultracentrifuge at 20000 rpm for 20 minutes at 4°C. Supernatant was spun again at 32000 rpm for 75 min at 4°C and afterwards the supernatant was dialyzed for 3 hours against 3 liters of buffer D (40 mM Hepes-KOH pH=8.0; 500 mM NaCl; 1mM DTT; 1 mM PMSF; 20 mM benzamidine-HCl; 20% glycerol). The dialyzed extract was incubated on a rotating wheel overnight with 1 ml of IgG Sepharose 6 Fast Flow (GE Healthcare) equilibrated with lysis buffer, in the presence of 0.1% reduced Triton X-100 (rTX-100) at 4°C. The beads were transferred onto the column compatible with ÄKTA Purifier system (GE Healthcare) and washed with 30 ml of IPP500 (10 mM Tris-HCl pH=8.0; 500 mM NaCl; 0.1% rTX-100), followed by wash with 30 ml of TEV protease cleavage buffer (10 mM Tris-HCl pH=8.0; 500 mM NaCl; 0.5 mM EDTA; 1 mM DTT). The on-column TEV protease cleavage with home-made enzyme was performed for 4 hours at 18°C. The eluate from IgG beads was collected and further purified by size-exclusion chromatography on Superdex S-200 column (GE Healthcare) using ÄKTA Purifier and gel filtration buffer (10 mM Tris-HCl pH=8.0; 150 mM NaCl). Fractions

containing the exosome were pooled, concentrated on Vivaspin 500 column (Sartorius) and analyzed by SDS-PAGE.

Multiple myeloma cell lines, genomic DNA isolation and sequencing of *hDIS3* gene

ATCC or DKFZ H929 and ATCC SKMM1 (the latter kindly provided by Dr W. Michael Kuehl, M.D.; Center for Cancer Research; National Cancer Institute; Bethesda) cell lines were obtained from Department of Immunology, Clinical Hospital, Medical University of Warsaw. Total genomic DNA from these cell lines was extracted and purified with TRI Reagent (Sigma-Aldrich) followed by ethanol precipitation, according to the manufacturer protocol. DNA from COLO677, JIM1, JIM3, KARPAS417, RPMI8226, U226B1 cell lines was purchased from The Health Protection Agency Culture Collections (HPA, UK).

For DNA sequencing, two fragments of *hDIS3* gene covering two parts of RNB domain and flanking regions were amplified – fragment of 1289 bp covering exons 9 to 11 was amplified with use of primer pair DIS3_9f-DIS3_11r, while fragment of 1182 bp covering exons 17 to 19 was amplified using primer pair DIS3_17f-DIS3_19r (refer to Table S2 for primer sequences). Primers were designed using human genomic sequence: (GenBank accession number NC_000013; region 73329540…73356344), Oligo software (MBI, USA) and checked with Primer-BLAST (available online at: http://www.ncbi.nlm.nih.gov/tools/primer-blast/) to ensure that only target sequences should be amplified. PCR reactions were optimized by temperature and primer concentration gradient to achieve only specific bands and to reduce primer-dimers to minimum. 40 ng of genomic DNA, 1x HF Buffer, 0.5 µM of each primer, 0.25 mM of dNTPs and 0.02 U/µl of Phusion DNA Polymerase (Thermo Scientific) were used in 25 µl reaction. No template controls were performed for all amplifications. Cycling program was as follows: step of initial denaturation and enzyme activation in 98°C for 3 minutes was followed by 35 cycles of amplification (with denaturation at 98°C, annealing at

64^oC and elongation at 72^oC, carried out for 15'', 21'' and 25'', respectively). Quality of PCR products was verified by visualizing them in ethidium bromide-stained 1.5% agarose gel.

Prior to DNA sequencing, 5 µl of each PCR product was "purified" from all remaining oligos, primer-dimers and other ssDNAs by 15 minutes of incubation at 37^oC with 10 U of Exonuclease I and 1 U of FastAP™ Thermosensitive Alkaline Phosphatase (both from Thermo Scientific), followed by 15 minutes of thermal inactivation at 85^oC.

Eventually, both strands of PCR products were sequenced using 5 pmoles of each primer, previously employed in amplification. In the case of low-quality reads, internal sequencing primers – DIS3_10f for PCR product amplified with DIS3_9f-DIS3_11r primer pair and DIS3_18r for PCR product amplified with DIS3_17f-DIS3_19r primer pair – were additionally used.

Sequencing results were preliminary screened with FinchTV software (Geospiza) and then thoroughly analyzed with Mutation Surveyor 4.0 software (SoftGenetics), which allows to detect minor alleles (down to 5% basing on the analysis of both strands), in search for the presence of mutations and other differences comparing to GenBank reference sequence.

Isolation of genomic DNA from DT40 cell lines

Approximately $5x10^6$ of DT40 cells were harvested by centrifugation at 300xg for 4 minutes, washed in PBS and spun down again. Cell pellet was resuspended in RSB buffer (10 mM Tris-HCl pH=7.4, 10 mM NaCl, 10 mM EDTA pH=8.0). 8 µl of Proteinase K (Bioline; 20 mg/ml), 6 µl of RNase A (Sigma-Aldrich; 10 mg/ml) and 40 µl of 10% SDS were added to the cell suspension. Samples were incubated in a thermomixer at 37^oC with mixing at 700 rpm. After 2 hours of incubation temperature was increased to 56^oC for 3 hours. Additionally, samples were mixed in 30 min. intervals. When lysis was complete, equal volume of phenol solution pH=8.0 (Sigma-Aldrich) was added and mixed in hands for 5 minutes and additionally on a rotating wheel for 1 hour and centrifuged at 21000xg, 4^oC for 15

minutes. Aqueous phase was transferred to new tubes and after addition of equal volume of phenol/chloroform (1:1; v:v), samples were mixed in hands for 5 minutes and additionally put onto a rotating wheel for 1 hour and centrifuged as above. Aqueous phase was transferred into new tubes and after addition of equal volume of chloroform, samples were incubated on a rotating wheel for 1 hours and centrifuged at 21000xg, RT for 5 minutes. Afterwards, DNA was precipitated from aqueous phase using 0.1 volume of 10 M ammonium acetate, 1.4 volumes of isopropanol for 1 hour at RT with mixing in 15 min. intervals and centrifuged at 21000xg, RT for 15 minutes. DNA pellets were resuspended and incubated for 15 minutes in 1 ml of 75% EtOH and centrifuged at 21000xg, 4°C for 5 minutes. DNA pellets were air-dried and suspended in 30 µl of H_2O.

Immunolocalization analysis

2x10^{4} cells of HEK293 Flp-In T-Rex-derived stable cell lines were plated onto Nunc Lab-Tek II 8-well chamber slides (Thermo Scientific), pre-coated with Poly-D-Lysine (Sigma-Aldrich), in a medium with doxycycline (100 ng/ml) to induce protein expression. Following aspiration of the medium, cells were fixed with 3.7% formaldehyde/5% sucrose in PBS for 25 minutes, permeabilized with 0.5% Triton X-100/10% FBS solution in PBS for 15 min and blocked for 30 minutes with 10% FBS solution in PBS (blocking solution). Cells were incubated in blocking solution, first with primary antibody for 1 h and then with secondary antibody coupled to fluorophore for 1 h in the dark. Finally, cells were stained with DAPI (Invitrogen) by incubation in a 2.5 µg/ml solution in PBS for 5 min and washed as above. All procedures were performed at 25°C and cells were washed 3 times with PBS between each step. After the final wash coverslips were mounted on chamber slides in ProLong Gold antifade reagent (Invitrogen), left in the dark overnight at 25°C and then stored at 4°C until microscopic analysis.

The following antibodies were used (dilutions in parentheses): 1) primary – mouse monoclonal anti-FLAG (M2) (Sigma-Aldrich; F3165) (1:200); rabbit polyclonal anti-hDIS3 (Sigma-Aldrich;

HPA039281, lot: R37348) (1:100); rabbit polyclonal anti-fibrillarin (Abcam; ab5821) (1:150); 2) secondary – Alexa Fluor 635-conjugated goat anti-mouse, Alexa Fluor 555-conjugated goat anti-rabbit IgG, Alexa Fluor (both from Molecular Probes) (1:800). Imaging was performed on a FluoView FV1000 system with spectral detectors (Olympus), using appropriate emission filters and a 60x/1.40 oil immersion objective lens. Images were processed using the FluoView software.

siRNA transfection

siRNA-mediated knockdown was done using stealthRNA and Lipofectamine RNAiMAX (Invitrogen) according to the manufacturer's instructions. The stealthRNA oligo against hRRP6 (ID HSS182420) or negative control (Stealth™ RNAi Negative Control Low GC) (both from Invitrogen) were used at the final concentration of 20 nM. The cells were grown in the absence or presence of doxycycline for additional 60 hours before harvesting.

Substrate preparation for *in vitro* biochemical assays

ss17-A_{14}, ss44 and compl (see Table S2 for sequences) oligoribonucleotides (Invitrogen) were purified by electrophoresis in a 10% polyacrylamide gel containing 8 M urea. Oligoribonucleotides were detected by ultraviolet shadowing, excised and the RNAs were eluted overnight at 25°C in a buffer containing 100 mM Tris-HCl, pH 8.0; 12.5 mM EDTA; 150 mM NaCl; 1% SDS and an equal volume of phenol:chloroform (1:1; v:v).

5′-end labeling of substrates was performed with T4 PNK (NEB) and [γ-^{32}P]ATP (GE Healthcare) according to the manufacturer's instructions.

All labeled single-stranded RNA substrates were further purified following electrophoresis in 10% denaturing polyacrylamide gels with 8 M urea as described above.

ds17-$(A)_{14}$ partial RNA duplex was prepared by mixing nonradioactive single-stranded oligoribonucleotides ss17-$(A)_{14}$ and compl in a 1:1.2 molar ratio, with addition of radiolabeled ss17-$(A)_{14}$ oligo in a buffer containing 10 mM Tris-HCl, pH 8.0 and 20 mM KCl. The mixture was then incubated at 95°C for 7 minutes and allowed to cool down slowly to room temperature.

Internally labeled RNA was obtained by *in vitro* transcription performed using pLsm1 plasmid (see above) digested with *Sal*I as a template, in the presence of [α-^{32}P]UTP and T7 RNA polymerase (NEB). Radiolabeled transcript was extracted with phenol:chloroform and chloroform alone and further purified by centrifugation in Spin Modules (MP Biomedicals) packed with Sephadex G-50 (Bio-Rad).

Polysome gradients and RNA isolation

Stable cell lines were grown overnight in a medium containing doxycycline on one ø145 mm plate until reaching ~95% confluence. Cells were treated with cycloheximide (Sigma-Aldrich; 200 µg/ml) at 37°C for 15 minutes, harvested by trypsinization, spun down for 1 minute at 500xg; 4°C and then washed 3 times with ice-cold PBS containing 100 µg/ml cycloheximide. After final wash and complete removal of PBS, the cells were suspended in 0.5 ml of lysis buffer (10 mM Hepes-KOH, pH=7.5; 100 mM KCl; 2.5 mM $MgCl_2$; 1 mM DTT; 100 µg/ml cycloheximide; 1 mg/ml heparin (Sigma-Aldrich); 1% reduced Igepal-CA630 (Sigma-Aldrich); 80 u/µl RiboLock^{TM} RNase Inhibitor (Thermo Scientific); 1 x Protease Inhibitor Cocktail, Complete EDTA-free (Roche)), lysed by thorough pipetting and incubation for 15 minutes at 4°C on a rotating wheel. Lysates were then centrifuged for 10 minutes at 10000xg; 4°C and RNA concentration in collected supernatants was measured using Nanodrop 2000c device (Thermo Scientific). 8 OD_{260} units of cytoplasmic lysates in 500 µl of lysis buffer were layered onto 7-47% sucrose gradients (prepared using filtered sucrose solutions in lysis buffer, lacking detergent and ribonuclease inhibitor) and ultracentrifuged for 2 hours at 39000xg; 4°C in SW-41Ti rotor (Beckman

Coulter). Subsequently, 0.5 ml fractions were collected from each gradient by pumping 60% sucrose solution (prepared as above) to the bottom of tubes and OD_{260} was monitored on ÄKTA Purifier.

For RNA isolation from polysome gradients, collected fractions were thoroughly mixed with 650 µl of phenol : guanidine thiocyanate (1:1) by vortexing and incubated at 65°C for 8 minutes. Then, 320 µl of chloroform and 120 µl of 3M sodium acetate (pH=5.2) were added, followed by vortexing and centrifugation for 5 minutes at 13200 rpm. Samples were extracted twice with chloroform and RNA was precipitated from aqueous phase with isopropanol, washed with 75% ethanol and suspended in 20 µl of RNase-free water. RNA samples were subsequently pooled and analyzed by northern-blotting.

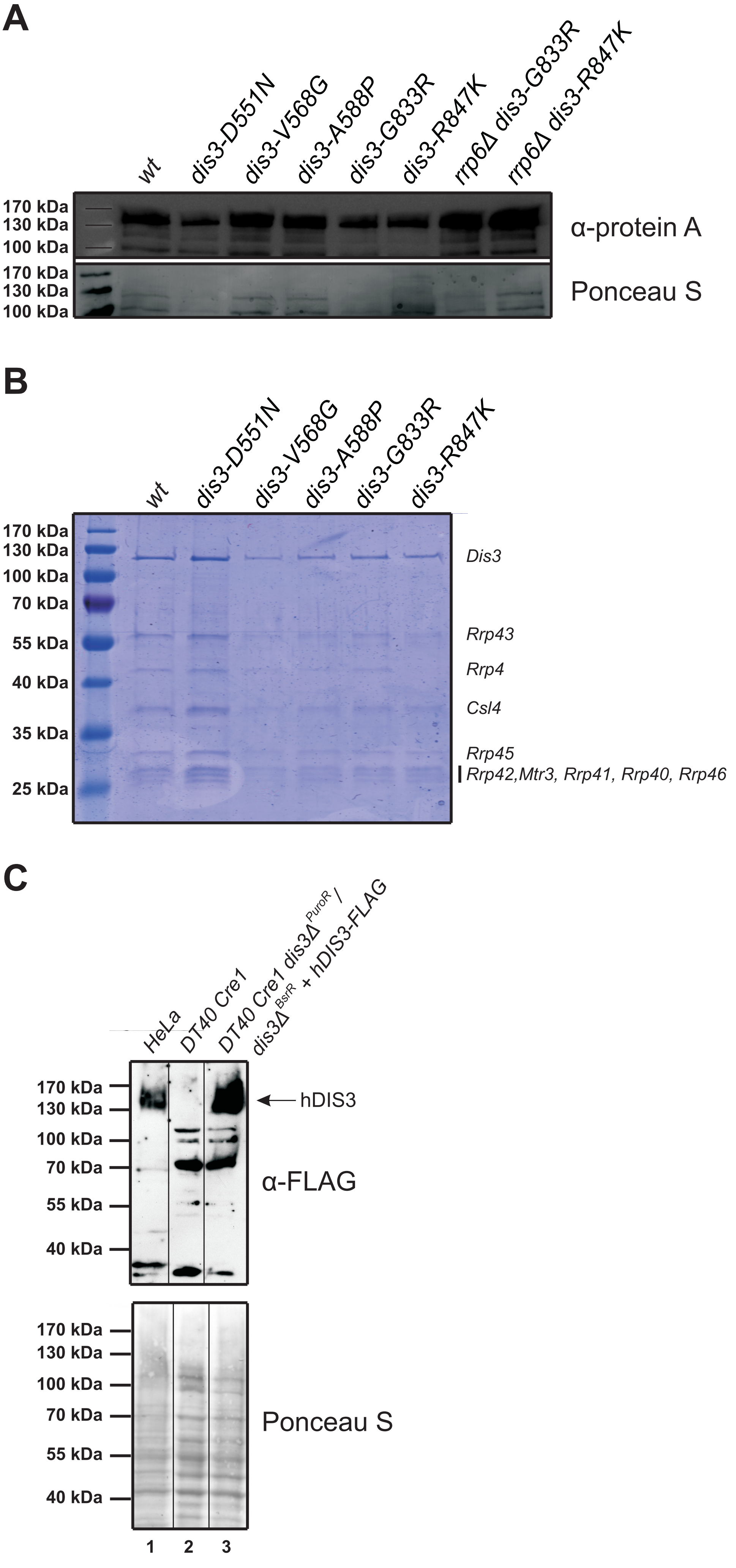
A
wt
dis3-D551N
dis3-V568G
dis3-A588P
dis3-G833R
dis3-R847K
rrp6Δ dis3-G833R
rrp6Δ dis3-R847K
170 kDa
130 kDa
100 kDa
α-protein A
170 kDa
130 kDa
100 kDa
Ponceau S
B
wt
dis3-D551N
dis3-V568G
dis3-A588P
dis3-G833R
dis3-R847K
170 kDa
130 kDa
100 kDa
70 kDa
55 kDa
40 kDa
35 kDa
25 kDa
Dis3
Rrp43
Rrp4
Csl4
Rrp45
Rrp42,Mtr3, Rrp41, Rrp40, Rrp46
C
HeLa
DT40 Cre1
DT40 Cre1 dis3Δ PuroR / dis3Δ BsrR + hDIS3-FLAG
170 kDa
130 kDa
100 kDa
70 kDa
55 kDa
40 kDa
hDIS3
α-FLAG
170 kDa
130 kDa
100 kDa
70 kDa
55 kDa
40 kDa
Ponceau S
1
2
3

Supplementary Figure S1. DIS3 proteins are efficiently expressed in constructed yeast strains and chicken cell lines. (**A**) Western-blot analysis of the expression of different protein A-tagged Dis3 proteins in constructed yeast strains. Total protein samples were isolated from various yeast strains, separated in SDS-PAGE gel and transferred onto nitrocellulose membrane, which was then probed with peroxidase-anti-peroxidase antibody. Masses of individual bands of molecular weight protein ladder are indicated on the left. (**B**) Mutant Dis3 variants are stable and efficiently incorporated into the exosome core. Exosome complexes were purified from the indicated yeast strains on IgG beads, using protein A-tagged Dis3 variants as baits, and subsequently analyzed in SDS-PAGE gel, stained with Coomassie. Positions of the exosome subunits are marked on the right. Masses of individual bands of molecular weight protein ladder are indicated on the left. (**C**) Western-blot analysis of the expression of hDIS3 in different cell lines. Total protein samples were isolated from human HeLa cells (*lane 1*), parental chicken DT40 Cre1 cells (*lane 2*) or stable DT40 Cre1 homozygotic *DIS3* knockout cell line bearing expression cassette encoding hDIS3-FLAG fusion (*lane 3*), separated in SDS-PAGE gel and transferred onto nitrocellulose membrane, which was stained with Ponceau S-Red (loading control; *bottom*) and subsequently probed with anti-FLAG antibody (*top*). Masses of individual bands of molecular weight protein ladder are indicated on the left. Position of hDIS3 protein is marked with arrow.

A

cell line	*this study* DNA change	aminoacid substitution	*Chapman et al, 2011* DNA change	aminoacid substitution
COLO677	none	none	*not analyzed*	
JIM1	none	none	*not analyzed*	
JIM3	none	none	none	none
KARPAS417	none	none	*not analyzed*	
RPMI8226	none	none	none	none
U226B1	none	none	*not analyzed*	
H929	none	none	*c.G1570C*	p.A524P
SKMM1	*c.A2533G*	p.I845V	*c.A2533G*	p.I845V

B

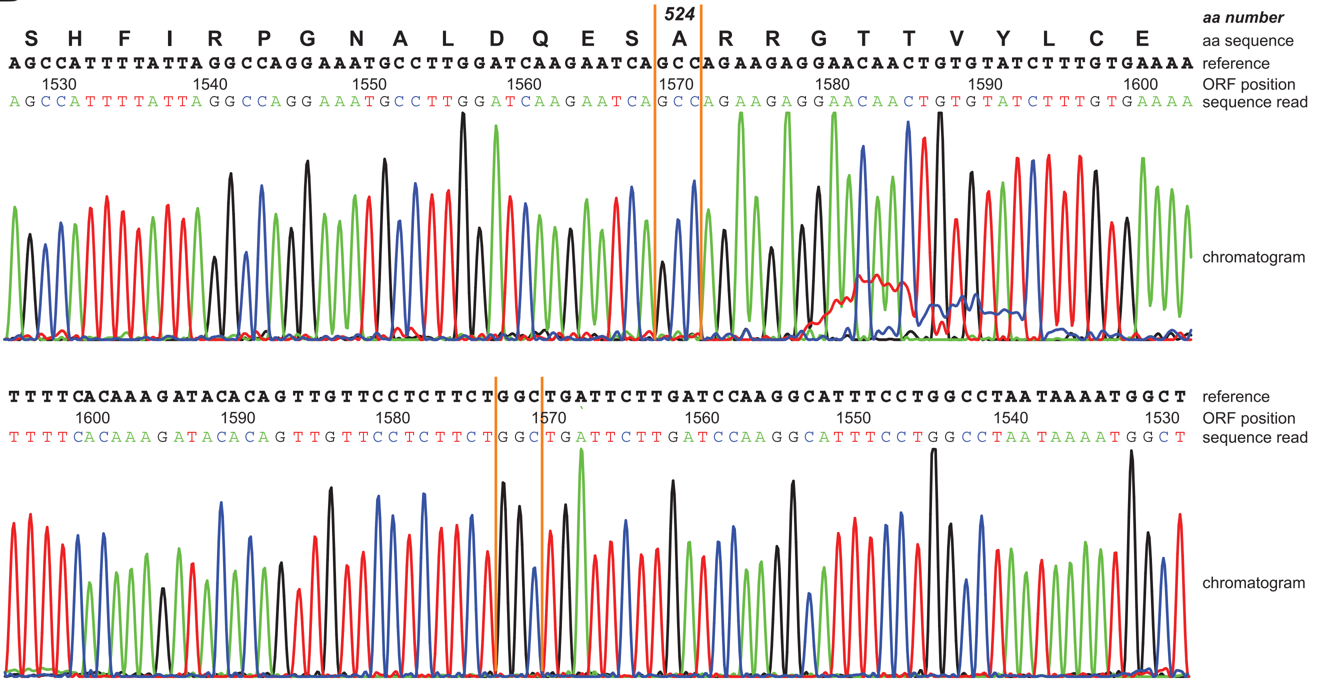

C

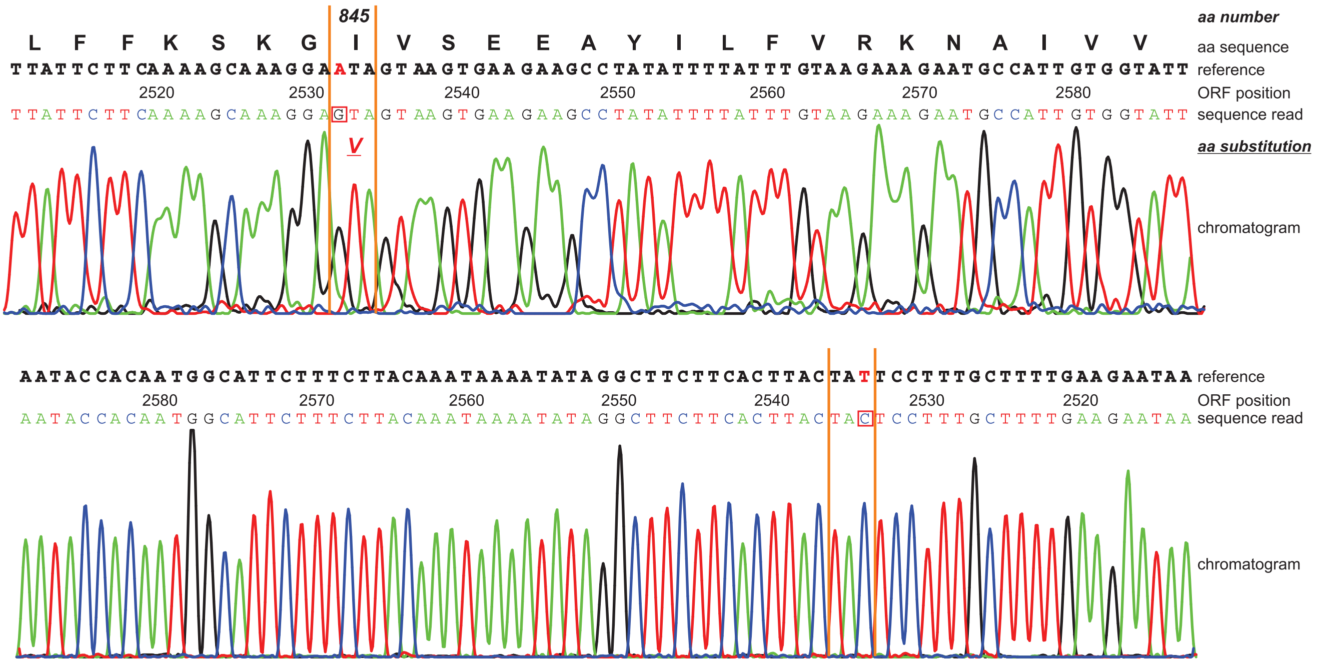

Supplementary Figure S2. Sequencing of *hDIS3* alleles in different multiple myeloma cell lines. (**A**) Comparison of the results obtained in this study with those reported in ref. 4; in three out of four cell lines that were analyzed in both reports the conclusions were consistent, including detection of *A2533G* mutation (leading to I845V amino acid change) in SKMM1 cell line; however, we were unable to confirm the presence of *G1570C* mutation (resulting in A524P substitution) in H929 cell line. (**B**) Results of *hDIS3* sequencing in H929 cell line; both coding strand and non-coding strand were sequenced (presented in the *upper* and *lower* chromatogram, respectively); borders of the codon encoding A524 are marked with orange vertical lines; no substitution of guanine in position 1570 of the ORF was detected. (**C**) Results of *hDIS3* sequencing in SKMM1 cell line; both coding strand and non-coding strand were sequenced (presented in the *upper* and *lower* chromatogram, respectively); borders of the codon encoding I845 are marked with orange vertical lines; *A2533G* leads to the change of ATA codon (corresponding to isoleucine) in GTA triplet (corresponding to valine).

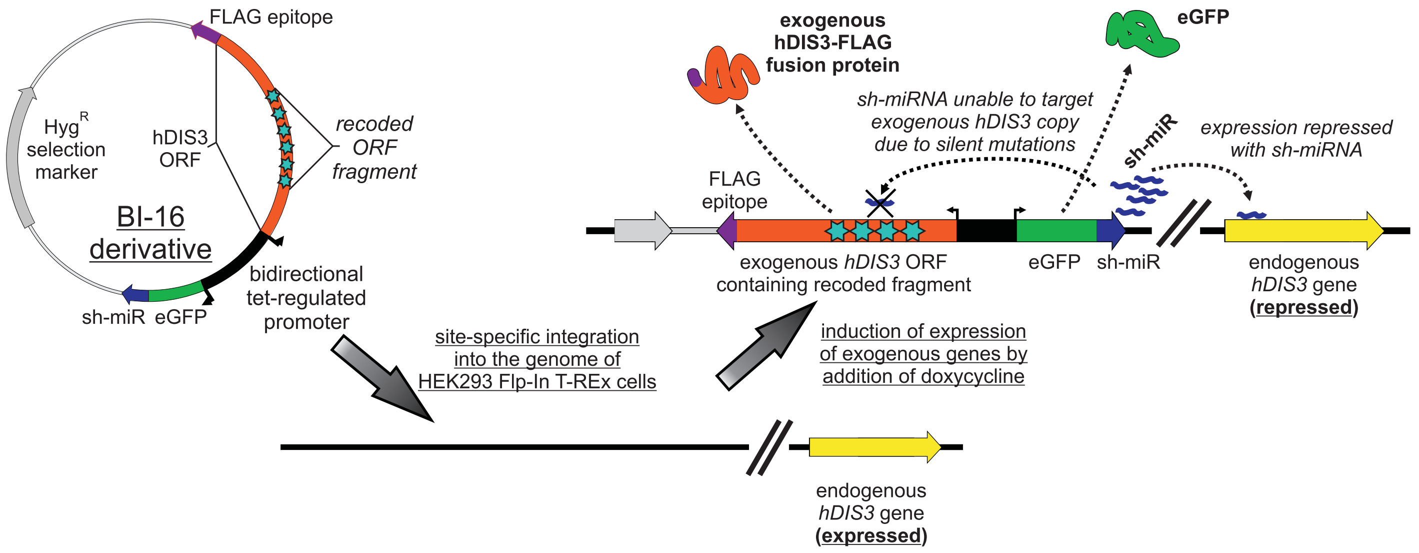

Supplementary Figure S3. General principle of the constructed human cellular model. Derivatives of the BI-16 vector (compatible with the Flp-In™ T-Rex™ system from Invitrogen) containing wild-type or mutated FLAG-tagged hDIS3 ORF with a recoded region and an eGFP-sh-miRNA fusion, both under control of the bidirectional tetracycline-regulated promoter, were integrated into the HEK293 Flp-In T-REx cell line genome. The FLAG-tagged hDIS3 ORF included a recoded sh-miRNA-insensitive sequence, rendering it insusceptible to sh-miRNA silencing. Upon induction with doxycycline, each stable cell line produced a given variant of hDIS3-FLAG fusion and sh-miRNA, thereby silencing expression of only the endogenous *hDIS3*. Production of sh-miRNA was monitored by eGFP expression.

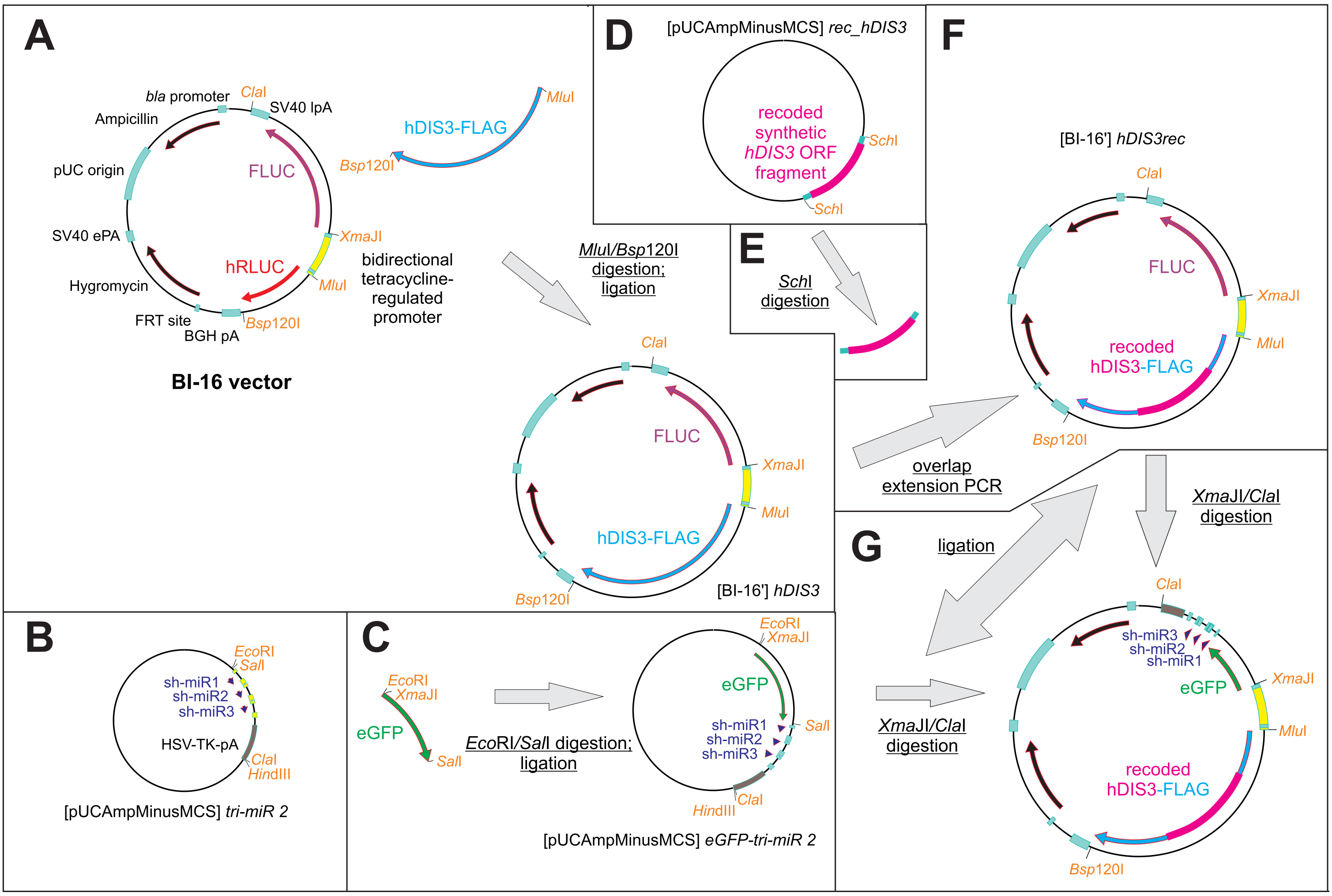

Supplementary Figure S4. Outline of the cloning strategy, which was applied for the construction of vectors utilized for establishment of the model human cell lines producing different variants of hDIS3 protein. (**A**) An insert coding for respective FLAG-tagged hDIS3 version was cloned into *Mlu*I/*Bsp*120I of the BI-16 vector. (**B**) In parallel, synthetic construct bearing three sh-miR sequences designed to specifically silence the expression of endogenous *hDIS3*, was ordered. (**C**) Subsequently, eGFP coding sequence was cloned upstream sh-miRs, to enable monitoring of their expression. (**D**) Next, a recoded ORF fragment encompassing sequences targeted by pre-designed sh-miRs was synthesized. (**E**),(**F**) Following its excision from the synthetic construct (E), it was used as a megaprimer in the overlap PCR (F), leading to the substitution of the ORF fragment in the insert corresponding to the exogenous hDIS3-FLAG into the one that should be insensitive to sh-miRNA action. (**G**) An insert encompassing fusion of eGFP with sh-miRs was cloned into *Xma*JI/*Cla*I sites of BI-16 derivatives from (A), thus generating final constructs enabling simultaneous: 1) expression of a given version of exogenous hDIS3-FLAG and 2) downregulation of endogenous *hDIS3*.

Further details on the cloning procedure are described in the Supplementary Materials and Methods above.

A

GAATTCATATA**GTCGAC**CAGTGGATCCTGGAGGCTTGCTGAAGGCTGTATGCTGTAATGTGGAAGCCTGT
CTGGTGTTTTGGCCACTGACTGACACCAGACACTTCCACATTACAGGACACAAGGCCTGTTACTAGCACT
CACATGGAACAAATGGCCCAGATCCTGGAGGCTTGCTGAAGGCTGTATGCTGTTCTGTTTCAGTCTCTTT
CTCGTTTTGGCCACTGACTGACGAGAAAGACTGAAACAGAACAGGACACAAGGCCTGTTACTAGCACTCA
CATGGAACAAATGGCCCAGATCCTGGAGGCTTGCTGAAGGCTGTATGCTGATACACAGATGCCTCAGGTC
TGTTTTGGCCACTGACTGACAGACCTGACATCTGTGTATCAGGACACAAGGCCTGTTACTAGCACTCACA
TGGAACAAATGGCCCAGATCTGGCCGCACTCGAGATATCTAGTGATCTAGAGGGCCCGCGGTTCGCTGAT
GGGGGAGGCTAACTGAAACACGGAAGGAGACAATACCGGAAGGAACCCGCGCTATGACGGCAATAAAAAG
ACAGAATAAAACGCACGGGTGTTGGGTCGTTTGTTCATAAACGCGGGGTTCGGTCCCAGGGCTGGCACTC
TGTCGATACCCCACCGTGACCCCATTGGGGCCAATACGCCCGCGTTTCTTCCTTTTCCCCACCCCACCCC
CCAAGTTCGGGTGAAGGCCCAGGGCTCGCAGCCAACGTCGGGGCGGCAGGCCCTGCCATAGC**ATCGAT**CG
C**AAGCTT**

B

```
hDIS3        (451)              ...GTAGCAGCAAAATGGTACAATGAACATTTG
hDIS3rec                     GAGTCATATAGTAGCAGCAAAATGGTACAATGAACATTTG
                                       ******************************

             (151)                   ...  V   A   A   K   W   Y   N   E   H   L

                                   miR495
hDIS3        AAAAAAATGTCAGCAGACAACCAGCTGCAAGTTATCTTCATAACAAATGACAGGAGAAAC
hDIS3rec     AAAAAAATGTCAGCCGATAATCAACTCCAGGTGATATTTATCACCAACGATCGCCGCAAT
             ************** ** ** ** ** ** ** ** ** ** ** ** **  *  * **

               K  K  M  S  A  D  N  Q  L  Q  V  I  F  I  T  N  D  R  R  N

hDIS3        AAAGAGAAAGCCATAGAAGAAGGAATACCAGCTTTCACTTGTGAAGAATATGTAAAGAGC
hDIS3rec     AAGGAAAAGGCAATCGAGGAGGGCATCCCCGCATTTACATGCGAGGAGTACGTCAAATCC
             ** ** ** ** ** ** ** ** ** ** ** ** ** ** ** ** ** ** **   *

               K  E  K  A  I  E  E  G  I  P  A  F  T  C  E  E  Y  V  K  S

hDIS3        CTAACTGCTAACCCCGAACTCATAGATCGTCTTGCTTGTTTGTCTGAAGAAGGGAATGAA
hDIS3rec     CTCACAGCAAATCCAGAGCTGATCGACAGACTGGCATGCCTCAGCGAGGAGGGCAACGAG
             ** ** ** ** ** ** ** ** **  * ** ** **  *    ** ** ** ** **

               L  T  A  N  P  E  L  I  D  R  L  A  C  L  S  E  E  G  N  E

hDIS3        ATAGAAAGTGGAAAAATAATATTTTCAGAGCATCTTCCCTTAAGTAAGCTACAGCAAGGC
hDIS3rec     ATCGAGAGCGGCAAGATCATCTTCAGCGAACACCTGCCACTGTCCAAACTCCAACAGGGA
             ** ** ** ** ** ** ** **    ** ** ** **  *    ** ** ** ** **

               I  E  S  G  K  I  I  F  S  E  H  L  P  L  S  K  L  Q  Q  G

hDIS3        ATAAAATCTGGTACATACCTTCAAGGAACATTTAGAGCTAGCAGGGAAAATTACTTGGAA
hDIS3rec     ATCAAGAGCGGAACCTATCTGCAGGGCACCTTCCGGGCATCCCGCGAGAACTATCTCGAG
             ** **    ** ** ** ** ** ** ** **  * **   * * ** ** **  * **

               I  K  S  G  T  Y  L  Q  G  T  F  R  A  S  R  E  N  Y  L  E

hDIS3        GCTACAGTATGGATTCATGGCGACAGTGAAGAAAATAAAGAGATAATCTTACAGGGACTT
hDIS3rec     GCAACCGTCTGGATCCACGGAGATTCCGAGGAGAACAAGGAAATCATTCTGCAAGGCCTG
             ** ** ** ***** ** ** **    ** ** ** ** ** ** **  * ** ** **

               A  T  V  W  I  H  G  D  S  E  E  N  K  E  I  I  L  Q  G  L

hDIS3        AAACATTTAAACAGAGCTGTTCACGAAGATATTGTGGCTGTGGAGCTTCTCCCCAAGAGT
hDIS3rec     AAGCACCTGAATCGGGCAGTGCATGAGGACATCGTCGCAGTCGAACTGCTGCCAAAATCC
             ** **  * **  * ** ** ** ** ** ** ** ** ** ** ** ** ** ** **

               K  H  L  N  R  A  V  H  E  D  I  V  A  V  E  L  L  P  K  S
                  miR898
hDIS3        CAGTGGGTAGCACCATCTTCTGTGGTTTTACATGATGAAGGTCAAAATGAAGAAGATGTG
hDIS3rec     CAATGGGTCGCCCCCAGCAGCGTCGTGCTGCACGACGAGGGACAGAACGAGGAGGACGTC
             ** ***** ** **        ** **  * ** ** ** ** ** ** ** ** ** **

               Q  W  V  A  P  S  S  V  V  L  H  D  E  G  Q  N  E  E  D  V

hDIS3        GAGAAAGAAGAAGAGACAGAACGAATGCTTAAGACTGCTGTAAGCGAGAAAATGTTGAAG
hDIS3rec     GAAAAGGAGGAGGAAACCGAGAGGATGCTGAAAACAGCAGTCTCCGAAAAGATGCTCAAA
             ** ** ** ** ** ** **  * ***** ** ** ** **   *** ** *** * **

               E  K  E  E  E  T  E  R  M  L  K  T  A  V  S  E  K  M  L  K

hDIS3        CCTACAGGTAGAGTTGTAGGAATAATAAAAAGGAATTGGAGACCATATTGTGGCATGCTT
hDIS3rec     CCAACCGGACGGGTGGTCGGCATCATTAAGCGCAACTGGCGGCCCTACTGCGGAATGCTG
             ** ** **  * ** ** ** ** ** **  * ** *** * ** ** ** ** *****

               P  T  G  R  V  V  G  I  I  K  R  N  W  R  P  Y  C  G  M  L
```

```
hDIS3        TCCAAGTCTGACATTAAGGAGTCAAGAAGACATCTCTTTACACCTGCTGATAAGAGAATC
hDIS3rec     AGCAAAAGCGATATCAAAGAAAGCCGGCGGCACCTGTTCACCCCAGCAGACAAACGGATT
               ***    ** ** ** **     *  * ** ** ** ** ** ** ** **  * **

               S  K  S  D  I  K  E  S  R  R  H  L  F  T  P  A  D  K  R  I
                                       miR1159
hDIS3        CCTCGAATTCGCATAGAAACCAGACAGGCTTCCACATTAGAAGGACGGAGAATTATTGTT
hDIS3rec     CCAAGGATCAGAATCGAGACACGGCAAGCAAGCACCCTGGAGGGCAGACGGATCATCGTA
             **  * **  * ** ** **  * ** **   ***  * ** **  *  * ** ** **

               P  R  I  R  I  E  T  R  Q  A  S  T  L  E  G  R  R  I  I  V

hDIS3        GCTATTGATGGTTGGCCCAGAAATTCCAGATATCCAAATGGACACTTTGTGAGAAATTTA
hDIS3rec     GCAATCGACGGATGGCCACGGAACAGCCGGTACCCCAACGGCCATTTCGTCCGGAACCTG
             ** ** ** ** *****  * **   * * ** ** ** ** ** ** **  * **  *

               A  I  D  G  W  P  R  N  S  R  Y  P  N  G  H  F  V  R  N  L
                                  miR1273
hDIS3        GGTGATGTTGGAGAGAAAGAGACTGAAACAGAAGTTTTGTTACTTGAACACGATGTTCCC
hDIS3rec     GGGGACGTGGGCGAAAAGGAAACAGAGACCGAGGTGCTCCTGCTGGAGCATGACGTGCCA
             ** ** ** ** ** ** ** ** ** ** ** **  *  * ** ** ** ** ** **

               G  D  V  G  E  K  E  T  E  T  E  V  L  L  L  E  H  D  V  P

hDIS3        CATCAGCCTTTTTCACAGGCTGTTCTTAGTTTTCTGCCAAAGATGCCCTGGAGCATTACT
hDIS3rec     CACCAACCATTCAGCCAAGCAGTGCTGTCCTTCCTCCCCAAAATGCCATGGTCCATCACA
             ** ** ** **    ** ** ** **    ** ** ** ** ***** ***  *** **

               H  Q  P  F  S  Q  A  V  L  S  F  L  P  K  M  P  W  S  I  T
                                                miR1404
hDIS3        GAAAAGGACATGAAAAACCGAGAAGACCTGAGGCATCTGTGTATTTGTAGTGTAGACCCA
hDIS3rec     GAGAAAGATATGAAGAATAGGGAGGATCTCCGCCACCTCTGCATTTGTAGTGTAGACCCA
             ** ** ** ***** **  * ** ** **  * ** ** ** ******************

               E  K  D  M  K  N  R  E  D  L  R  H  L  C  I  C  S  V  D  P

hDIS3        CCAGGATGTACTGATAT...                                          (1457)
hDIS3rec     CCAGGATGTACTGATATTATATGACTC
             *****************

               P  G  C  T  D ...                                           (485)
```

Supplementary Figure S5. Synthetic inserts cloned into BI-16 vector. (**A**) Sequence of the *tri-miR2* insert. Red, blue, green and violet letters indicate *Eco*RI, *Sal*I, *Cla*I and *Hin*dIII restriction sites, used in cloning procedures; grey and black bacground indicate 5' and 3' miR flanking regions, respectively; red backgrounds correspond to 21 nt-long antisense target sequences (mature miRNA sequences), beginning in positions 1159., 1273. and 1404. of hDIS3 ORF (from 5' to 3'); violet backgrounds correspond to nucleotides 1-8 and 11-21 of the respective sense target sequences; green backgrounds indicate a 19 nt-long sequence derived from endogenous murine miR-155, with underlined 13 nt-long fragment able to form a loop within sh-miRNA structure; yellow background corresponds to HSV TK polyadenylation signal. (**B**) Alignment of the native (*hDIS3*) and recoded fragments (*hDIS3rec*) of hDIS3 ORF. Nucleotides unchanged during recoding are marked with asterisks; yellow backgrounds indicate *Sch*I restriction sites, utilized for excision of recoded hDIS3 insert from the synthetic construct; green backgrounds correspond to perfectly conserved flanking regions, enabling usage of the insert as a megaprimer in overlap PCR; red background indicate positions of sequences targeted by pre-designed sh-miRs.

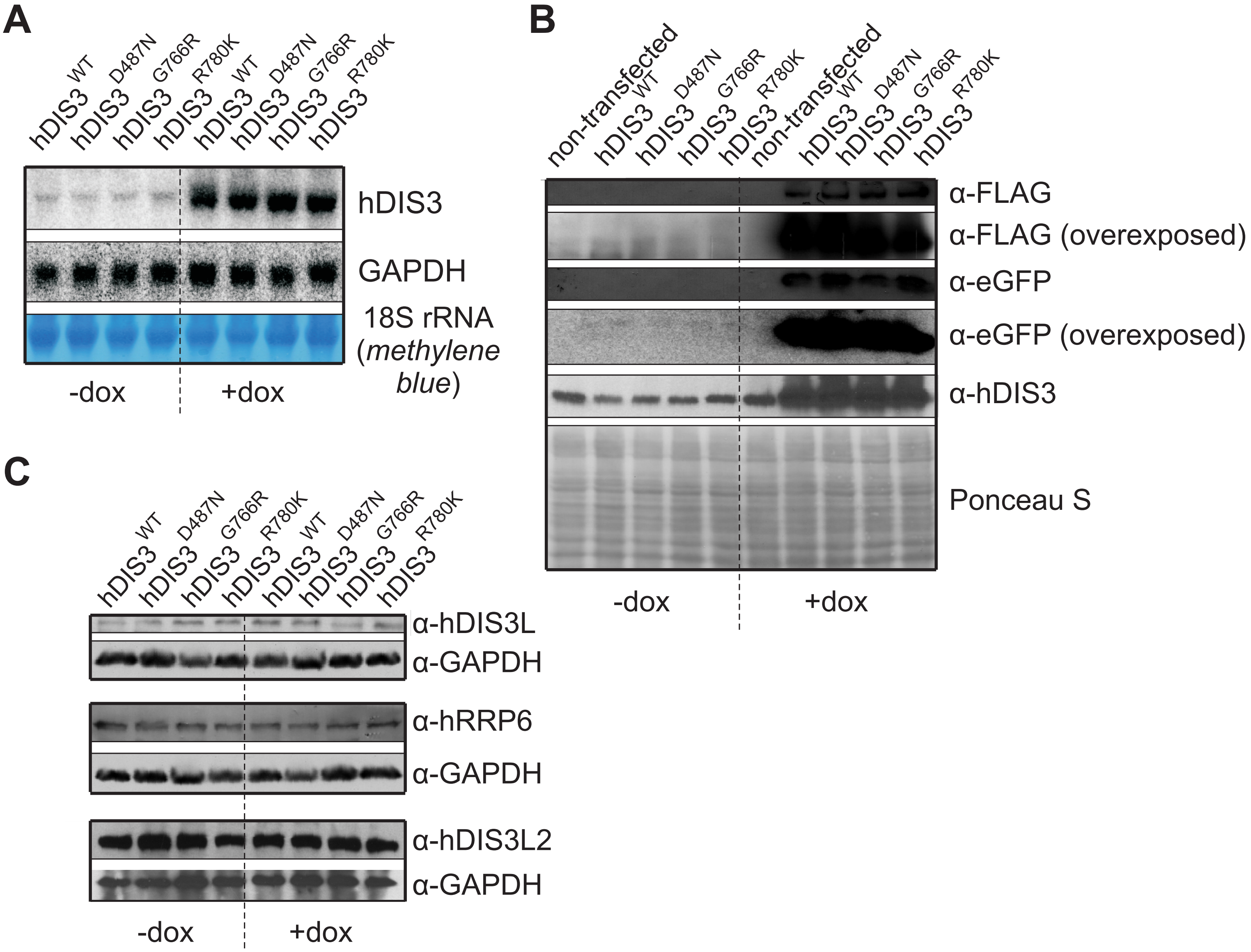

Supplementary Figure S6. Analysis of the expression of introduced exogenous inserts and 3'-5' exoribonucleases other than hDIS3 in constructed model cell lines. (**A**) Northern-blot analysis of *hDIS3* expression; total RNA was isolated from cell lines containing exogenous sequences coding for wild-type or mutated hDIS3 versions, either uninduced (lanes "-dox") or subjected to doxycycline treatment (lanes "+dox"); following electrophoretic separation in denaturing agarose gel, RNA was transferred onto membrane, which was then sequentially hybridized with probes complementary to the 5'-terminal part of hDIS3 ORF or to GAPDH (loading control). (**B**) Western-blot analysis of the expression of FLAG-tagged hDIS3 variants and eGFP-shmiR fusion; protein samples were prepared from the same cell lines as in (A) and, as a control, from non-transfected HEK293 Flp-In T-REx cells, separated in SDS-PAGE gel and transferred onto nitrocellulose membrane, which was then probed with anti-FLAG, anti-eGFP or anti-hDIS3 antibody; staining of the membrane with Ponceau S Red was utilized as a loading control. Prolonged exposures are shown to unequivocally demonstrate the lack of exogenous proteins in the absence of doxycycline. (**C**) Western-blot analysis of the expression of other human proteins displaying 3'-5' exoribonucleolytic activity; protein samples were analyzed as in (B), but using antibodies specific to hDIS3L, hRRP6 or hDIS3L2.

A

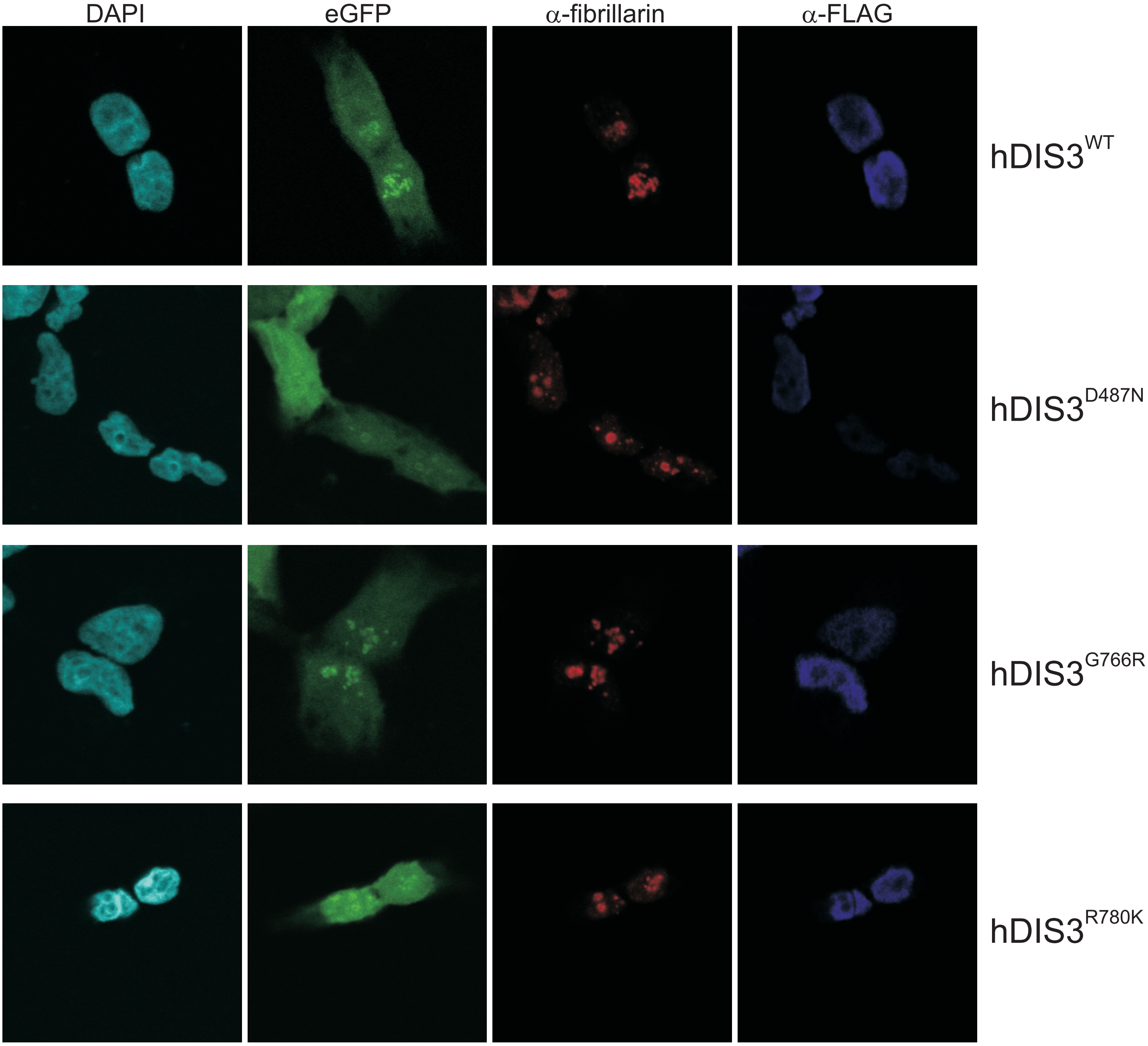
DAPI
eGFP
α-fibrillarin
α-FLAG
hDIS3WT
hDIS3D487N
hDIS3G766R
hDIS3R780K

B

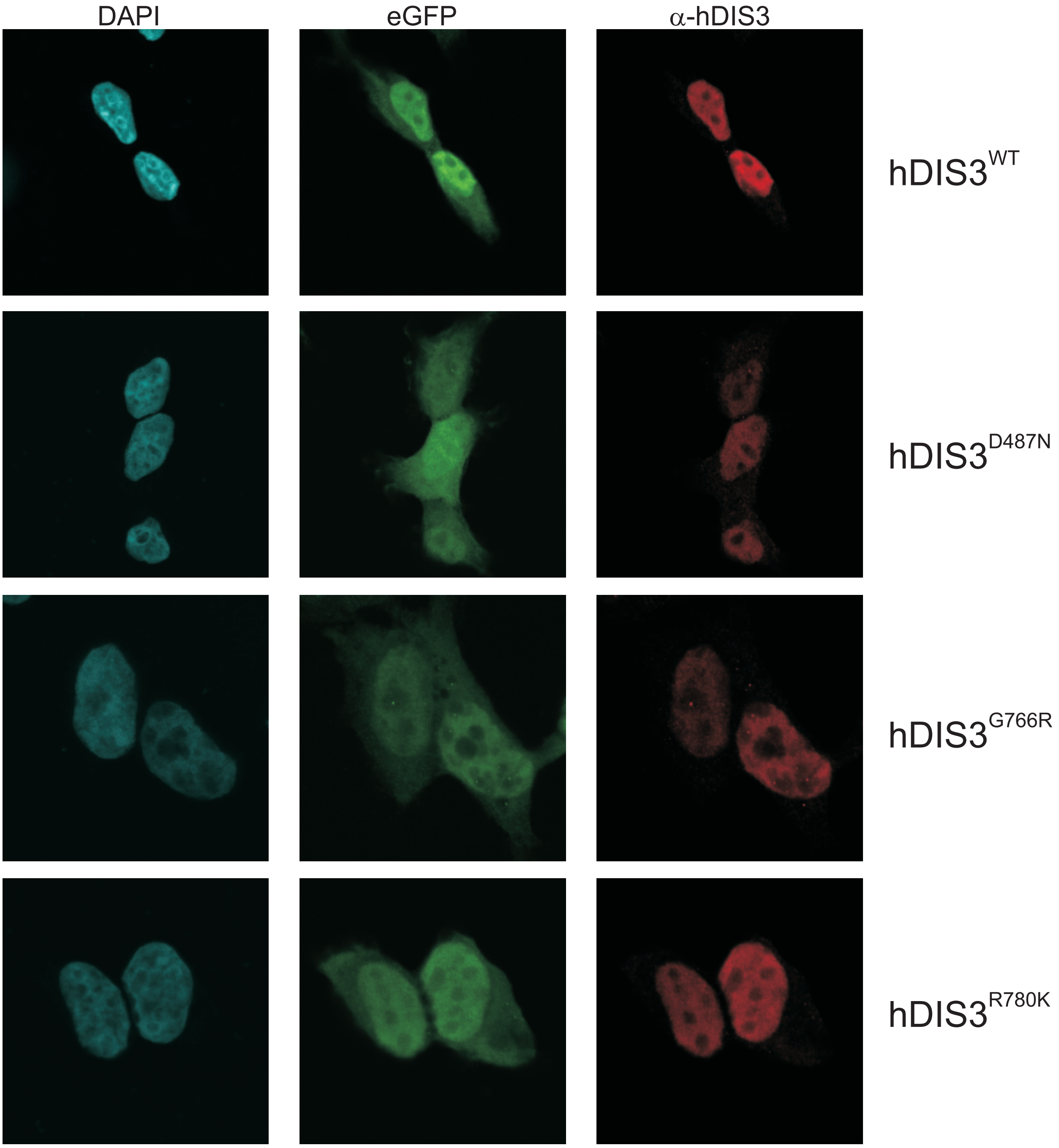

Supplementary Figure S7. Overexpressed exogenous hDIS3 variants display proper intracellular localization. (**A**) Model cell lines bearing exogenous sequences coding for different FLAG-tagged versions of hDIS3 were subjected to induction, followed by immunofluorescence using anti-FLAG and anti-fibrillarin (nucleolar marker) antibodies (detected with secondary antibodies coupled with Alexa Fluor 635 and 555 fluorescent dyes, respectively) in combination with DAPI staining of the nuclei; eGFP fluorescence was used to monitor the expression of eGFP-sh-miR fusion; hDIS3-FLAG fusions seemed to localize in the nucleoplasm, while they were clearly excluded from the nucleoli; weak cytoplasmic staining was also visible. (**B**) Cells were analyzed by immunofluorescence as above, but using only anti-hDIS3 antibody (detected with secondary antibody coupled with Alexa Fluor 555); a signal, corresponding to both endogenous and exogenous hDIS3, arose only from the nucleoplasm, but was absent from the nucleoli.

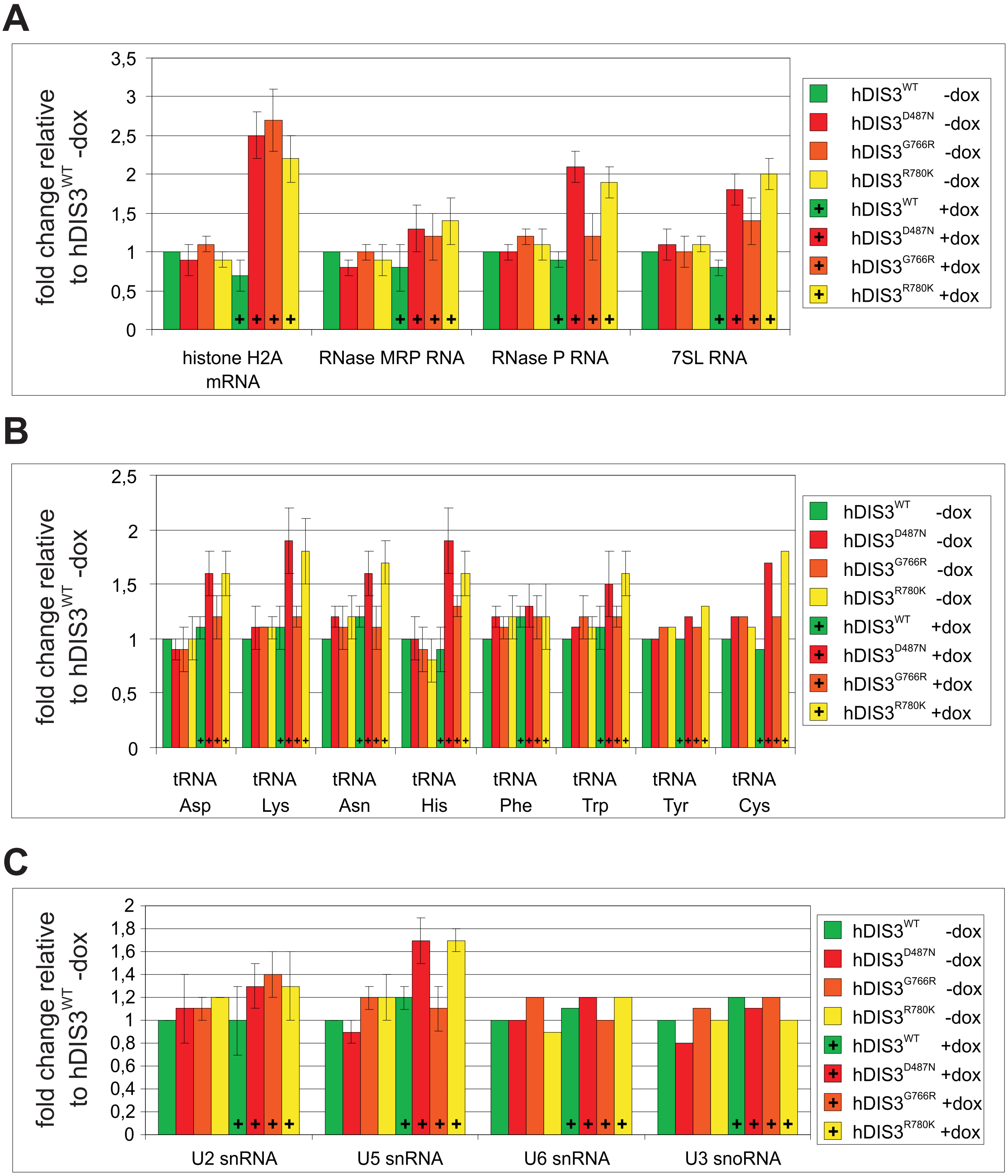

Supplementary Figure S8. Quantification of the results of northern blot experiments performed using total RNA isolated from various model cell lines expressing either wild-type or mutated hDIS3, either untreated ("-dox") or treated with doxycycline ("+dox") (related to Figure 5 in the main text). The graphs represent mean values (fold changes of RNA levels relative to the cell line with wild-type hDIS3, not subjected to induction with doxycycline) of two or three independent experiments; GAPDH mRNA (A) or 5S rRNA (B and C) was used for normalization purposes; error bars, reflecting standard deviation are given only in cases where independent hybridizations were done in triplicate. (**A**) Quantification of the data obtained for histone H2A mRNA and different RNA polymerase III transcripts (corresponding to the Figure 5A of the main text, (**B**) various tRNA molecules (corresponding to the Figure 5C) and (**C**) sn- and snoRNAs (corresponding to the Figure 5D).

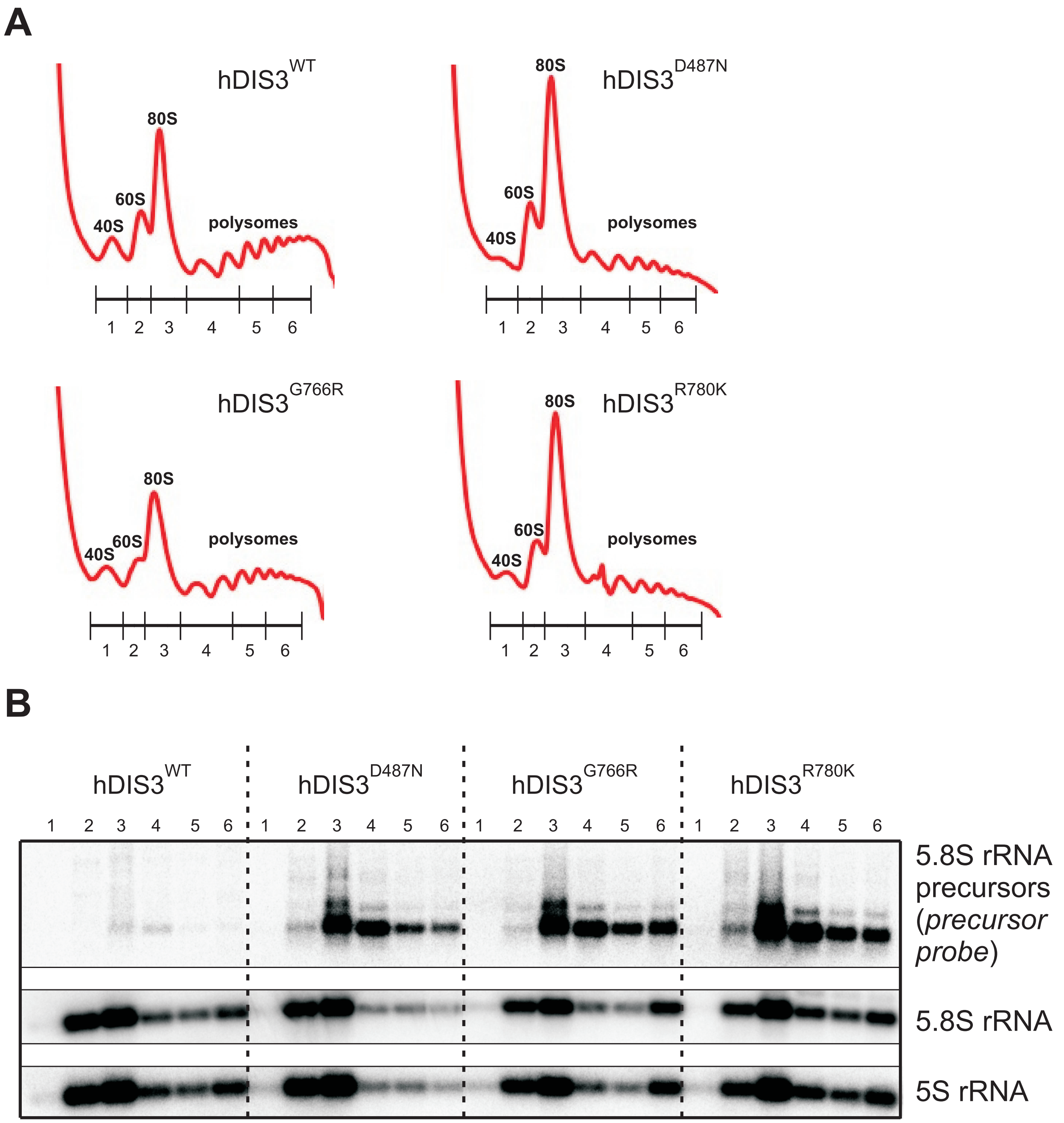

Supplementary Figure S9. Analysis of polysome profiles of model cell lines. (**A**) Native cytoplasmic extracts were prepared from stable cell lines subjected to induction of expression of exogenous hDIS3 variants and separated by centrifugation in sucrose gradients; graphs show distribution of absorbance at 254 nm from the top (*left*) to the bottom (*right*) of the gradients; peaks corresponding to individual ribosomal subunits (40S and 60S), monosomes (80S) and polysomes are indicated; 6 fractions were collected from each gradient (numbered 1-6) – their borders are marked with vertical lines; polysome profiles do not seem to vary between cell lines producing different variants of hDIS3. (**B**) Northern-blot analysis of RNA samples prepared from fractions collected as indicated in (A), performed using probes specific to 5.8S rRNA 3'-extended precursors and to the mature 5.8S and 5S rRNA molecules (controls); 5.8S rRNA precursors, which accumulate significantly in cell lines producing mutated hDIS3 variants, were present in polysomes.

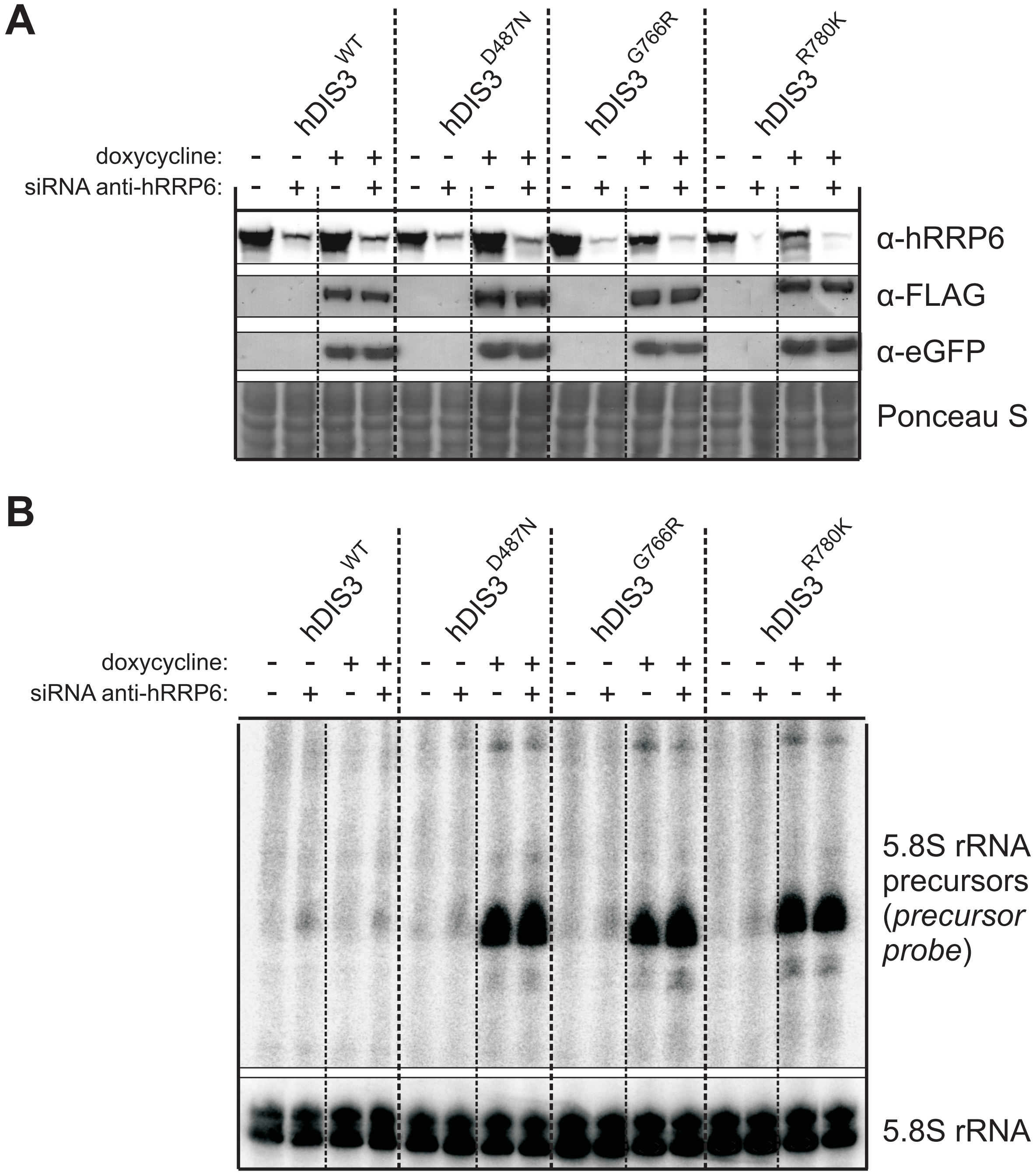

Supplementary Figure S10. siRNA-mediated silencing of *hRRP6* expression does not have a significant synergistic effect on accumulation of 5.8S rRNA precursors with production of mutated hDIS3 variants. (**A**) Western-blot analysis was performed for protein samples from model cell lines: untreated or treated with doxycycline (*doxycycline:* "-" or "+", respectively), which were transfected with either siRNA against hRRP6 (*anti-hRRP6:* "+") or with control, unrelated siRNA (*anti-hRRP6:* "-"); following transfer of proteins separated in SDS-PAGE onto nitrocellulose membrane, it was probed with antibodies specific to hRRP6, FLAG epitope or eGFP staining of the membrane with Ponceau S Red was employed as a loading control. (**B**) Northern-blot analysis of 5.8S rRNA precursors; total RNA was isolated from the same cell lines as in (A), separated in denaturing agarose gel and transferred onto nylon membrane, which was then hybridized with probes recognizing 5.8S rRNA 3'-extended precursors (*top*) or mature molecules (*bottom*).

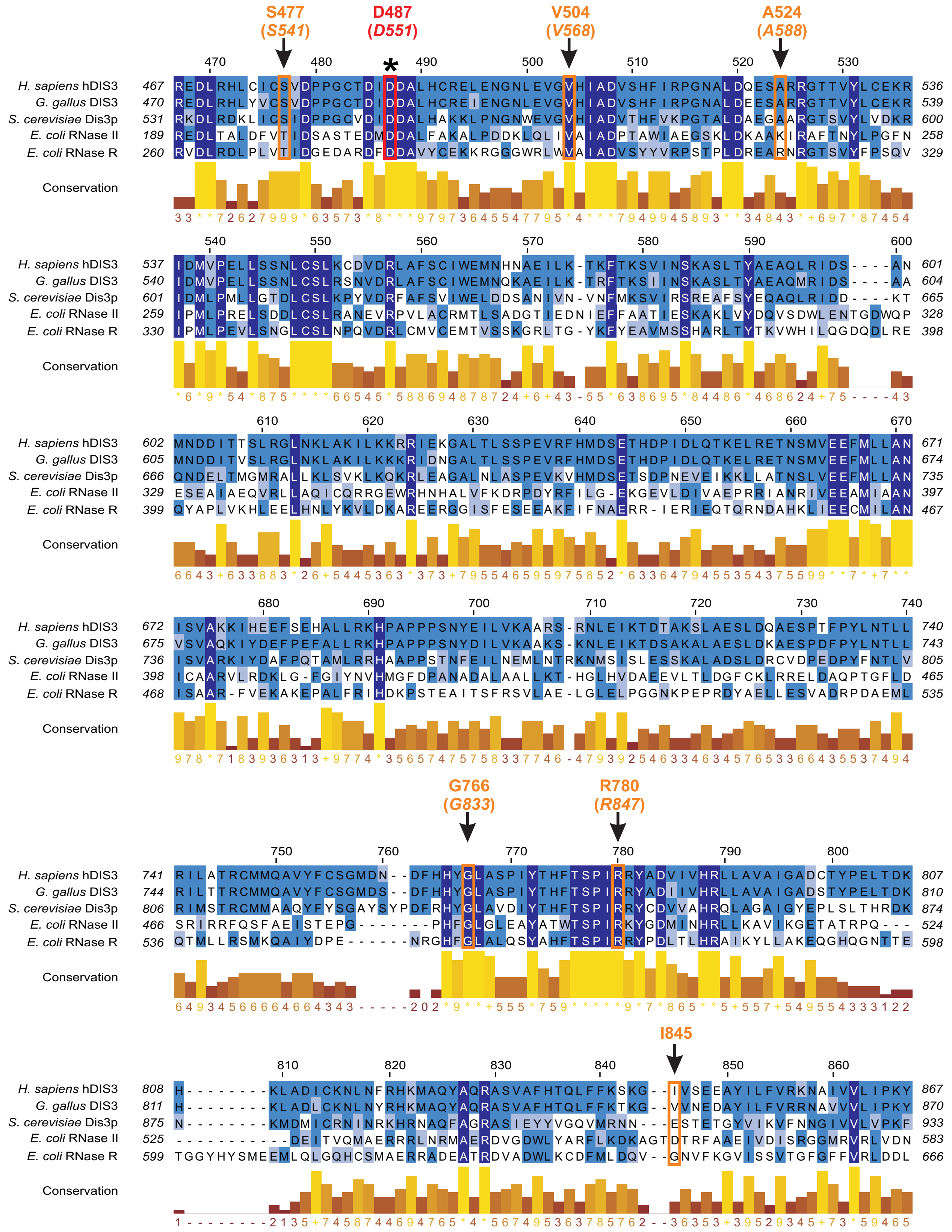

Supplementary Figure S11. Detailed amino acid sequence alignment of human, chicken and yeast DIS3 proteins with *E. coli* RNases II and R. Most mutations associated with multiple myeloma result in changes of relatively highly conserved aminoacids, except I845, which is not conserved in other members of the family and is localized slightly downstream the border of RNB domain. Amino acids substituted in MM are indicated in orange and their positions are shown with arrows. An asterisk indicates position of D487 (red lettering), which was previously shown to be critical for exoribonucleolytic activity of hDIS3. Equivalents in yeast Dis3 are italicized and in brackets.

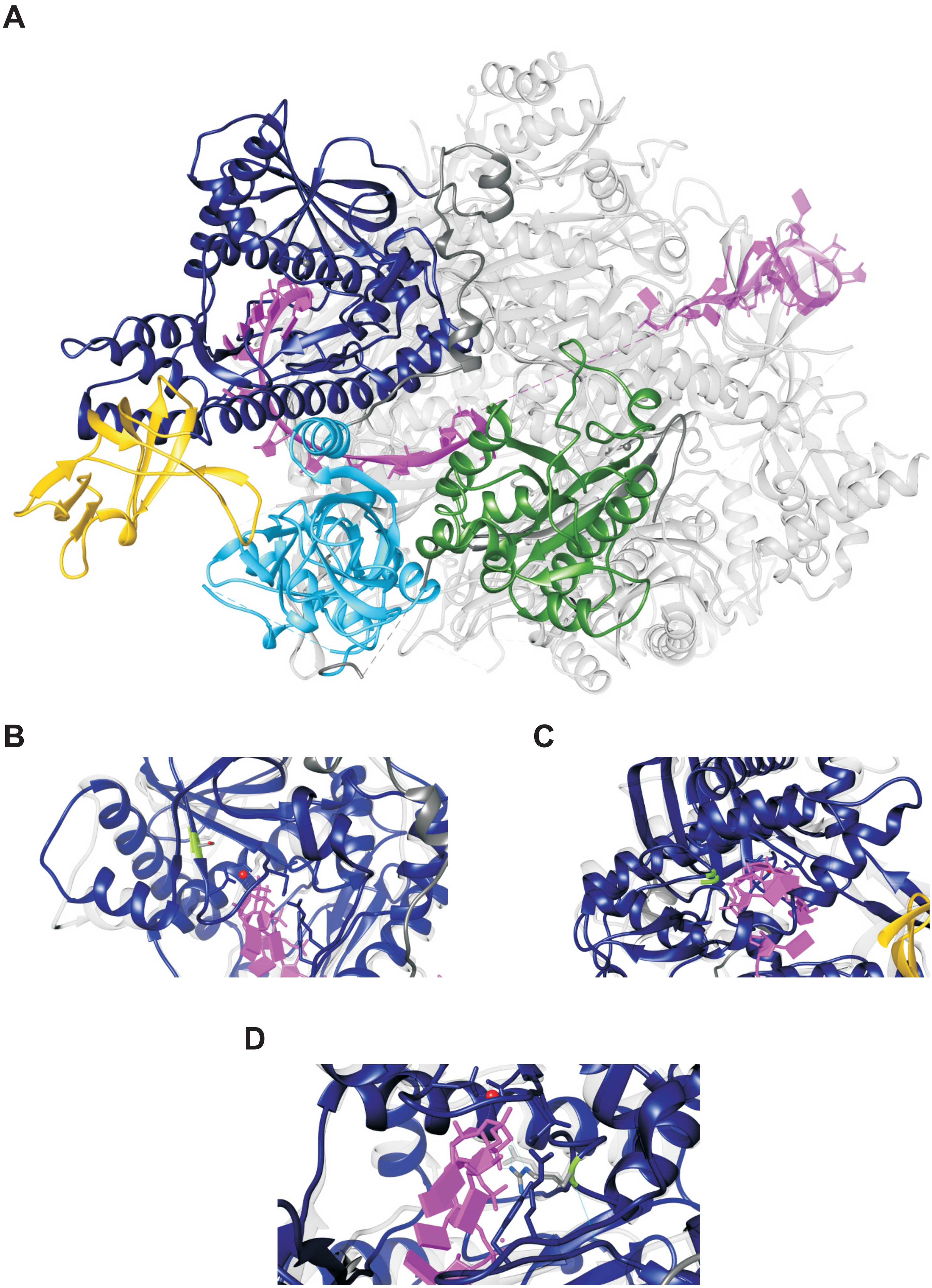
A
B
C
D

E

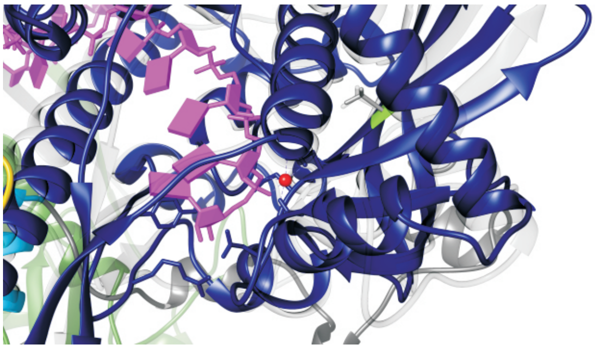

F

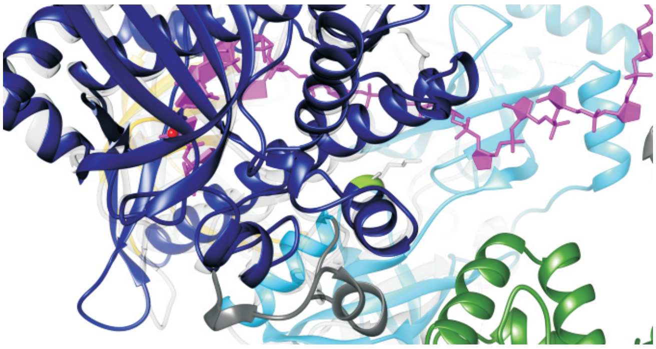

Supplementary Figure S12. Positioning of amino acids substituted by mutations found in MM patients. (**A**) Structure of the *S. cerevisiae* exosome complex, including its main catalytic subunit – Dis3, according to ref. 25. Exosome ring and Rrp6 subunit are coloured in light grey, RNA is in magenta. Dis3 domains are colored as follows: RNB domain – dark blue, PIN domain – green, S1 domain – yellow and 2 cold shock domains (CSDs) – cyan. Residues outside mentioned domains are in dark grey. (**B**)-(**F**) Yeast Dis3 residues, which are equivalent to residues mutated in MM patients. Dis3 protein is superimposed with *E. coli* RNase II structure to highlight the conserved structural regions. Dis3 domain are coloured as described in (A), RNase II is drawn in light grey, ribbon representation of mutated amino acids is in light green, atoms are colored according to elements (carbon – grey, oxygen – red, nitrogen – blue), magnesium ion is in red. (B) Substitution of S541 (analog of S477 in hDIS3) with a bulky amino acid (lysine) may affect the magnesium coordination. (C) Substitution of G833 in yeast Dis3 (analog of G766 in hDIS3) may potentially disrupt protein-RNA interactions. (D) Substitution of R847 in yeast Dis3 (analog of R780 in hDIS3), which takes part in RNA binding (hydrogen bonds as light blue lines), is likely to affect the efficiency of substrate cleavage. (E) and (F) V568 and A588 in yeast Dis3 (respective analogs of V504 and A524 in hDIS3) are located far from the active site and their influence on the enzymatic properties of the protein is difficult to predict.

Supplementary Tables

Table S1. Yeast strains used in this study.

**Strain**	**Genetic background**	**References**
ADZY532	[BMA64] *DIS3 WT*	this study
ADZY123	[BMA64] *dis3-D551N*	this study
ADZY531	[BMA64] *dis3-D171N*	ref. 24
Y11777	[BY4742] *rrp6Δ*	Euroscarf
ADZY679	[BMA64] *dis3-V568G*	this study
ADZY681	[BMA64] *dis3-A588P*	this study
ADZY783	[BMA64] *dis3-G833R*	this study
ADZY685	[BMA64] *dis3-R847K*	this study
ADZY732	*rrp6Δ dis3-G833R*	this study
ADZY742	*rrp6Δ dis3-R847K*	this study
ADZY713	*DIS3 WT / dis3-D171N G833R*	this study
ADZY716	*DIS3 WT / dis3-D171N R847K*	this study

Table S2. Oligonucleotides used in this study. "***r***" before sequence in brackets indicates that the oligo is composed of ribonucleotides.

Oligonucleotide	Sequence (5'-3')	Purpose
S477Rfor	*TGTGTATctgcagAGTAGACC*CACCAGGATGTACTGATATAG	site-directed mutagenesis (hDIS3 S477R)
S477Rrev	*GGTCTACTctgcagATACACA*GATGCCTCAGGTCTTCTCGG	site-directed mutagenesis (hDIS3 S477R)
V504Gfor	*AGGTTGGtggccaTATTGCT*GATGTGAGCCATTTTATTAGG	site-directed mutagenesis (hDIS3 V504G)
V504Grev	*AGCAATAtggccaCCAACCT*CCAAATTTCCATTTTCGAGTTC	site-directed mutagenesis (hDIS3 V504G)
A524Pfor	*AGAATCAcctaggAGAGGAA*CAACTGTGTATCTTTGTGAAAAG	site-directed mutagenesis (hDIS3 A524P)
A524Prev	*TTCCTCTcctaggTGATTCT*TGATCCAAGGCATTTCCTGGC	site-directed mutagenesis (hDIS3 A524P)
G766Rfor	*CATCACTaccggtTAGCGTC*TCCAATATACACACATTTTAC	site-directed mutagenesis (hDIS3 G766R)
G766Rrev	*GACGCTAaccggtAGTGATG*AAAATCATTATCCATTCCAGAAC	site-directed mutagenesis (hDIS3 G766R)
R780Kfor	*CCCATTAaacgttACGCAGA*TGTCATTGTTCATCGGCTTTTGG	site-directed mutagenesis (hDIS3 R780K)
R780Krev	*TCTGCGTaacgttTAATGGG*TGAAGTAAAATGTGTGTATATTGG	site-directed mutagenesis (hDIS3 R780K)
I845Vfor	*CAAAGGAGTAGTAAGTGAAGaggcctATATTTT*ATTTGTAAGAAAGAATGCC	site-directed mutagenesis (hDIS3 I845V)
I845Vrev	*AAAATATaggcctCTTCACTTACTACTCCTTTG*CTTTTGAAGAATAACTGGG	site-directed mutagenesis (hDIS3 I845V)
D3PINF	*AGGAATAAccggGCGATTCGA*GTAGCAGCAAAATGGTACAATG	site-directed mutagenesis (hDIS3 D146N)
D3PINR	*TCGAATCGCccggTTATTCCT*GTCATTAGCATTTTCTCCCTG	site-directed mutagenesis (hDIS3 D146N)

ADZKD106	CGGTCATATGAGAGTGTGTTGCG	construction of yeast strains
ADZKD107	AGTGGTTTAGTGGTAAAATCCAACGTTGCCATCGTTGGGCCCCCGGTTCG	construction of yeast strains
ADZKD133	AACGGTAATTGGGAAGTTGG**tggcca**TATTGCTGATGTTACTCACT	construction of *S. cerevisiae dis3-V568G* strain
ADZKD134	AGTGAGTAACATCAGCAATA**tggcca**CCAACTTCCCAATTACCGTT	construction of *S. cerevisiae dis3-V568G* strain
ADZKD135	GCACTGCCCTGGATGCGGAA**gggccc**GCAAGAGGTACTTCTGTATA	construction of *S. cerevisiae dis3-A588P* strain
ADZKD136	TATACAGAAGTACCTCTTGC**gggccc**TTCCGCATCCAGGGCAGTGC	construction of *S. cerevisiae dis3-A588P*strain
ADZKD137	TATCCTGACTTTAGACACT**accggt**TAGCCGTTGATATCTACACA	construction of *S. cerevisiae dis3-G833R* strain
ADZKD138	TGTGTAGATATCAACGGCTA**accggt**AGTGTCTAAAGTCAGGATA	construction of *S. cerevisiae dis3-G833R* strain
ADZKD139	CACATTTCACATCACCTATTA**aacgtt**ACTGTGATGTTGTGGCCCAT	construction of *S. cerevisiae dis3-R780K* strain
ADZKD140	ATGGGCCACAACATCACAGT**aacgtt**TAATAGGTGATGTGAAATGTG	construction of *S. cerevisiae dis3-R780K* strain
ADZKD141	CCTAAATAGAGCATTCAACGGTGACCAGG	construction of *S. cerevisiae dis3-D171N G833R(R780K)-pA/ DIS3 WT strains*
ADZKD142	CCTGGTCACCGTTGAATGCTCTATTTAGG	construction of *S. cerevisiae dis3-D171N G833R(R780K)-pA/ DIS3 WT strains*
ADZKD145	GGATGATGTTAATTGCTTGG	verification of yeast strains
ADZKD146	TTGAAACTCTACCACCGACC	verification of yeast strains
D3FMluI	atat**acgcgt***GCCGCCACC***ATG**CTCAAGTCCAAGACGTTC	cloning of hDIS3 ORF into BI-16 vector backbone
D3RB120I	gcgc**gggccc***TTA***CTTGTCGTCGTCGTCCTTGTAATCTATATC**TTTTCCAAGCTTCATCTTCT	cloning of hDIS3 ORF into BI-16 vector backbone
eGFPFor	gcg**gaattc**catatac**ctagg***ACC***ATG**GTGAGCAAGGGCGAGGAGC	cloning of eGFP ORF into [pUCAmpMinusMCS] vector backbone

eGFPRev	gcgc**gtcgac**TCACTACCTCCTC*TTA*CTTGTACAGCTCGTCCATGC	cloning of eGFP ORF into [pUCAmpMinusMCS] vector backbone
CMV_F	TACGAAGTTATTGATCAGGGCTAGCCTAGGGGGCCAGATATAC CGTTGAC	cloning of hDIS3 expression cassette into pLoxPuro/pLoxBsr
BGH_R	GGCTCACCTCGAGGATCTGGGCTAGCCTAGGGCCATAGAGCCC ACCGCATC	cloning of hDIS3 expression cassette into pLoxPuro/pLoxBsr
DIS3_LF	GTTGTTAGTGGTGTACGTAGGCGGCCGCTCTGGCATTTACCTC CAAGG	cloning of chicken *DIS3* left arm into pLoxPuro/pLoxBsr
DIS3_LR	GGTAGGGGATCCACTAGTTCTAGAGCGGCCGTCTATCAATGGC ATTGGACTAGATGGCCT	cloning of chicken *DIS3* left arm into pLoxPuro/pLoxBsr
DIS3_RF	TAGGGCGAATTGGAGCTCCACCGCGGTGGCGGCCCGGCCGGC CCTGTTGTTTTCCTCAGTTAC	cloning of chicken *DIS3* right arm into pLoxPuro/pLoxBsr
DIS3_RR	GGTTCCTTGGAGGTAAATGCCAGAGCGGCCGCCTACGTACACC ACTAACAACAAAGG	cloning of chicken *DIS3* right arm into pLoxPuro/pLoxBsr
RTADZ-9	GAGATACATTGTGAGGGACC	sequencing of yeast *DIS3*
RTADZ-27	ATGTCAGTTCCCGCTATCGC	sequencing of yeast *DIS3*
ADZKD151	CGTCGTTCTTGTTACCAACG	sequencing of yeast *DIS3*
ADZKD152	CACCGTGATTTCCGACAAGC	sequencing of yeast *DIS3*
ADZ1601	GCCCGCAGAAGGCCACGATTGG	sequencing of yeast *DIS3*
RTADZ-68	AGGGCTCTCTTGAAATTGTCTG	sequencing of yeast *DIS3*
ADZ1603	GACAGGTGTGTGGATCCCGAAG	sequencing of yeast *DIS3*
HD3F883	GAAGATATTGTGGCTGTGGAGC	sequencing of hDIS3 inserts (except those containing recoded fragment)
HD3F1848	CCGTGGACTGAATAAACTAGCC	sequencing of hDIS3 inserts
HD3F2429	TGACAGACAAACACAAGCTTGC	sequencing of hDIS3 inserts
HD3R1021	TTACAGCAGTCTTAAGCATTCG	sequencing of hDIS3 inserts (except those containing recoded fragment)
HD3R1592	CTGGCTGATTCTTGATCCAAGG	sequencing of hDIS3 inserts
HD3R2443	TGTGTTTGTCTGTCAACTCTGG	sequencing of hDIS3 inserts
hD3r819R	CTTGTTCTCCTCGGAATCTC	sequencing of hDIS3 inserts containing recoded fragment

RSZ174	TCGTTTAGTGAACCGTCAG	sequencing of hDIS3 insert in pLoxPuro/pLoxBsr backbone
RSZ176	GGAGTGGCACCTTCCAG	sequencing of hDIS3 insert in pLoxPuro/pLoxBsr backbone
DIS3_9f	CTGAGAAGATATGTAAATGCTG	sequencing of *hDIS3* gene in multiple myeloma cell lines
DIS3_10f	ATATGTTGTAGTTGTGCTTTG	sequencing of *hDIS3* gene in multiple myeloma cell lines
DIS3_11r	CTTGTTATTTGAACCACTCG	sequencing of *hDIS3* gene in multiple myeloma cell lines
DIS3_17f	GTTTCTGGCTACTACTTCTAC	sequencing of *hDIS3* gene in multiple myeloma cell lines
DIS3_18r	GGCTTCTTCACTTACTATTC	sequencing of *hDIS3* gene in multiple myeloma cell lines
DIS3_19r	TAGGGCAAAACTTTACATGG	sequencing of *hDIS3* gene in multiple myeloma cell lines
SumoF	TCATACTGTCAAAGACAGGG	sequencing of hDIS3 inserts in the pET-28M-6xHis-SUMOTag backbone
BI16seq1	CATTCTCCGCTCCATCGTTC	sequencing of *eGFP-tri-miR* 2 inserts in the BI-16 vector backbone
BI16seq2	TCCACTGGTCGACTCACTAC	sequencing of *eGFP-tri-miR* 2 inserts in the BI-16 vector backbone
CMV_R	ATGTAACGCGGAACTCCAT	sequencing of hDIS3 expression cassette in pLoxPuro/pLoxBsr backbone
ss17-A_{14}	r(CCCCACCACCAUCACUUAAAAAAAAAAAAAA)	RNA oligonucleotide substrate used in biochemical assays
ss44	r(CGACUGGAGCACGAGGACACUGACAUGGACUGAAGGAGUAGAAA)	RNA oligonucleotide substrate used in biochemical assays

compl	r(AAGUGAUGGUGGUGGGG)	RNA oligonucleotide used in biochemical assays
GAPDH (Forward/Reverse)	GTCAGCCGCATCTTCTTTTG / GCGCCCAATACGACCAAATC	qPCR primer pair
40-2b PROMPT (Forward/Reverse)	GGGAGTCTAAGGAAAAGGAG / CAGTGAAAGGAGAGCGTATC	qPCR primer pair
40-13 PROMPT (Forward/Reverse)	GGAAATAGTGGAGAAAAGCA / CATTTTTGAAGGAACGGTAG	qPCR primer pair
40-33 PROMPT (Forward/Reverse)	CTGGCCTAGCTAAAGTCTCA / TCTGCTCCTAGCTCTCAGTC	qPCR primer pair
40-52 PROMPT (Forward/Reverse)	AGTTCCAAGAAACCACACAC / GGTCGTTTGAGTGGACTAAC	qPCR primer pair
40-13 gene (Forward/Reverse)	GGAGTTGACAGCAGAGTTTT / ATGCACTTTAACCAGGTTTG	qPCR primer pair
40-33 gene (Forward/Reverse)	GGTGACAACTGGTCTCTGTC / CCGAAAGTTACCAAAACATT	qPCR primer pair
40-52 gene (Forward/Reverse)	AAAATGAGACTGGCCACTAA / GATGTGGGATTCTCTCAAAC	qPCR primer pair
mtATP6/8 (Forward/Reverse)	CCATCAGCCTACTCATTCAACC / GCGACAGCGATTTCTAGGATAG	qPCR primer pair
oligo(dT) primer	TTTTTTTTTTTTTTTTTT	primer for reverse transcription
DIS3_rfinR	ACACAACTGTAACACCTGCA	synthesis of PCR probe for Southern hybridization
GAPDH_F	TGCACCACCAACTGCTTAGC	synthesis of PCR probe for northern-blot
GAPDH_R	GGCATGGACTGTGGTCATGAG	synthesis of PCR probe for northern-blot
7SL_F	TCGGGTGTCCGCACTAAGTT	synthesis of PCR probe for northern-blot
7SL_R	TGGCTATTCACAGGCGCGAT	synthesis of PCR probe for northern-blot
yeast 7S antisense oligo	GGCCAGCAATTTCAAGTTA	probe for northern-blot
yeast 5.8S antisense oligo	GCGTTGTTCATCGATGC	probe for northern-blot

yeast 5S antisense oligo	CTACTCGGTCAGGCTC	probe for northern-blot
human 5.8S rRNA precursor antisense oligo	GCGATTGATCGGCAAGCGA	probe for northern-blot
human 5.8S rRNA antisense oligo	TCCTGCAATTCACATTAATTCTCGCAGCTAGC	probe for northern-blot
human 5S rRNA antisense oligo	CATCCAAGTACTAACCAGGCCC	probe for northern-blot
human tRNA Asn^{GTT} antisense oligo	ACCAACCTTTCGGTTAACAGCCGAACGCGC	probe for northern-blot
human tRNA Asp^{GTC} antisense oligo	CGGTCTCCCGCGTGACAGGCGGGGATACTC	probe for northern-blot
human tRNA His^{GTG} antisense oligo	CGAGGTTGCTGCGGCCACAACGCAGAGTAC	probe for northern-blot
human tRNA Trp^{CCA} antisense oligo	CGCAACCTTCTGATCTGGAGTCAGACGCGC	probe for northern-blot
human tRNA Tyr^{GTA} antisense oligo	GACCTAAGGATCTACAGTCCTCCGCTCTAC	probe for northern-blot
human tRNA $Lys^{TTT\ (1,2)}$ antisense oligo	GACCCTCAGATTAAAAGTCTGATGCTCTAC	probe for northern-blot
human tRNA Phe^{GAA} antisense oligo	GGACCTTTAGATCTTCAGTCTAACGCTCTC	probe for northern-blot
human tRNA Cys^{GCA} antisense oligo	GGGACCTCTTGATCTGCAGTCAAATGCTCT	probe for northern-blot
human RNase P RNA antisense oligo	ATGGGCGGAGGAGAGTAGTCTG	probe for northern-blot
human RNase MRP RNA antisense oligo	GCCGCGCTGAGAATGAGCCCC	probe for northern-blot
human U2 snRNA antisense oligo	GGGTGCACCGTTCCTGGAGGTACTGCAATA	probe for northern-blot
human U5 snRNA antisense oligo	TTGGGTTAAGACTCAGAGTTGTTCCTCTCC	probe for northern-blot

human U6 snRNA antisense oligo	GAACGCTTCACGAATTTGCG	probe for northern-blot
human U3 snoRNA antisense oligo	ACCACTCAGACCGCGTTCTCTCCCTCTCAC	probe for northern-blot

Table S3. Plasmids used in this work.

**Plasmid**	**Genotype**	**Source**
pHEX1	[pET-28M-6xHis-SUMOTag] *hDIS3 WT*	ref. 26
pHEX8	[pET-28M-6xHis-SUMOTag] *hDIS3 RNB MUT (D487N)*	ref. 26
pMM1	[pET-28M-6xHis-SUMOTag] *hDIS3 S477R*	this study
pMM2	[pET-28M-6xHis-SUMOTag] *hDIS3 V504G*	this study
pMM3	[pET-28M-6xHis-SUMOTag] *hDIS3 A524P*	this study
pMM4	[pET-28M-6xHis-SUMOTag] *hDIS3 G766R*	this study
pMM5	[pET-28M-6xHis-SUMOTag] *hDIS3 R780K*	this study
pMM6	[pET-28M-6xHis-SUMOTag] *hDIS3 I845V*	this study
pMM7	[BI-16’’] *hDIS3rec WT; eGFP-tri-miR 2/HSV-TK-pA*	this study
pMM8	[BI-16’’] *hDIS3rec RNB MUT (D487N); eGFP-tri-miR 2/HSV-TK-pA*	this study
pMM9	[BI-16’’] *hDIS3rec G766R; eGFP-tri-miR 2/HSV-TK-pA*	this study
pMM10	[BI-16’’] *hDIS3rec R780K; eGFP-tri-miR 2/HSV-TK-pA*	this study
pMM11	[BI-16’’] *hDIS3rec D146N; eGFP-tri-miR 2/HSV-TK-pA*	this study
pMM12	[BI-16’’] *hDIS3rec D146N D487N; eGFP-tri-miR 2/HSV-TK-pA*	this study
pMM13	[BI-16’’] *hDIS3rec D146N G766R; eGFP-tri-miR 2/HSV-TK-pA*	this study
pMM14	[BI-16’’] *hDIS3rec D146N R780K; eGFP-tri-miR 2/HSV-TK-pA*	this study
pMM15	[pLoxPuro] *hDIS3; cDIS3del*	this study
pMM16	[pLoxBsr] *hDIS3; cDIS3del*	this study

Supplementary References

61. Puig,O., Caspary,F., Rigaut,G., Rutz,B., Bouveret,E., Bragado-Nilsson,E., Wilm,M. and Séraphin,B. (2001) The tandem affinity purification (TAP) method: a general procedure of protein complex purification. Methods, 24, 218-229.

62. Bryksin,A.V. and Matsumura,I. (2010) Overlap extension PCR cloning: a simple and reliable way to create recombinant plasmids. Biotechniques, 48, 463-465.

63. Arakawa,H., Lodygin,D. and Buerstedde,J.M. (2001) Mutant loxP vectors for selectable marker recycle and conditional knock-outs. BMC Biotechnol., 1, 7.

64. Song,J., Liang,C. and Chen,X. (2006) Transduction of avian cells with recombinant baculovirus. J. Virol. Methods, 135, 157-162.

65. Li,M.Z. and Elledge,S.J. (2007) Harnessing homologous recombination *in vitro* to generate recombinant DNA via SLIC. Nat. Methods, 4, 251-256
